# Development of a highly sensitive platform for protein–protein interaction detection and regulation of T cell function

**DOI:** 10.1073/pnas.2318190121

**Published:** 2024-08-06

**Authors:** Hideki Hayashi, Tak Wah Mak, Yoshimasa Tanaka, Yoshinao Kubo, Mai Izumida, Ryuji Urae, Toshifumi Matsuyama

**Affiliations:** ^a^Medical University Research Administrator, Nagasaki University School of Medicine, Nagasaki 852-8523, Japan; ^b^Centre for Oncology and Immunology, Hong Kong Science Park, Hong Kong Special Administrative Region 999077, China; ^c^Center for Medical Innovation, Nagasaki University, Nagasaki 852-8588, Japan; ^d^Princess Margaret Cancer Center, University Health Network, Toronto, ON M5G 2M9, Canada; ^e^Department of Pathology, School of Clinical Medicine, Li Ka Shing Faculty of Medicine, The University of Hong Kong, Hong Kong Special Administrative Region 999077, China; ^f^Department of Clinical Medicine, Institute of Tropical Medicine, Nagasaki University, Nagasaki 852-8523, Japan; ^g^Souseikai Clinical Research Center, Fukuoka 812-0025, Japan; ^h^Department of Forensic Pathology and Science, Nagasaki University Graduate School of Biomedical Sciences, Nagasaki University, Nagasaki 852-8523, Japan

**Keywords:** interferon signaling, interferon-gamma, protein interaction, IL-2, T cell activation

## Abstract

We developed a highly sensitive assay for detecting protein–protein interaction using chimeric receptors comprising two molecules of interest in the extracellular domain and interferon alpha and beta receptor subunit 1 or 2 (IFNAR1/2) in the intracellular domain. This intracellular IFNAR1/2 reconstitution system (IFNARRS) proved markedly more sensitive than the NanoBiT system, currently considered one of the best detection systems for protein interaction. Employing chimeric receptors with extracellular domains from the IFNγ or IL-2 receptor and the intracellular domains of IFNAR1/2, the IFNARRS system effectively identifies low IFNγ or IL-2 levels. Cells stably expressing these chimeric receptors responded to IFNγ secreted by activated T cells following various stimuli, including a specific peptide-antigen. The activation signals were further enhanced by the expression of relevant genes, such as costimulators, via IFN-stimulated response elements in the promoters. Besides IFNγ or IL-2, the IFNARRS system demonstrated the capability to detect other cytokines by using the corresponding extracellular domains from these target cytokine receptors.

## Significance

To detect protein–protein interaction, we used chimeric receptors comprising two molecules of interest in the extracellular domain and interferon alpha and beta receptor subunit 1 or 2 (IFNAR1/2) in the intracellular domain. High sensitivity of this IFNAR1/2 reconstitution system (IFNARRS) was achieved by employing a robust and specific IFN-stimulated response element (ISRE) in the promoter of Nluc reporter gene. In addition to detecting various protein interactions, IFNARRS was used to detect low IFNγ or IL-2 levels. Cells stably expressing the chimeric receptors responded to IFNγ secreted by activated T cells. The cells further used the activation signals to confer specific functions upon themselves and the cocultured T cells by activating appropriate genes such as costimulators, via ISRE.

Various extracellular and intracellular signaling mechanisms are mediated via specific interactions between two or several molecules. An engineered luciferase, NanoLuc (Nluc), is widely used as reporter of various biological signals due to its small molecular mass and robust enzymatic activity (1). To detect protein–protein interactions, a complementation reporter (NanoBiT) was developed using a two-subunit system consisting of large (LgBiT) and small (SmBiT) subunits (2). These subunits are fused to the proteins of interest. Upon protein binding, the subunits come into proximity to form a functional enzyme that exerts a luminescent signal. The NanoBiT system is one of the most sensitive methods for detecting protein interactions. Type I interferons (such as IFNα and β) trigger dimerization of interferon alpha and beta receptor subunits 1 and 2 (IFNAR1/2) and activate the JAK-STAT pathway and the downstream interferon-stimulated genes (ISGs) (3–5). Activated STAT1/STAT2 complex with IRF9 binds to a specific region of promoters called IFN-stimulated response element (ISRE) in numerous ISGs. Activation of genes via ISREs is strong and specific to type I IFNs (3–7) (*SI Appendix*, Fig. S7). As activation is enhanced with increasing numbers of ISRE, we sought to detect protein interactions using chimeric receptors comprising two molecules of interest in the extracellular (EX) domains that are fused to the transmembrane (TM) and intracellular (IN) domains of IFNAR1 and IFNAR2. When the interaction between human EGFR-related 2 (HER2) and its specific affibody (8, 9) was determined using a reporter gene in which 10xISRE was placed in the promoter region of Nluc, the intensity of the signal was 20-fold or greater than that of the NanoBiT system. Notably, the signal was specific to protein binding as endogenous IFNAR1 was silenced. Beyond the binding assay, IFNARRS (IFNAR1/2 reconstitution system) was used to detect ligand-dependent receptor clustering when the EX domains of the IFNGR1/2 (10, 11) or IL2Rβ /γ (12, 13) subunits were fused to the IFNAR1/2 TM and IN domains. HEK293T cells stably expressing chimeric receptors detected IFNγ or IL-2 secreted from activated T cells (10–14). Furthermore, the ligand-dependent signals activated relevant genes via ISRE to confer specific functions upon themselves and the cocultured cells.

The IFNARRS can be applied to the detection of various cytokines besides IFNγ or IL-2. In fact, when a target cytokine receptor is composed of two or three molecules whose EX regions recognize the cytokine, the cells expressing the chimeric receptors composed of the EX domains of the target cytokine receptor fused to the TM and IN domains of IFNAR1/2 can detect and modify the target cytokine-producing cells by activating the appropriate genes via ISRE. More precise targeting can be achieved by expressing an appropriate membrane-anchored ZZ affibody (ZZ-TM) that binds to specific antibodies via their Fc region to the target cell surface molecules (15).

## Results

### Extracellular Domains of IFNAR1/2 Can be Replaced with the Proteins of Interest.

**Fig. 1.**
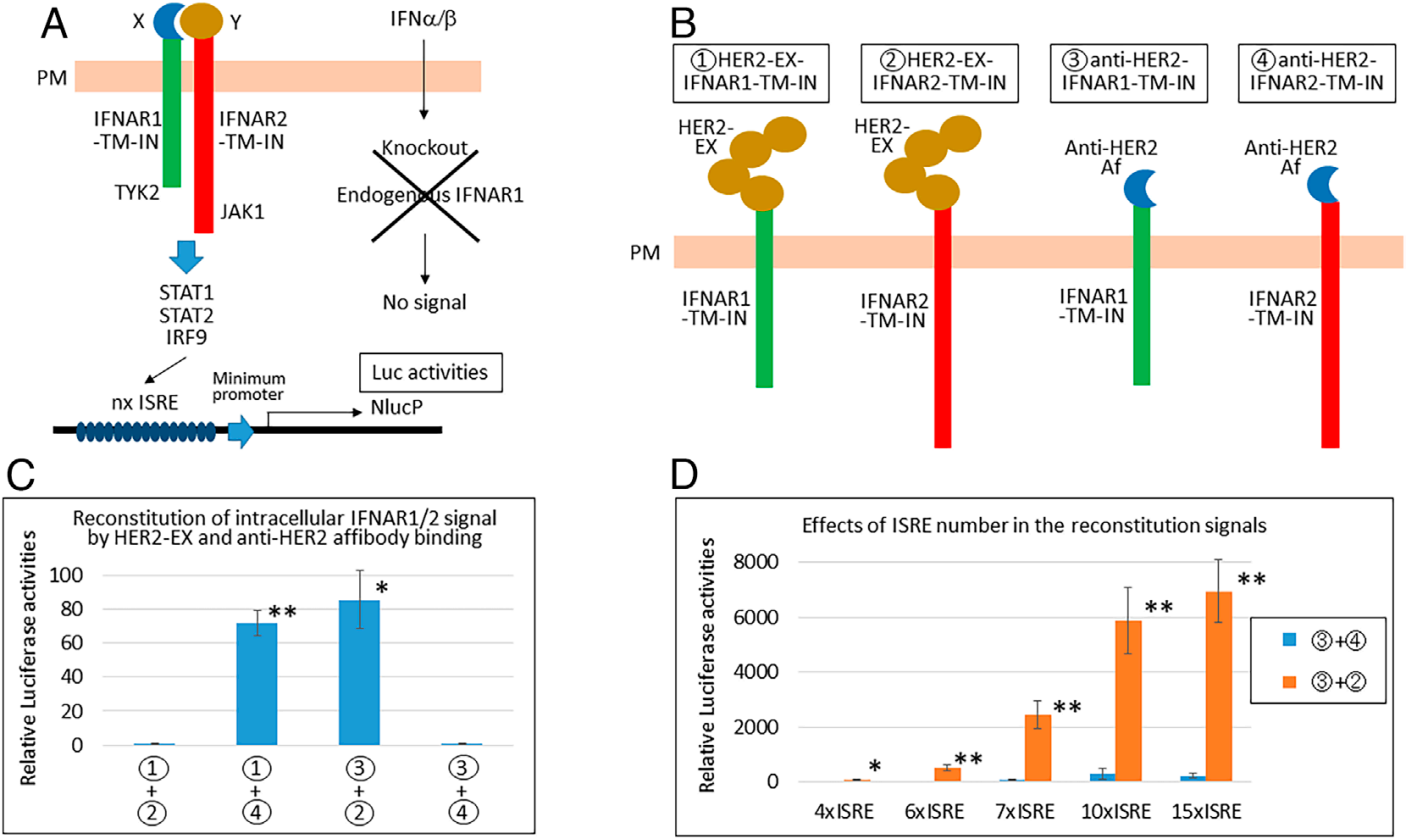
Specific binding of the extracellular domain of HER2 and the anti-HER2 affibody was detected as Nluc activity. (*A*) Schematic illustration of the IFNAR1/2 reconstitution system (IFNARRS). Proteins to be examined for their interaction are labeled as X and Y. X and Y were fused to the transmembrane and intracellular domains (TM-IN) of IFNAR1 and IFNAR2, respectively. When X and Y bind to each other, IFNAR1-TM-IN and IFNAR2-TM-IN come near and activate TYK2/JAK1 and STAT1/STAT2/IRF9 to induce Nluc activities via ISREs. NlucP is only activated by the specific binding of X and Y via elimination of IFNα-dependent activation with knock-out of the endogenous *IFNAR1* gene. (*B*) An extracellular domain of HER2 and an anti-HER2 affibody were fused to the transmembrane and intracellular domains of IFNAR1 and IFNAR2, as model binding proteins. (*C*) Of the four possible combinations, two ([1] + [4] and [3] + [2]) caused significant increases in Nluc activities when transfected into HEK293T cells with the 6xISRE-NlucP plasmid. After 36 h, Nluc luciferase activities were measured (mean ± SD of three independent experiments). (*D*) Different numbers of ISRE were placed in the promoter of NlucP (*SI Appendix*, Fig. S1*B*) and transfected into HEK293T cells in a 96-well plate (Negative: [3] + [4] and Positive: [3] + [2]). After 36 h, the Nluc assay was performed. Asterisks indicate significant difference compared each Positive to Negative (*P < 0.01, **P < 0.0001). IFNAR1 and IFNAR2, interferon alpha, and beta receptor subunits 1 and 2; EX, extracellular domain; TM, transmembrane region; IN, intracellular domain; JAK, Janus kinase; TYK2, tyrosine kinase 2; STAT, signal transducer and activator of transcription; IRF9, interferon regulatory factor 9; ISRE, interferon-stimulated response element; NlucP, a destabilized form of Nluc with a PEST sequence; PM, plasma membrane; HER2, human EGFR-related 2; and affibody, Af.

**Fig. 2.**
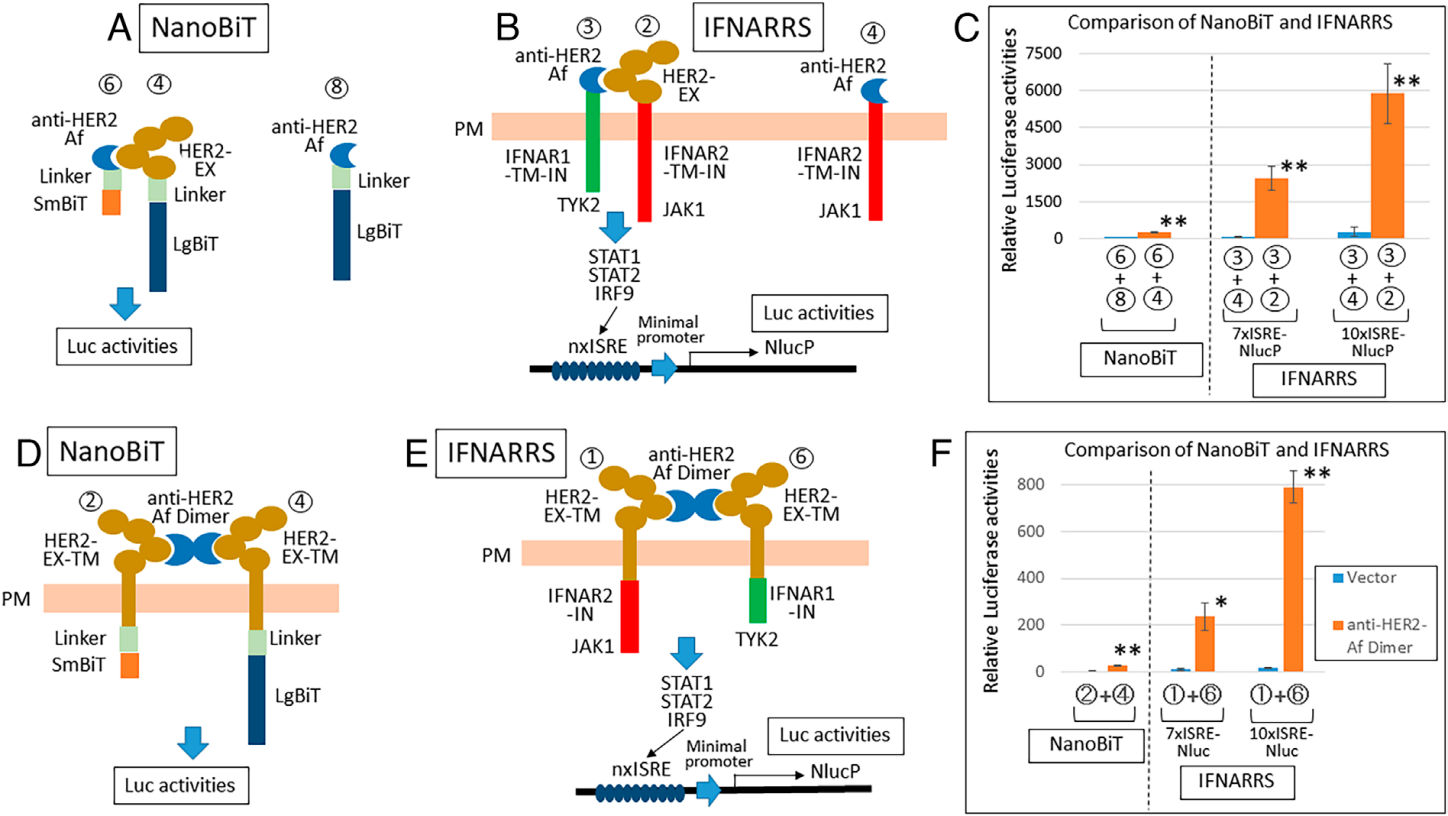
IFNARRS compared with the NanoBiT system. (*A*–*C*) Signaling via the transmembrane and intracellular domains of IFNAR1/2. The same HER2-EX and anti-HER2 affibody in Fig. 1*B* were used in NanoBiT system (*A*) and IFNARRS (*B*). (*A*) The HER2-EX and anti-HER2 affibody were fused to SmBiT and LgBiT, with or without a short peptide linker (Linker) as depicted in *SI Appendix*, Fig. S2 (Negative: [6] + [8] or Positive: [6] + [4]). (*B*) The HER2-EX and anti-HER2 affibody were fused to IFNAR2-TM-IN and IFNAR1-TM-IN depicted in Fig. 1*B* (Negative: [3] + [4] or Positive: [3] + [2]). (*C*) HEK293T cells in a 96-well plate were transfected with the indicated combinations using the same backbone of the plasmid for each gene expression, using 0.2μL Lipofectamin 2000 [(*A*) 15 ng of -SmBiT, 15 ng of -LgBiT, and 10 ng of vector plasmids; (*B*) 15 ng of -IFNAR2, 15 ng of -IFNAR1, 10 ng of Nx ISRE-NlucP plasmids]. After 36 h, the Nluc assay was performed with the same amount of substrate (mean ± SD of three independent experiments). (*D*–*F*) Signaling via the intracellular domains of IFNAR1/2 alone. The same extracellular and transmembrane regions of HER2 (HER2-EX-TM) in *SI Appendix*, Fig. S5*A* were used in NanoBiT system (*D*) and IFNARRS (*E*). (*D*) The HER2-EX-TM was fused to SmBiT and LgBiT with or without Linker, as depicted in *SI Appendix*, Fig. S6. [2] HER2-EX-TM-Linker-SmBiT and [4] HER2-EX-TM-Linker-LgBiT successfully generated the anti-HER2 affibody dimer-dependent signal (Negative: vector or Positive: anti-HER2 Af). (*E*) The HER2-EX-TM was fused to the IFNAR1 and IFNAR2 intracellular domains (IFNAR1-IN and IFNAR2-IN). As shown in *SI Appendix*, Fig. S5, the intracellular [1] IFNAR2-IN-265 and [6] IFNAR1-IN-D62 domains successfully transmitted the signal of the JAK-STAT pathway (Negative: vector or Positive: anti-HER2 Af). (*F*) To compare this IFNARRS to the NanoBiT system for the anti-HER2 affibody dimer-dependent signal, HEK293T cells in a 96-well plate were transfected with the indicated plasmids, using 0.2 μL Lipofectamin 2000 [(*D*) 10 ng of -SmBiT, 10 ng of -LgBiT, and 10 ng of vector plasmids with 10 ng of vector or anti-HER2 Af plasmid; (*E*) 10 ng of -IFNAR2, 10 ng of -IFNAR1, and 10 ng of Nx ISRE-NlucP plasmids with 10 ng of vector or anti-HER2 Af plasmid]. After 36 h, Nluc assay was performed with the same amount of substrate (mean ± SD of three independent experiments). Asterisks indicate significant difference compared each Positive to Negative (*P < 0.01, **P < 0.0001).

To develop a highly sensitive binding assay, we examined whether the EX domains of IFNAR1 and IFNAR2 can be replaced with other proteins of interest without impairing the IN functions of IFNAR1/2 that activate TYK2/JAK1 and STAT1/STAT2/IRF9 for the induction of ISRE-containing ISG expression. As shown in Fig. 1*A*, when two molecules (shown as X and Y) bind, IN IFNAR1/2 comes close and activates the JAK-STAT pathway. The signal is only transduced when X interacts with Y under the condition that the endogenous IFNAR1 is silenced. Herein, we used the EX domain of HER2 and the anti-HER2 affibody as model-binding proteins. These proteins were fused to the TM and IN domains of IFNAR1 and IFNAR2 (Fig. 1*B* and *SI Appendix*, Fig. S1*A*). To confirm that the interaction between HER2-EX and anti-HER2 affibody is linked to IFNAR1/2-mediated signaling ([1], [2], [3], [4]), four possible combinations were cotransfected into HEK293T cells with the 6xISRE-NlucP plasmid. NlucP is a destabilized form of Nluc with a PEST sequence to respond more quickly to changes in transcriptional activity. As expected, two combinations ([1] + [4] and [3] + [2]) induced significant Nluc activities via ISRE (Fig. 1*C*). Of note, the Nluc activities increased with an increasing number of ISRE in the promoter, indicating specific binding of HER2-EX and anti-HER2 affibody (Fig. 1*D* and *SI Appendix*, Fig. S1*B*). To compare this IFNARRS with the NanoBiT system, we fused the same HER2-EX and anti-HER2 affibody to SmBiT and LgBiT with or without a flexible linker peptide (15, 16) (*SI Appendix*, Fig. S2*A*). The reconstituted Nluc activities by the binding of HER2-EX and anti-HER2 affibody increased with the insertion of the flexible linker peptide enclosed in red (*SI Appendix*, Fig. S2 *B*–*E*). Although an exact comparison of IFNARRS and NanoBiT is difficult to perform, we used the same backbone of the plasmid to express each gene, and the same amounts of plasmid DNAs for transfection using Lipofectamine 2000 (Fig. 2 *A*–*C*). The Nluc assay was performed simultaneously with the same amount of substrate. The signal intensities of IFNARRS ([3]+[2]) using 7xISRE and 10xISRE were 10.2- and 24.6-fold higher, respectively, than the strongest combination of NanoBiT ([6]+[4]) with the linker; however, the specificities (signal/noise) were 38.8- and 21.2-fold, respectively, which were lower than the 80.3-fold obtained with the NanoBiT system.

We applied the IFNARRS to various binding assays using the ZZ-affibody (15), which binds to the Fc region of immunoglobulin G instead of the anti-HER2 affibody (ZZ Af-IFNAR1-TM-IN) (*SI Appendix*, Fig. S3). The specific binding of anti-programmed cell death-ligand 1 (PD-L1) antibody to PD-L1 (16) was detected by expressing the EX PD-L1-fused TM and IN domains of IFNAR2 (PD-L1-EX-IFNAR2-TM-IN), ZZ Af-IFNAR1-TM-IN, and 10xISRE-NlucP. Dose-dependent Nluc activities were obtained via the addition of the PD-L1 antibody to transiently transfected cells; however, no effects were obtained with the control anti-GST antibody (*SI Appendix*, Fig. S3*C*). To examine the interaction of PD-L1 to programmed cell death 1 (PD1), a PD1-EX and anti-HER2 affibody fusion protein was fabricated via a short linker peptide (PD1-EX-Linker-anti-HER2 Af) (*SI Appendix*, Fig. S4). The PD1-EX-Linker-anti-HER-2 Af, HER2-EX-IFNAR1-TM-IN, PD-L1-EX-IFNAR2-TM-IN, and 10xISRE-NlucP were transfected into HEK293T cells. The high Nluc activities highlighting the interaction between PD1 and PD-L1 were inhibited in the presence of the anti-PD-L1 antibody in a dose-dependent manner; however, almost no effects were observed with the control anti-GST antibody (*SI Appendix*, Fig. S4*D*). Taken together, the IFNARRS system can be used to examine various types of protein binding.

### Intracellular Domains of IFNAR1/2 Are Sufficient for Signal Transmittance.

The TM regions of some TM proteins play important roles in the correct assembly of each subunit. In T cell receptor (TCR), the eight TM helices of the TCR–CD3 complex (TCRαβ/CD3εγ/CD3εδ/CD3ζζ dimers) form a compact bundle-like structure, mainly via hydrophobic interactions with each other (17). In addition to hydrophobic residues, basic residues in the TM segments of TCRαβ form ionic interactions with acidic residues in the TM segments of CD3εγ/CD3εδ/CD3ζζ, further stabilizing the bundle structure. Therefore, to examine the binding of TM proteins, the native TM regions of these proteins should be included in the assay. We determined whether the IN domains of IFNAR1 and IFNAR2 without TM regions are sufficient to transmit the signal for TYK2/JAK1 and STAT1/STAT2 to induce Nluc activation via ISRE.

The EX and TM regions of HER2 (HER2-EX-TM) were fused to the IFNAR1 and IFNAR2 IN domains (IFNAR1-IN and IFNAR2-IN) (Fig. 2*E* and *SI Appendix*, Fig. S5*A*). As the conjugation of two unrelated domains sometimes impairs the function of each domain, a series of deletions of the IFNAR1-IN domain were constructed. As shown in *SI Appendix*, Fig. S5 *B* and *C*, [1] [HER2-EX-TM-IFNAR2-IN-265 (265-515 amino acid)] and [6] [HER2-EX-TM-IFNAR1-IN-D62 (461 to 537 amino acid)] worked well with anti-HER2-IFNAR1-TM-IN-394 and anti- HER2-IFNAR2-TM-IN-231, respectively, to transmit the JAK- STAT signal (enclosed in red). These results were consistent with the previous report that the IFNAR2-IN (265 to 462 amino acid) and the IFNAR1-IN (463 to 507 amino acid) were sufficient to transmit the IFNα-dependent downstream signal (5). As a result, we transfected [1] and [6] with or without a dimer form of the anti-HER2 affibody into HEK293T cells (Fig. 2*E*). Significant Nluc activities were only induced by the proximity of the IFNAR2-IN-265 and IFNAR1-IN-D62 domains when cotransfected with the anti-HER2 affibody dimer (Fig. 2*F*). Therefore, IN domains of IFNAR1/2 are sufficient to transmit the TYK2/JAK1 and STAT1/STAT2 signals. We also compared this system with NanoBiT by fabricating the same HER2-EX-TM-fused LgBiT and SmBiT with a linker peptide between them (Fig. 2 *D*–*F*), because the reconstituted Nluc activities by the anti-HER2 affibody dimer increased with insertion of the linker peptide (enclosed in red in *SI Appendix*, Fig. S6). The signal intensities of IFNARRS ([1]+[6]) using 7xISRE and 10xISRE were 8.3- and 27.7-fold higher, respectively, than the strongest combination of NanoBiT ([2]+[4]) (Fig. 2*F*). Additionally, the specificities (signal/noise) were 22.0- and 40.9-fold, respectively, which were higher than the 8.4-fold obtained with NanoBiT. These findings affirm the superior sensitivity of IFNARRS over the NanoBiT system.

### Assessment of Ligand-Dependent Clustering of the Receptor Using IFNARRS.

#### Stable HEK293T cell line (GRk-281) for IFNγ detection.

**Fig. 3.**
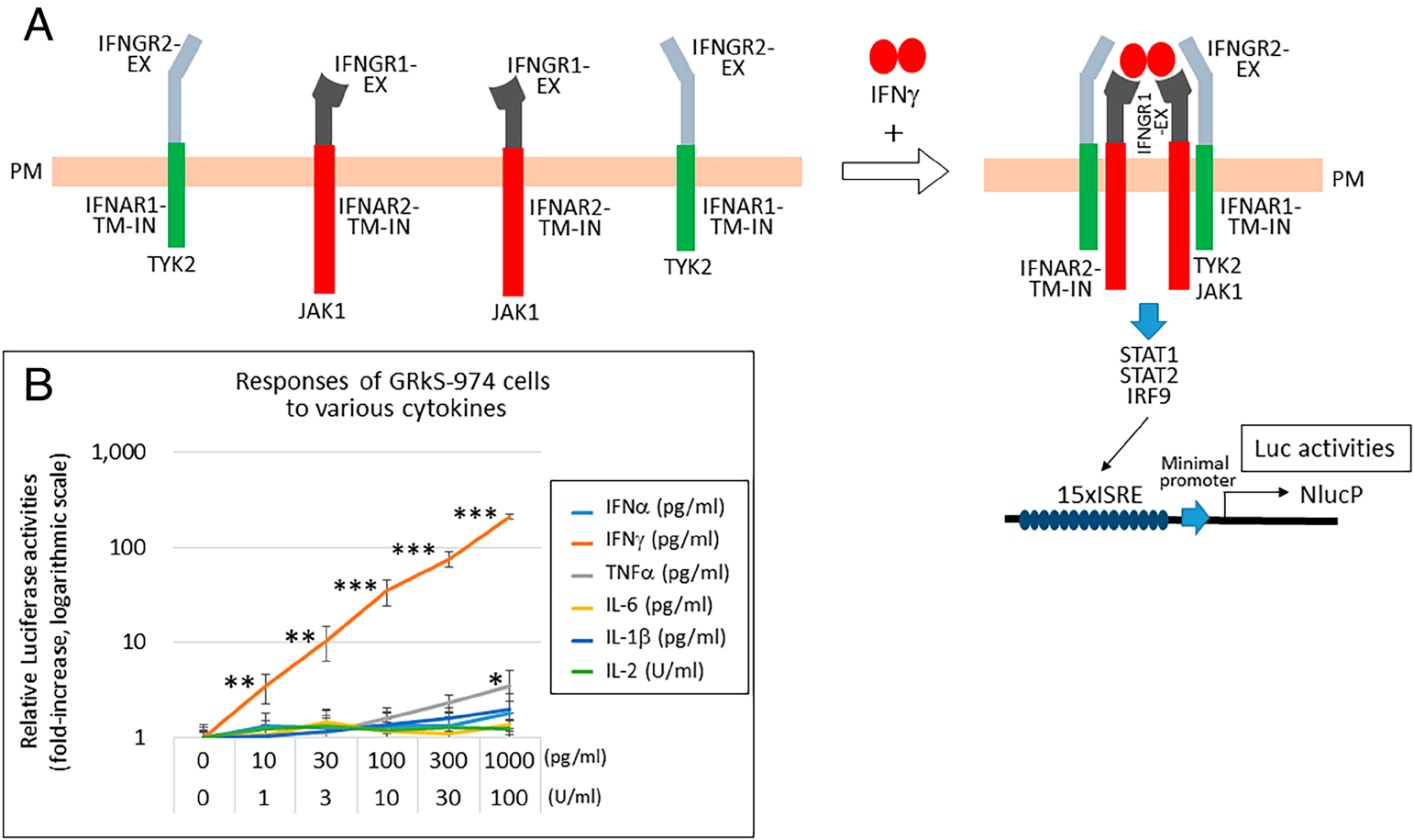
The IFNγ-dependent IFNGR1 and IFNGR2 tetramer formation was detected by the IFNARRS. (*A*) Extracellular domains of IFNGR1 and IFNGR2 (IFNGR1-EX and IFNGR2-EX) were fused to IFNAR2-TM-IN and IFNAR1-TM-IN, respectively. When the dimer form of IFNγ is added, the IFNGR1 and IFNGR2 tetramer is formed, and the intracellular domains of IFNAR1 and IFNAR2 will come into proximity to send the JAK-STAT-dependent signal. (*B*) HEK293T-GRkS-974 cells stably expressing IFNGR1-EX-IFNAR2-TM-IN, IFNGR2-EX-IFNAR1-TM-IN, 15xISRE-NlucP, 7xISRE-IFNγ, and 10xISRE-IL-2 with the endogenous *IFNAR1* gene knocked out were stimulated with indicated concentrations of various cytokines for 12 h. The Nluc assay was then performed and shown, taking the count of medium only as 1 on a logarithmic scale (mean ± SD of three independent experiments). Asterisks indicate significant difference compared with the control (medium only) (*P < 0.01, **P < 0.001, ***P < 0.0001). IFNGR1 and 2, interferon gamma receptor 1 and 2; IFNG, IFN gamma.

The successful detection of protein–protein binding by IFNARRS prompted us to examine ligand-dependent receptor clustering. Notably, activated T cells secreting IFNγ and IL-2 were used to evaluate T cell activities (12–14). If T cell activities can be evaluated with IFNARRS, endogenous T cell functions can be regulated via the ligand-stimulated ISRE-dependent activation of relevant genes, such as cytokines and costimulatory molecules, to affect T cell activation, proliferation, and differentiation, as well as ISRE-dependent reporter Nluc activation (18, 19) (Fig. 9). In contrast to IFNAR1/2 signaling, IFNγ uses the IFNγ receptor composed of two distinct chains, high-affinity IFNGR1 and low-affinity IFNGR2 (10, 11). Binding of dimeric IFNγ to IFNGR1 results in the formation of the IFNGR1 dimer and recruitment of the two IFNGR2 molecules. The assembled receptor tetramer activates the JAK1/JAK2 and STAT1 homodimer-dependent signal. However, the GAS (gamma interferon activation site)-dependent promoter activity is weak (20) compared to the ISRE-dependent promoter (7) (*SI Appendix*, Fig. S7). In the present study, we fused the EX-regions of IFNGR1 and IFNGR2 to the TM and IN regions of IFNAR2 and IFNAR1, respectively (Fig. 3*A*). Based on the signal/noise ratio of IFNγ-dependent Nluc activity, the best combination of the EX, TM, and IN domains was determined by transient expressions of various combinations ([4] and [8] of *SI Appendix*, Fig. S8), and the transfection of 7xISRE-IFNγ enhanced the signal. [4] and [8] were transfected to HEK-293T with 15xISRE-NlucP, 7xISRE-IFNγ, and hygromycin resistance gene-expressing plasmids to obtain stable cell lines. After hygromycin B selection, the stable cell line GR-535, which exhibited the highest signal/noise ratio (IFNγ-stimulated Nluc activities) among several stable cell lines (enclosed in red in *SI Appendix*, Fig. S8*E*), was isolated. To eliminate IFNα-dependent activation in GR-535 cells, the endogenous *IFNAR1* gene was knocked-out via transfection of a Cas9-expressing plasmid and an sgRNA targeting the *IFNAR1* gene with donor dsDNA for homologous recombination. The plasmid was designed to express a puromycin resistance gene after successful recombination (*SI Appendix*, Fig. S9 *A* and *B*). After puromycin selection, the cell line GRk-281, which exhibited the highest signal/noise ratio (IFNγ-stimulated Nluc activities) with no response to IFNα due to the knockout of endogenous *IFNAR1*, was isolated among several stable cell lines (enclosed in red in *SI Appendix*, Fig. S9*C*). The genes expressed or knocked out, and the biological characteristics of stable cell lines obtained here, are listed in Table 1.

**Table 1.** Gene expressions of stable cell lines and their biological characteristics

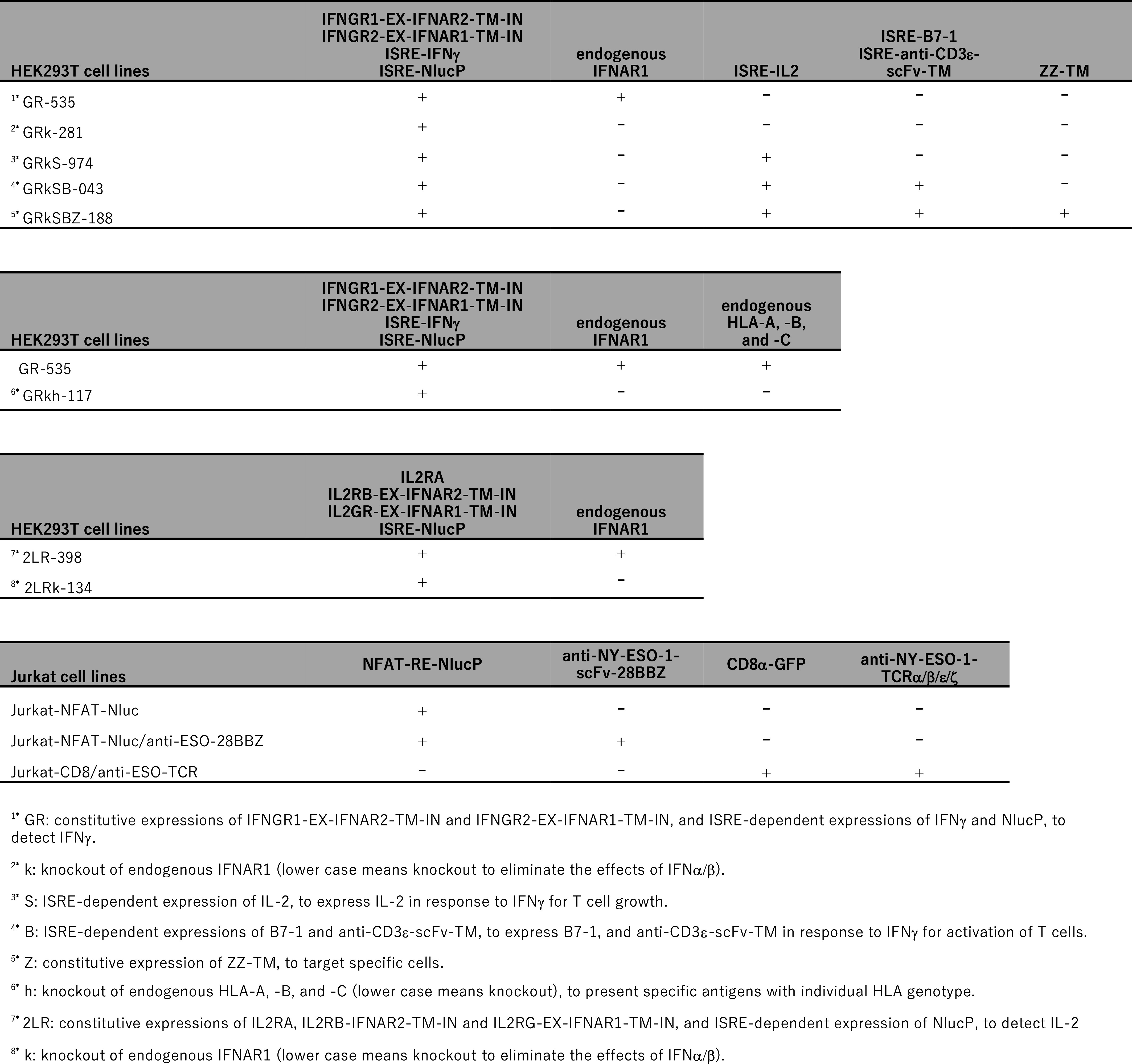

#### Stable HEK293T cell line (2LRk134) for measuring IL-2 levels.

To elucidate the utility of IFNARRS for the analysis of ligand-dependent receptor signaling, we created a chimeric IL-2 receptor with IFNAR1/2. The high-affinity IL-2 receptor consists of IL2Rα/β/γ subunits, and the IL2Rβ/γ subunits have IN signaling domains for activation of the JAK1/JAK3 and STAT5 homodimer signals (12, 13). Extracellular domains of IL2RG and IL2RB (IL2Rγ-EX and IL2Rβ-EX) were fused to IFNAR1-TM-IN and IFNAR2-TM-IN, respectively. Based on the signal/noise ratio of IL-2-dependent Nluc activity, the best combination of [2] + [7] + IL2RA of *SI Appendix*, Fig. S10 were transfected into HEK293T cells with 10xISRE-NlucP and a hygromycin resistance gene-expressing plasmids, to obtain stable cell lines. After hygromycin B selection, the stable cell line, 2LR-398, which exhibited the highest signal/noise ratio (IL-2-dependent Nluc activities), was isolated among several stable cell lines (enclosed in red in *SI Appendix*, Fig. S10*E*). To eliminate IFNα-dependent activation in 2LR-398 cells, the endogenous *IFNAR1* gene was knocked out. The cell line, 2LRk-134, which exhibited the highest IL-2-stimulated Nluc activities, with no response to IFNα via knockout of endogenous *IFNAR1*, was established among several stable cell lines (*SI Appendix*, Fig. S10*F*). 2LRk-134 cells exhibited dose-dependent responses to IL-2 (1 to 100 U/mL), with no responses to other cytokines except a very high concentration of IFNγ (1,000 pg/mL). The luciferase intensity to 1,000 pg/mL IFNγ was comparable to that observed at 1 U/mL IL-2 production. Consequently, 2LRk-134 cells enable the estimation of IL-2 concentrations exceeding 1 U/mL in any given sample.

#### Stable HEK293T cell line (GRkS-974) that secretes IL-2 in response to IFNγ.

**Fig. 4.**
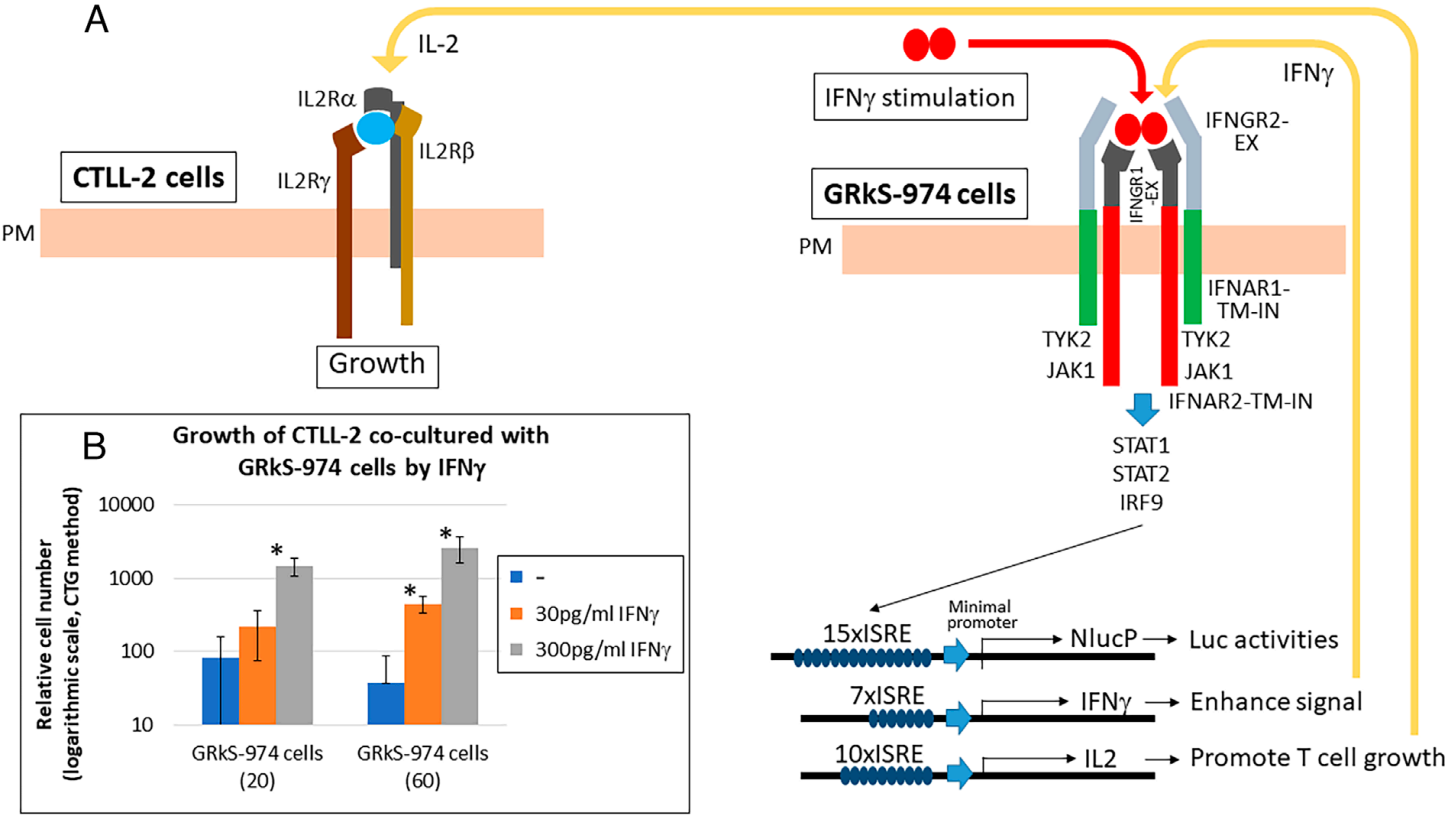
GRkS-974 cells secrete a large amount of IL-2 in response to small amounts of IFNγ. (*A*) Some T cells secrete IFNγ when activated with specific antigens. In response to the secreted IFNγ, GRkS-974 cells stably expressing IFNGR1-EX-IFNAR2-TM-IN, IFNGR2-EX-IFNAR1-TM-IN, 15xISRE-NlucP, 7xISRE-IFNγ, and 10xISRE-IL-2 with the endogenous *IFNAR1* gene knocked out, activate NlucP, IFNγ, and IL-2 genes via ISRE. NlucP shows the activation level. The increased IFNγ enhances the activation signal, and IL-2 promotes the T cell growth. (*B*) CTLL-2 cells (a mouse cell line known to proliferate in response to IL-2) were cocultured with GRkS-974 cells. After the cells (GRkS-974: 20 or 60, CTLL-2: 400) were stimulated with the indicated concentrations of IFNγ or medium only (−) for 72 h, the number of proliferated CTLL-2 cells was assessed by the CellTiter-Glo® assay (CTG) and shown on a logarithmic scale. Asterisks indicate a significant difference from the control (medium only) (*P < 0.01).

To confer IL-2 production in response to IFNγ, GRk-281 cells were further transfected with 10xISRE-IL-2 and a blasticidin resistance gene-expressing plasmid (*SI Appendix*, Fig. S11*A*). The GRkS-974 cell line, showing the highest signal/noise ratio (enclosed in red), secreted a large amount of IL-2 in response to 10 to 1,000 pg/mL IFNγ in dose- and time-dependent manners (*SI Appendix*, Fig. S11 *B* and *C*). The sensitivity for IFNγ is sufficient for detecting some activated T cells (14, 21). Other cytokines except a large amount of TNFα (1,000 pg/mL) did not affect the luciferase activities with significance (*P* < 0.01) (Fig. 3*B*). The luciferase intensity by TNFα was marginal (3.4-fold), compared to the same amount of IFNγ (210-fold), and the sensitivity to give significant response was about 1/100 to that of IFNγ. Therefore, we think only IFNγ is the practical ligand that activates the chimeric receptor. To evaluate further the specificity and sensitivity of IL-2 secreted by GRkS-974 cells with IFNγ, we used mouse CTLL-2 cells that exhibit IL-2-dependent growth (22). When CTLL-2 cells were cocultured with GRkS-974 cells, CTLL-2 cells were found to proliferate significantly with 30 to 300 pg/mL IFNγ in a dose-dependent manner (Fig. 4*B* and *SI Appendix*, Fig. S11*D*). This result indicates that CTLL-2 cells responded to the IL-2 secreted by IFNγ-stimulated GRkS-974 cells as CTLL-2 cells did not grow with IFNγ treatment.

**Fig. 5.**
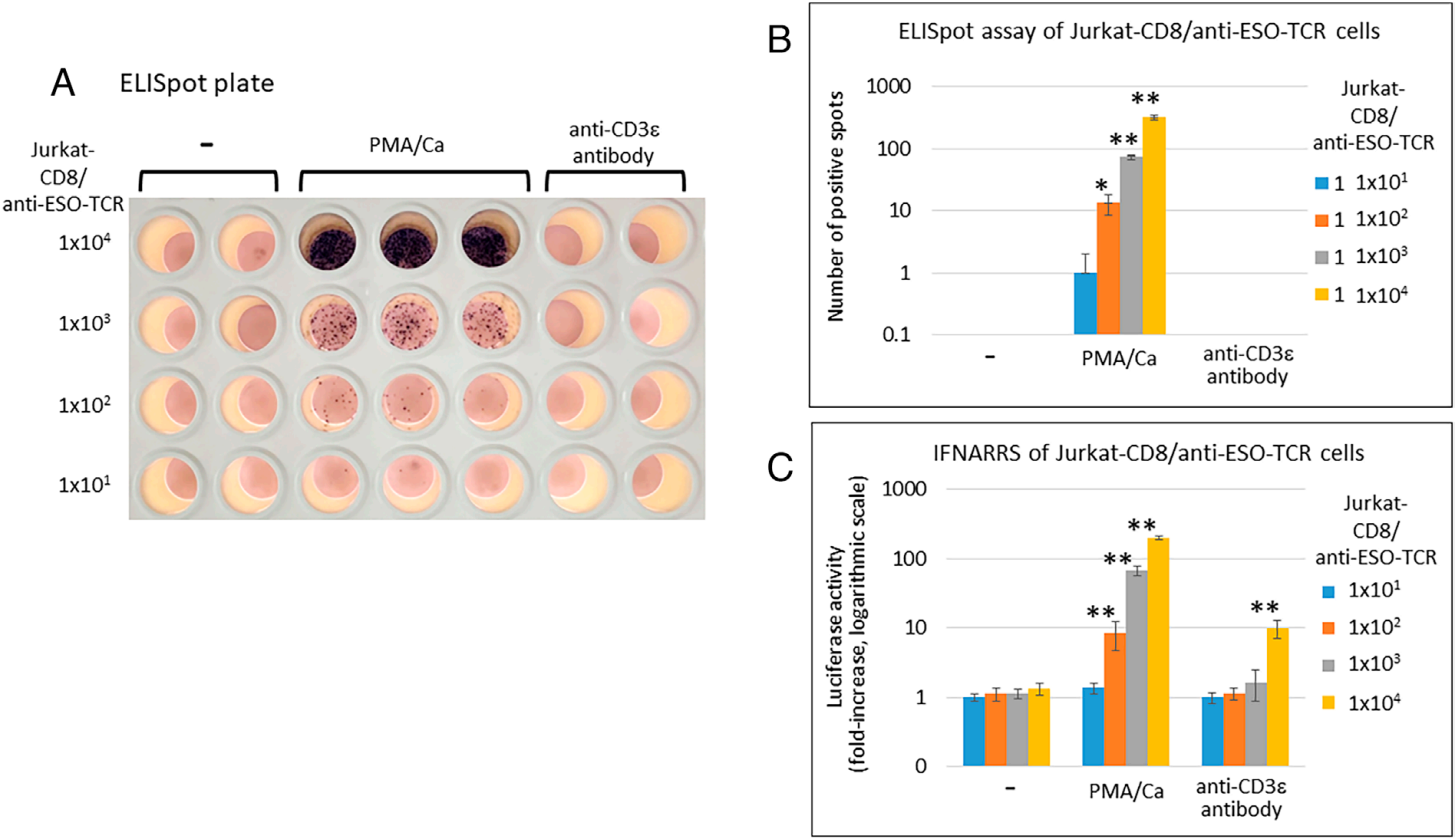
IFNARRS compared with ELISpot assay. (*A*) Indicated numbers of Jurkat-CD8/anti-ESO-TCR cells were stimulated with 100 ng/mL PMA and 300 ng/mL calcium ionophore (PMA/Ca), with 100 ng/mL anti-CD3ε antibody, or medium only (−) for 13 h in a 96-well plate for ELISpot assay to measure the secreted IFN-γ from the T cells. The positive spots are visualized. (*B*) The numbers of positive spots in each well were determined and shown in logarithmic scale (mean ± SD of three wells). (*C*) Simultaneously, the same numbers of Jurkat-CD8/anti-ESO-TCR cells were added to the GRkS-974 cells seeded in a 96-well plate in advance (2 × 10^4^ cells/well). After the same treatments as in *A*, Nluc assay was performed, taking the count of medium only with 1 × 10^1^ Jurkat cells as 1 in the logarithmic scale (mean ± SD of four independent experiments), and shown. Asterisks indicate a significant difference from the control (medium only) (*P < 0.01, **P < 0.0001).

We determined whether GRkS-974 cells can detect the T cells activated via various stimuli. We introduced CD8α-EGFP (23) and a TCRα/β specific to a NY-ESO-1 peptide antigen-loaded MHC class I (14) with CD3ε and CD3ζ (24) into Jurkat T cells via sequential infections with recombinant lentivirus (25) (*SI Appendix*, Fig. S12*A*). Jurkat cells expressing CD8α and anti-NY-ESO-1 TCRα/β/CD3ε/ζ were cocultured with GRkS-974 cells and stimulated with PMA (Phorbol 12-myristate 13-acetate) and calcium ionophore (Ca), or an anti-CD3ε antibody (22). GRkS-974 cells displayed significant activation of Nluc in response to those stimuli (*SI Appendix*, Fig. S12*B*). When GRkS-974 cells were preincubated with the NY-ESO-1 peptide to load endogenous MHC class I, the luciferase activities increased with increasing NY-ESO-1 peptide in a dose-dependent manner via coculture with Jurkat cells expressing CD8α and anti-NY-ESO-1 TCR (*SI Appendix*, Fig. S12*C*). In conventional practice, ELISA and ELISpot assays are commonly used to detect activated T cells. As shown in *SI Appendix*, Fig. S13, this IFNARRS demonstrated higher sensitivity than ELISA in detecting PMA/Ca-activated Jurkat-CD8/anti-ESO-TCR cells. We conducted a comparison between this IFNARRS and ELISpot assays using various numbers of Jurkat-CD8/anti-ESO-TCR cells treated with PMA/Ca or anti-CD3ε antibody (Fig. 5). It is known that the PMA/Ca stimulation elicited a much stronger response compared to the anti-CD3ε antibody stimulation (*SI Appendix*, Fig. S14). While both assays detected 1 × 10^2^ PMA/Ca-activated Jurkat cells, only IFNARRS detected 1 × 10^4^ anti-CD3ε antibody-activated Jurkat cells. In ELISpot assay, strong spots were observed in two out of three wells with 1 × 10^1^ Jurkat cells by PMA/Ca, though not statistically significant. ELISpot can detect even a single T cell with robust stimulation but struggles with weak stimulation such as anti-CD3ε antibody. IFNARRS captures total IFNγ secretion from all cells in a well, successfully detecting 1 × 10^4^ T cells activated by anti-CD3ε antibody, unlike ELISpot. In conclusion, this IFNARRS exhibits sensitivity nearly equivalent to ELISpot and offers an advantage over ELISpot in detecting T cells activated by weaker stimuli.

### Stable Cell Lines GRkSB-043 and GRkSBZ-188 Derived from GRkS-974 Exhibit Functional Dendritic Cell (DC) Activities.

**Fig. 6.**
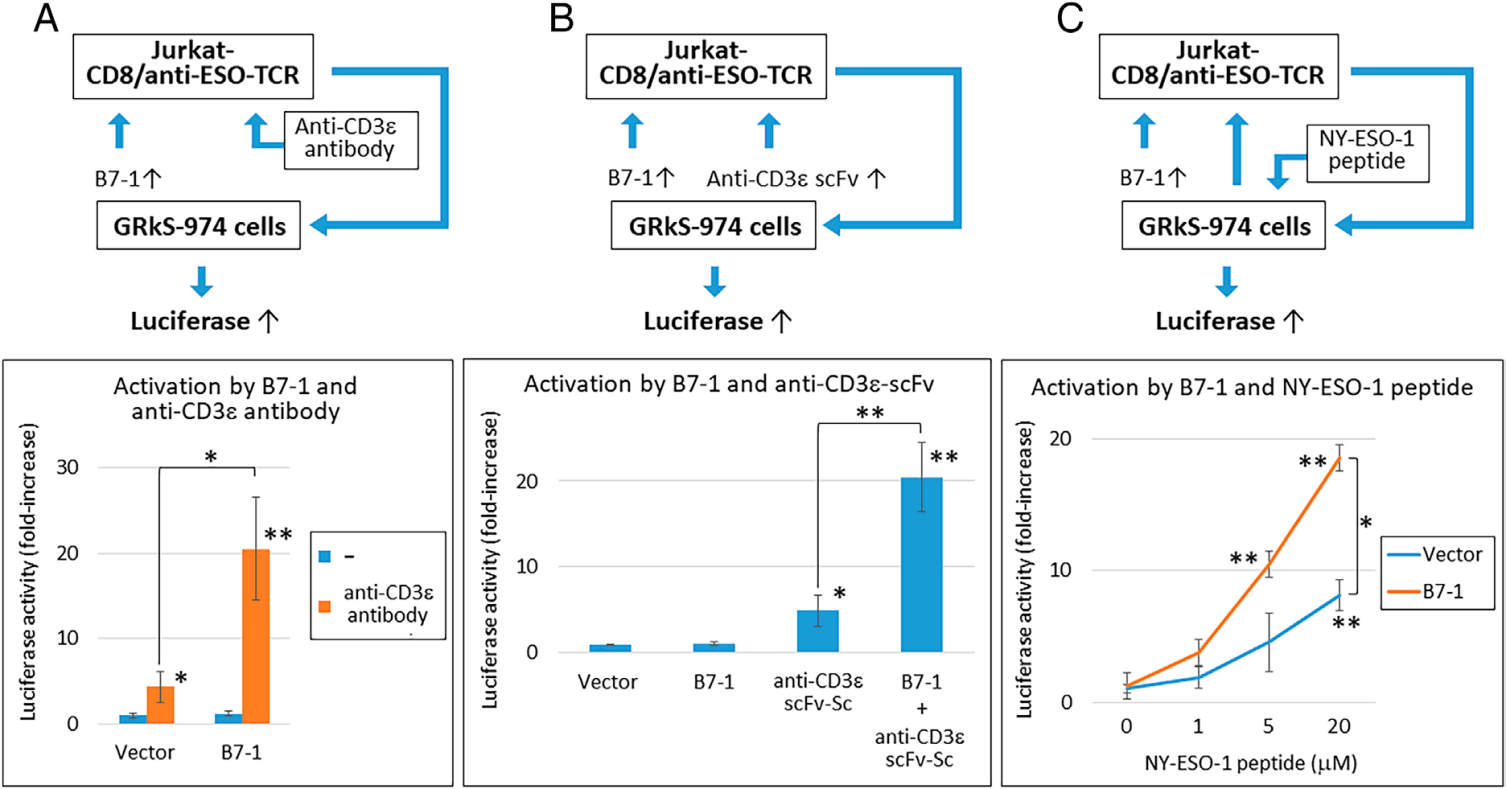
Interactions of GRkS-974 cells and Jurkat cells were enhanced by the anti-CD3ε antibody, B7-1, and antigen peptide stimulation. (*A*) GRkS-974 cells (1 × 10^5^ cells/well) were transfected with a B7-1-expressing vector or empty vector. Jurkat T cells (1 × 10^5^ cells/well) stably expressing CD8α and anti-NY-ESO-1 TCR were added to cells 24 h after transfection and stimulated with 100 ng/mL anti-CD3ε antibody for 12 h. Nluc assay was performed by taking the empty vector transfection without anti-CD3ε antibody stimulation as 1 (mean ± SD of three independent experiments). (*B*) As indicated, GRkS-974 cells were transfected with vectors expressing B7-1 or secretory anti-CD3ε scFv-Sc. Jurkat cells stably expressing CD8α and anti-NY-ESO-1 TCR were added to the cells 24 h after transfection. After 12 h of coculture, Nluc assay was performed by taking the empty vector transfection as 1 (mean ± SD of three independent experiments). (*C*) GRkS-974 cells were transfected with the B7-1-expressing or empty vector. The cells were further incubated for 12 h with the indicated concentrations of NY-ESO-1 peptide to load on the surface MHC class I 24 h after transfection. After that, Jurkat cells stably expressing CD8α and anti-NY-ESO-1 TCR were added to the cells. After 12 h of coculture, Nluc assay was performed, taking the vector transfection without NY-ESO-1 peptide as 1 (mean ± SD of three independent experiments). Asterisks indicate a significant difference from the vector transfection without stimulation or between the indicated pairs (*P < 0.01, **P < 0.001). TCR, T cell receptor; and scFv-Sc, secretory single-chain variable fragment.

Some T cells, such as cytotoxic T and Th1 cells, secrete IFNγ and IL-2 when activated (14, 21). The stable GRkS-974 cells secrete a large amount of IL-2 in response to 10 to 1,000 pg/mL IFNγ in a dose-dependent manner. Several costimulatory molecules were reported to activate and induce the proliferation of naive T cells via DCs (26). We examined the effects of costimulators, such as B7-1, CD40, and 4-1BB-L, on Jurkat cells expressing the TCR specific for the NY-ESO-1 peptide activated nonspecifically by an anti-CD3ε antibody or specifically by NY-ESO-1 peptide-loaded MHC class I. Jurkat cells were stimulated with an anti-CD3ε antibody. B7-1 significantly enhanced the anti-CD3ε antibody- or secretory anti-CD3ε-single chain variable fragment (scFv-Sc)-dependent activation (Fig. 6 *A* and *B*) (27). B7-1 expression also enhanced NY-ESO-1 peptide-dependent activation (Fig. 6*C*). Consequently, we constructed plasmids to express B7-1, a secretory anti-CD3ε scFv (anti-CD3ε-scFv-Sc), and a membrane-anchored anti-CD3ε scFv (anti-CD3ε-scFv-TM) under the 10xISRE-dependent promoter (*SI Appendix*, Fig. S15). The IFNγ-dependent activation of GRkS-974 cells with Jurkat cells was significantly enhanced by the coexpression of B-7 and anti-CD3ε-scFv-TM via ISRE.

**Fig. 7.**
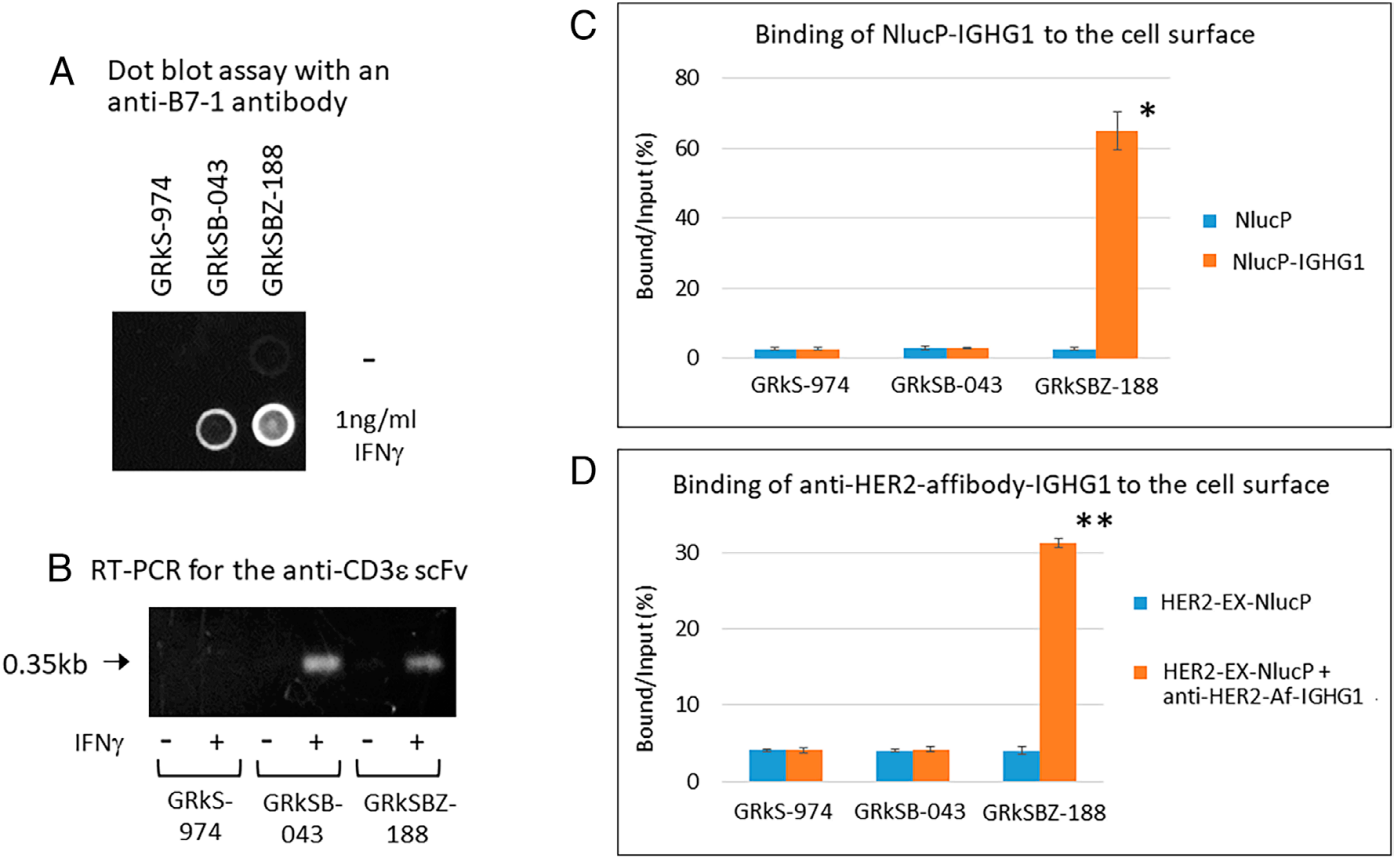
Expressions of B7-1, anti-CD3ε-scFv-TM, and ZZ-TM in the GRkS-974, GRkSB-043, and GRkSBZ-188 cells. (*A*) To confirm IFNγ-dependent expression of B7-1 in GRkSB-043 and GRkSBZ-188 cells, the GRkS-974, GRkSB-043, and GRkSBZ-188 cells (1 × 10^5^, each) on a 24-well plate were treated with 1ng/mL IFNγ for 24 h. The cell lysate (5 μg protein of each) was dotted onto a nitrocellulose membrane. The B7-1 proteins were visualized with an anti-B7-1 antibody, HRP-conjugated anti-mouse IgG antibody, and ECL reagent. (*B*). GRkS-974, GRkSB-043, and GRkSBZ-188 cells (1 × 10^5^ each) on a 24-well plate were treated with 1ng/mL IFNγ for 24 h. The 1 μg of total RNA was subjected to reverse transcription and used in PCR using the specific primers (for anti-CD3ε-scFv-TM). The products were electrophoresed in a 1% agarose gel and stained with ethidium bromide. The arrow indicates the expected PCR product (0.35 kb). (*C*) and (*D*) show specific bindings of NlucP-IGHG1 and anti-HER2-affibody-IGHG1 to the surface ZZ-TM on GRkSBZ-188 cells. In *C*, plasmids designed to secrete NlucP or NlucP-fused IGHG1 proteins were transfected to HEK293T cells, the supernatants were collected, and one aliquot was subjected to binding assay. The same counts (25,000) of NlucP or NlucP-IGHG1 were incubated with GRkS-974, GRkSB-043, and GRkSBZ-188 cells for 12 h. After removing the supernatant, the Nluc activities bound to cells were measured and shown as Bound/Input. Compared to NlucP, NlucP-IGHG1 was bound specifically to GRkSBZ-188 cells. In *D*, plasmids designed to secrete HER2-EX-NlucP or anti-HER2-affibody-IGHG1 proteins were transfected to HEK293T cells (1 × 10^5^ each), the supernatants were collected, and one aliquot was subjected to binding assay. The same counts (15,000) of HER2-EX-NlucP with or without anti-HER2-affibody-IGHG1 were incubated with GRkS-974, GRkSB-043, and GRkSBZ-188 cells for 12 h. After removing the supernatant, the Nluc activities bound to cells were measured and shown as Bound/Input. Compared to HER2-EX-NlucP only, HER2-EX-NlucP with anti-HER2-affibody-IGHG1 specifically bound to GRkSBZ-188 cells. Asterisks indicate a significant difference from the control (*C*, NlucP or *D*, HER2-EX-NlucP only) (*P < 0.0001, **P < 0.000001).

**Fig. 8.**
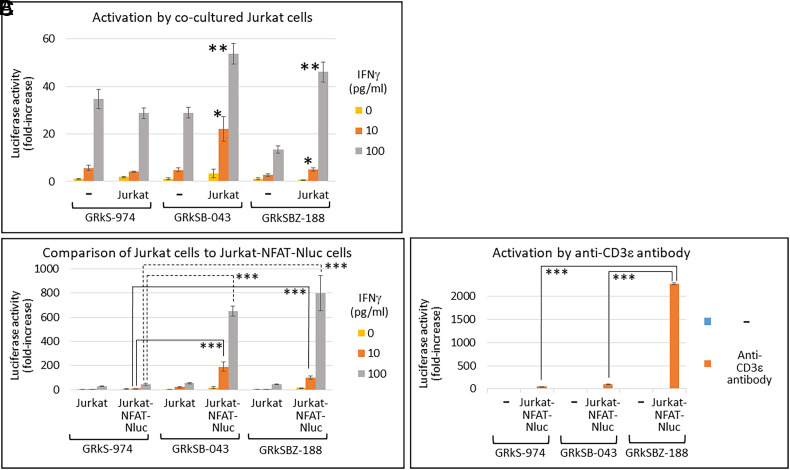
Characterization of GRkSB-043 and GRkSBZ-188 cells. (*A*) GRkS-974, GRkSB-043, and GRkSBZ-188 cells (2 × 10^4^ each) were cocultured with Jurkat cells (3 × 10^4^ each). In contrast to parent GRkS-974 cells, the GRkSB-043 and GRkSBZ-188 cells stably expressing B7-1 and anti-CD3ε-scFv-TM were significantly activated by the cocultured Jurkat cells in response to IFNγ. Nluc assay was performed, and the count without IFNγ and Jurkat cells was shown as 1 (mean ± SD of three independent experiments). Asterisks indicate significant differences between with or without Jurkat cells in the same concentration of IFNγ (*P < 0.01, **P < 0.001). (*B*) To evaluate the Jurkat cells’ activation, the same numbers of Jurkat-NFAT-Nluc cells (3 × 10^4^ each) were used instead of Jurkat cells. The difference reflects the Jurkat-NFAT-Nluc activation. (*C*) Compared to GRkS-974 and GRkSB-043 cells, GRkSBZ-188 cells expressing stably ZZ-TM significantly enhanced the anti-CD3ε antibody-dependent activation of cocultured Jurkat-NFAT-Nluc cells by recruiting the anti-CD3ε antibody on the cell surface via ZZ-TM. Nluc assay was performed, taking the count without IFNγ and Jurkat cells as 1 (mean ± SD of three independent experiments). Asterisks indicate significant differences between the indicated pairs (***P < 0.0001).

**Fig. 9.**
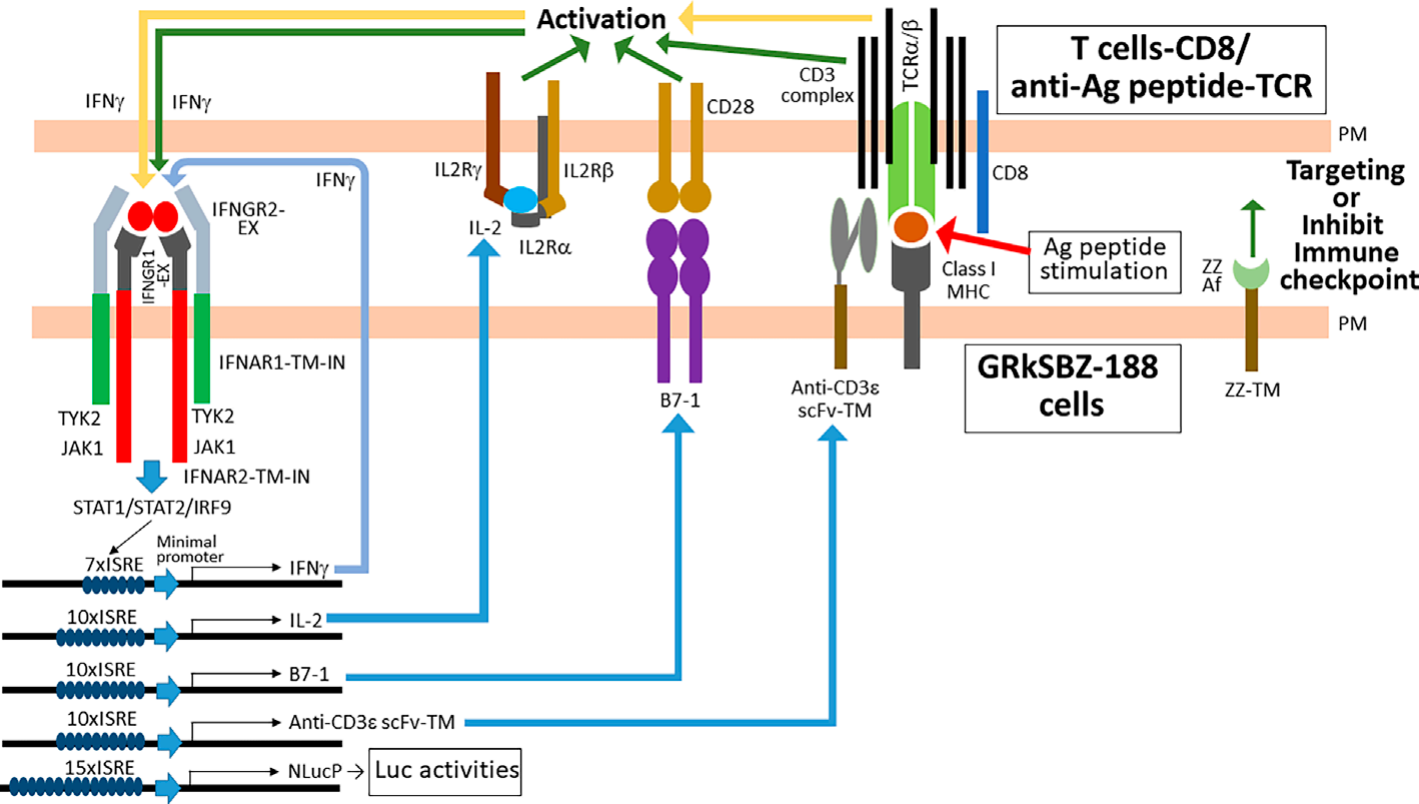
GRkSBZ-188 cells emulate some DC functions. GRkSBZ-188 cells stably express IL-2, B7-1, and anti-CD3ε-scFv-TM in response to IFNγ, and express constitutively ZZ-TM to recruit on the cell surface any antibodies via the Fc-region. IL-2 is very important for the activation and proliferation of endogenous T cells, and simultaneous stimulation of TCR by specific antigens and costimulatory molecules such as CD28 is essential for proper activation and proliferation of naïve T cells in vivo. In addition, immune checkpoint inhibitors, including anti-PD-1 antibodies, prevent T cell exhaustion. GRkSBZ-188 cells exhibit all those functions in response to IFNγ, by just coculturing with endogenous T cells.

To validate the findings from the transient expressions mentioned above and ensure the effects of B7-1 and anti-CD3ε scFv-TM by just coculturing, we established two stable cell lines (GRkSB-043 and GRkSBZ-188) by expressing B7-1 and anti-CD3ε scFv-TM in response to IFNγ. GRkSB-043 and GRkSBZ-188 cells expressed B7-1 and anti-CD3ε-scFv-TM in response to IFNγ (Fig. 7 *A* and *B*). Additionally, in GRkSBZ-188 cells, apart from B7-1 and anti-CD3ε scFv-TM, functional ZZ affibody was expressed on the cell surface to bind to the Fc-regions of IGHG1 (immunoglobulin heavy constant gamma 1) in NlucP-IGHG1 and anti-HER2-affibody-IGHG1 (28) (*SI Appendix*, Fig. S16 and Fig. 7 *C* and *D*). The IFNγ treatments increased the Nluc activities in all GRkS-974, GRkSB-043, and GRkSBZ-188 cells by activating the chimeric receptors on the cell surface (Fig. 8*A*). Consistent with the transient experiments (*SI Appendix*, Fig. S15*B*) when cocultured with Jurkat cells, treatment with IFNγ increased Nluc activities in GRkSB-043 and GRkSBZ-188 cells but not in GRkS-974. This effect was attributed to the activation of Jurkat cells by the expressions of B7-1 and anti-CD3ε-scFv-TM in GRkSB-043 and GRkSBZ-188 cells but not in GRkS-974 cells. To examine TCR activations in T cells, NFAT-RE (Nuclear Factor of Activated T cells-response element) is commonly utilized by employing an NlucP gene as a reporter (29). We employed Jurkat cells stably expressing NlucP under the control of 6xNFAT-RE (Jurkat-NFAT-NlucP) to assess the effects of Jurkat cells by the expressions of B7-1 and anti-CD3ε-scFv-TM in HEK293T cells (*SI Appendix*, Fig. S14). When cocultured with Jurkat-NFAT-Nluc instead of Jurkat cells, the GRkSB-043 and GRkSBZ-188 cells showed further enhanced Nluc activities, whereas GRkS-974 cells did not exhibit such enhancement (Fig. 8*B*). These differences between Jurkat and Jurkat-NFAT-Nluc reflected the Nluc activities from Jurkat-NFAT-Nluc and clearly indicated the B7-1- and anti-CD3ε-scFv-TM-dependent activations of Jurkat-NFAT-Nluc cells. In addition, the GRkSBZ-188 cells expressing ZZ-TM enhanced the T cell activation by recruiting the anti-CD3ε antibody to the cell surface (Fig. 8*C*). By utilizing specific antibodies to recognize target cells, the GRkSBZ-188 cells exhibited enhanced activation of the target cells compared to other cells (Fig. 9).

Finally, we have established a stable cell line by knocking out endogenous HLA-A, HLA-B, and HLA-C (30) as well as endogenous IFNAR1 of GR-535 cells, to present specific antigens with the individual HLA genotype of interest by re-expression (Table 1). As shown in *SI Appendix*, Fig. S17, the knockout GRkh-117 cells showed no response to transfected NY-ESO-1 cDNA or NY-OSO-1 peptide loading due to the lack of endogenous HLAs against Jurkat cells expressing NFAT-RE-Nluc and anti-NY-ESO-1-scFv-28BBZ (31, 32) (*SI Appendix*, Fig. S14), in contrast to GRk-281 cells. However, the responses to the peptide–HLA-A2 complex were restored simultaneously upon transfection with an HLA-A2 gene. Both GRk-281 and GRkh-117 cells lack IFNα-responses because of endogenous IFNAR1 knockout.

## Discussion

The IFNARRS proves to be a highly sensitive method for detecting protein interactions, making it particularly useful for detecting even in the weakest protein–protein interactions. Notably, this system offers significant potential for analyzing and harnessing various ligand-dependent receptor clusters. The stable GRkS-974 cell line expressing IFNGR1-EX-IFNAR2-TM-IN, IFNGR2-EX-IFNAR1-TM-IN, 15xISRE-NlucP, 7xISRE-IFNγ, and 10xISRE-IL-2 with the endogenous *IFNAR1* gene knocked out exhibits robust IL-2 secretion in response to minimal IFNγ stimulation. Furthermore, these cells can detect Jurkat cells activated nonspecifically with PMA and calcium ionophore, specifically with the NY-ESO-1 peptide-loaded MHC class I. The ISRE-dependent expression of costimulator B7-1 and anti-CD3ε-scFv-TM demonstrates a synergistic activation of Jurkat cells. When clonally expanded T cells are obtained from clinical samples, TCR repertoire analysis can determine the TCR structures (33). The possible candidates include activated T cells targeting neoantigens in cancer or activated T cells with unidentified antigens in certain autoimmune diseases. While sophisticated methods exist for identifying cognate TCR structures against cancer neoantigens, many of these techniques are complex and computationally intensive, often relying on dendritic cells and extensive computer analysis (21, 34, 35). In contrast, the GRkSBZ-188 cells have an advantage over the traditional methods of providing costimulation via agonistic antibody (e.g., anti-CD28) + anti-CD3ε in solution. When antigen-specific T cells are activated by the peptide-MHC on the GRkSBZ-188 cells, the GRkSBZ-188 cells start expressing B7-1, anti-CD3ε scFv-TM in response to the secreted IFNγ from the activated T cells and further stimulate and proliferate the antigen-specific T cells selectively around the GRkSBZ-188 cells, because the B7-1, anti-CD3ε scFv-TM are on the cell surface of GRkSBZ-188 cells. In addition, recruiting immune checkpoint inhibitors like anti-PD1 antibodies on the GRkSBZ-188 cell surface could effectively and selectively prevent exhaustion of the activated T cells than the traditional use of anti-PD1 antibodies in solution (36), as shown in Fig. 9. In this assay, however, there is the caveat of nonspecific T cell expansion post cognate epitope:MHC-I/TCR engagement. The ability of GRkSBZ-188 cells to expand the antigen-specific T cells meaningfully over background will require future experimental validation. Once validated, this simple and cost-effective method can be valuable for analyzing activated T cells from numerous patient cohorts, potentially revealing distinct patterns of activated TCRs associated with beneficial effects.

Indeed, the IFNARRS holds promise for analyzing a wide array of biological phenomena beyond cytokine signaling. Another advantage of IFNARRS is that it assesses interactions between transmembrane proteins (Fig. 2 *D*–*F*). As shown in Fig. 3, in response to IFNγ, IFNGRs form a heterotetramer in the presence of both IFNGR1 and IFNGR2. However, IFNGR1 forms a homodimer in the absence of IFNGR2. The IFNγ-induced dimer formation of IFNGR1 containing the transmembrane (TM) region was only detected by IFNARRS but not by the NanoBiT system (*SI Appendix*, Figs. S18 and S19). In immune checkpoint therapy, where molecules are clustered in central and peripheral immune synapses, this system could examine the intricate interactions between various immune checkpoint molecules, including those involving the transmembrane region (37). Understanding these interactions could lead to more effective combined immune checkpoint therapies. Moreover, recent research has revealed that many G protein-coupled receptors (GPCRs) modulate their signaling by forming homo- or hetero-oligomers in addition to their conventional coupling to specific G proteins (38, 39). Given the involvement of GPCRs in diverse biological events and the development of numerous targeted drugs, the IFNARRS provides a precise tool for analyzing interactions between these transmembrane proteins. This capability could advance our understanding of GPCR signaling and facilitate the development of therapeutics targeting these receptors.

In therapeutic applications, cognate DCs are ideal for activating naive T cells or proliferating activated T cells in response to pathogens, cancer neoantigens, and other stimuli. However, generating large quantities of DCs is challenging. Artificial materials mimicking DCs have recently been explored for ex vivo activation and proliferation of T cells (40, 41). If HEK293T cells can be separated from T cells based on differences in size, adherence, and anchorage dependency (42, 43), GRkSBZ-188 cells could serve as an alternative method to activate and proliferate T cells in response to specific antigens ex vivo (*SI Appendix*, Fig. S20). This approach can potentially simplify and enhance ex vivo T cell regulation for therapeutic purposes. By leveraging the IFNARRS to design HEK293T cells, target T cells appropriately could be functionally modified by activating desired genes via ISRE without the need for genetic engineering of the T cells themselves.

## Materials and Methods

### Cell Culture and Reagents.

Human embryonic kidney HEK293T cells were cultured in Dulbecco’s modified Eagle’s medium supplemented with 10% fetal bovine serum. Jurkat T cells were cultured in RPMI 1640 medium supplemented with 10% fetal bovine serum. CTLL-2 cells (22) were cultured in RPMI 1640 medium supplemented with 10% fetal bovine serum and 50 μM 2-mercaptoethanol. Recombinant human IFNγ (FUJIFILM Wako Chemicals, #CYT-206), IL-2 (KYOWA Pharmaceutical Industry, # 6399), phorbol 12-myristate 13-acetate (PMA, Sigma-Aldrich, #P1585), calcium ionophore (Sigma-Aldrich, #C7522), and anti-GST (Santa Cruz Biotechnology, Inc, #sc-138) and anti-B7-1 (Proteintech #65083-1-Ig) antibodies were used in this study. The anti-PD-L1 (27A2) and anti-CD3ε (OKT3) antibodies are described elsewhere (16, 22).

### cDNA Cloning and Vector Constructs.

Total mRNA and cDNA were obtained, as described previously (25). The cDNAs from HEK293T cells were used as a template to amplify the *IFNAR1*, *IFNAR2*, *IFNGR1*, and *IFNGR2* genes with PrimeSTAR Max DNA Polymerase (Takara Bio Inc). The DNA primers used to amplify the respective genes are listed in *SI Appendix*, Table S1. The cDNAs from Jurkat cells were used to amplify IFNG, IL-2, IL2RA, IL2RB, and IL2RG. The cDNA from human B cells was used to amplify the B7-1 genes. Double-strand DNAs for SmBiT, LgBiT, PD-L1, and PD-1, the α and β chains of TCR for the anti-NY-ESO-1 (14), and anti-CD3ε scFv gene (27) were purchased from IDT Inc. The NlucP (a destabilized form of Nluc with a PEST sequence) expression vector with a minimal promoter (NlucP/MinP) was purchased from Promega Co. The expression vectors for HER2 (#16257), CD8α-EGFP (#86051), TCRα/β/CD3ε/ζ (#89347), Cas9 (#52961), pSLCAR-CD19-28z (#135991), and pSLCAR-CD19-BBz (#135992) were obtained from Addgene. RIKEN BRC provided the IHGH1 (IRAK174M03). The GAS-Luc and anti-NY-ESO-1-scFv (3M4E5) were described in refs. 20 and 31, respectively.

The DNA sequences in *SI Appendix*, Figs. S1–S19 are shown in *SI Appendix*, Tables S2–S14. All constructs were confirmed via sequencing.

### Establishment of Stable Clones.

To establish stable HEK293T cells for IFNγ detection, we cotransfected five pcDNA expression vectors, each containing IFNGR1-EX-IFNAR2-TM-IN, IFNGR2-EX-IFNAR1-TM-IN, 15xISRE-NlucP, 7xISRE-IFNγ, and a hygromycin resistance gene under a CMV promoter (total DNA: 400 ng, 10:10:10:10:1, 1.2 μL Lipofectamine 2000 from Thermo Fisher Scientific Inc). After selection with 0.5 mg/mL hygromycin B for 2 wk, a stable cell line, GR-535, which exhibited the highest signal/background ratio (IFNγ-stimulated Nluc activities), was isolated among several stable clones. To eliminate IFNα-dependent activation in GR-535 cells, the endogenous *IFNAR1* gene was knocked-out by cotransfecting a Cas9-expressing plasmid (44) and a sgRNA-expressing plasmid with a donor dsDNA for homologous recombination (total DNA: 300 ng, 1:2, 1.2 μL Lipofectamine 2000). The plasmid was designed to ensure the expression of the puromycin resistance gene after successful recombination. After selection with 2.8 μg/mL puromycin for two weeks, the cell line GRk-281, which exhibited the highest signal/background ratio (IFNγ-stimulated Nluc activities) and no response to IFNα due to knockout of endogenous *IFNAR1*, was isolated among several stable clones. To confer IL-2 production in response to IFNγ, the GRk-281 cells were further cotransfected with 10xISRE-IL-2- and a blasticidin resistance gene-expressing plasmid (total DNA: 300 ng, 60:1, 1.2 μL Lipofectamine 2000). After selection with 64 μg/mL blasticidin S for 3 wk, the cell line, GRkS-974, which exhibited the highest signal/background ratio (IFNγ-stimulated IL-2 production), was established among several stable clones. The GRkS-974 cells were further cotransfected with 10xISRE-B7-1-, 10xISRE-anti-CD3ε-scFv-TM-, and a zeocin resistance gene-expressing plasmid (total DNA: 400 ng, 1.5:1.5:1, 1.2 μL Lipofectamine 2000). After three weeks of selection with 125 µL/mL zeocin, the cell line GRkSB-043, which expresses B7-1 and anti-CD3ε antibody in response to IFNγ, was established. The GRkS-974 cells were also cotransfected with 10xISRE-B7-1-, 10xISRE-anti-CD3ε-scFv-TM-, ZZ-TM-, and a zeocin resistance gene-expressing plasmid (total DNA: 400 ng, 1:1:1:1, 1.2 μL Lipofectamine 2000). After selection with 125 μg/mL zeocin for 3 wk, the cell line GRkSBZ-188, which expresses B7-1 and anti-CD3ε antibody in response to IFNγ and constitutively expressed ZZ-TM, was established.

To establish stable HEK293T cells for IL-2 detection, we cotransfected five pcDNA expression vectors, each containing IL2Rα, IL2Rβ-EX-IFNAR2-TM-IN, IL2Rγ-EX-IFNAR1-TM-IN, 10xISRE-NlucP, and a hygromycin resistance gene. After hygromycin B selection, the stable cell line, 2LR-398, which exhibited the highest signal/background ratio (IL-2-stimulated Nluc activities) was isolated among several stable clones. The endogenous *IFNAR1* gene of 2LR-398 cells was knocked out using the same method mentioned above. The cell line, 2LRk-134, which exhibited the highest signal/noise ratio (IL-2-stimulated Nluc activities) and no response to IFNα due to knockout of endogenous *IFNAR1*, was established. We confirmed that all the established clones above showed their specified characteristics over several weeks. In addition, each clone was subjected to subcloning by seeding 10 and 100 in a 6-well plate, and the distinct isolated several subclones showed the same characteristics as the parent cells.

Jurkat cells were infected with RetroNectin (Takara Bio Inc., #T100A) and a recombinant lentivirus to express CD8α-EGFP (23) and a puromycin resistance gene, as described previously (25). After selection with 1.3 μg/mL puromycin, fluorescent CD8α-EGFP-expressing cells were isolated. Jurkat cells expressing CD8α-EGFP were further infected with a recombinant lentivirus to express anti-NY-ESO-1 TCRα/β, CD3ε/ζ, and a blasticidin resistance gene. The variable regions of TCRα/β in pLentivirus for TCRα/β/CD3ε/ζ expression (24) were replaced with an anti-NY-ESO-1 TCRα/β (14). After selection with 20 μg/mL blasticidin S, the expression of anti-NY-ESO-1 TCRα/β was confirmed by IFNγ production in response to the NY-ESO-1 peptide-loaded HEK293T cells. Jurkat cells expressing CD8α-EGFP and anti-NY-ESO-1 TCRα/β with CD3ε/ζ were used for the experiments. To assess TCR activation, Jurkat cells were infected with a recombinant lentivirus to express NlucP under control of 6xNFAT-RE (GGAGGAAAAACTGTTTCATACAGAAGGCGT) (29) and a constitutive zeocin resistance gene. After selection with 400 μg/mL zeocin, Jurkat-NFAT-RE-NlucP cells that showed PMA/Ca (100 ng/mL PMA and 300 ng/mL calcium ionophore)-dependent Nluc activation were established. The anti-NY-ESO-1-scFv (31) was fused to 28BBZ of CAR-T construct (32) to express anti-NY-ESO-1-scFv-28BBZ. A recombinant lentivirus was used to express the anti-NY-ESO-1-scFv-28BBZ, and a blasticidin resistance gene was infected in the Jurkat-NFAT-Nluc cells. After selection with 20 μg/mL blasticidin S, Jurkat-NFAT-RE-NlucP/anti-NY-ESO-1-scFv-28BBZ cells that showed Nluc activation to NY-ESO-1 peptide-loaded HEK292T cells were established.

### Peptide Loading.

Highly purified NY-ESO-1 peptide (SLLMWITQC) was purchased from GenScript Biotech. HEK293T cells were loaded onto the endogenous major histocompatibility complex (MHC) Class I HLA-A molecule of HEK293T cells via incubation with 1 to 20 μM NY-ESO-1 peptide for 12 h.

### Transient Transfection and Nluc Assay.

HEK293T cells (2 × 10^4^/well) were seeded in 96-well plates and transfected 12 h later with the indicated plasmid DNAs (total amount of 40 ng) using 0.2 μL Lipofectamine 2000. At 24 h posttransfection, the cells were stimulated with the indicated reagents for 12 h, unless otherwise stated. The luciferase activity was determined by adding the substrate of Nano-Glo® Luciferase Assay System (Promega # N1110).

Of note, some stable cell lines were only treated with the indicated reagents, and the luciferase activity was determined.

### IL-2 Production Assay.

To measure IL-2 concentration in the medium, 10 μL medium was added to 40 μL of 2LRk-134 cells seeded in a round bottom 96-well plate 12 h before the assay. Calibration was performed with 10 μL of a series of purified IL-2 (0, 3, 10, 30, 100 U/mL) instead of medium. Nluc luciferase assay was performed 12 h after the addition of the medium. The IL-2 concentration of the medium was calculated using a third-degree polynomial equation.

IL-2 production was assessed using CTLL-2 cells, which proliferate in response to IL-2. HEK293T-GRkS-974 cells (20 to 60 in 20 μL) seeded in a round bottom 96-well plate and CTLL-2 cells (200 to 400 in 40 μL) were added to GRkS-974 cells 12 h after seeding. The cocultured cells were stimulated with the indicated amounts of IFNγ for 48 to 72 h, and the proliferation of CTLL-2 was assessed via microscopic observation and quantitatively analyzed using the CellTiter-Glo® assay (Promega #G7570).

### ELISpot Assay.

ELISpot assay was performed using a 96-well plate pre-coated with a monoclonal anti-human IFNγ antibody, according to the manufactured instruction (R&D Systems #EL285), to detect activated T cells. In short, the indicated numbers of Jurkat-CD8/anti-ESO-TCR cells were stimulated with 100 ng/mL PMA and 300 ng/mL calcium ionophore (PMA/Ca), with 100 ng/mL anti-CD3ε antibody, or medium only (−) for 13 h in a 96-well plate, and the secreted IFNγ was captured on the PVDF membrane, detected by another anti-human IFNγ antibody, and visualized with alkaline phosphatase and BCIP/NBT.

### Dot Blot Assay.

GRk-974, GRkSB-043, and GRkSBZ-188 cells in a 24-well plate (1 × 10^5^ cell/well) were treated with IFNγ for 24 h. Cell lysates were extracted using 150 μL of GLO lysis buffer (Promega #E2661), and the 5 μg protein was dotted onto the nitrocellulose membrane. The B7-1 proteins were detected using an anti-B7-1 antibody (Proteintech #65083-1-Ig) at 1:500 dilution and visualized with HRP-conjugated anti-mouse IgG antibody and ECL reagent.

### RT-PCR.

GRk-974, GRkSB-043, and GRkSBZ-188 cells in a 24-well plate (1 × 10^5^ cell/well) were treated with IFNγ for 24 h. Total RNA was prepared from the cells using the acid phenol-guanidinium thiocyanate method. Reverse transcription was conducted for 60 min at 42 °C from 1 μg of total RNA using ProtoScript II Reverse Transcriptase (NEB # M0368L) and subjected to PCR using the specific primers (for anti-CD3ε-scFv-TM) listed in *SI Appendix*, Table S1 and KOD-FX-NEO DNA Polymerase (TOYOBO #KFX-201) on a LightCycler 1.5 (ABI). The reaction conditions were 94 °C for 2 min, followed by 30 cycles of 10-s denaturation at 98 °C and 30 s of extension at 68 °C.

#### Statistical Analysis.

Quantitative data were analyzed using Student’s *t* test with a two-tailed *P*-value. The n for each analysis is indicated in the figure legends. *P* < 0.01 was considered to indicate statistical significance.

## Supplementary Material

Appendix 01 (PDF)

## Data Availability

All study data are included in the article and/or *SI Appendix*.
