# Supplementary material for: Development of a highly sensitive platform for protein–protein interaction detection and regulation of T cell function
Source: Proc Natl Acad Sci U S A. 2024 Aug 6;121(33):e2318190121. doi: 10.1073/pnas.2318190121 (PMC11331103; doi:10.1073/pnas.2318190121)
Supplement: Supplementary file 1 — Appendix 01 (PDF) [file pnas.2318190121.sapp.pdf]

Supplementary Information

Materials and Methods

Firefly luciferase assay

A GAS (gamma interferon activation site)-containing promoter region or 4xISRE was placed before a reporter Firefly luciferase gene (20). The Firefly luciferase reporter plasmid (GAS-FireflyLuc or ISRE-FireflyLuc, 40 ng each) was transfected to HEK293T or HEK293T stably expressing RIG-I cells (2×10^4 per well) which were plated in a 96-well plate in advance, using Lipofectamine 3000 (Invitrogen). After 24 h, the cells were stimulated with indicated amounts of IFNγ or IFNα, respectively, for 12 h. Firefly luciferase activity was determined.

Establishment of stable cell line GRkh-134

To knock out endogenous HLA-A, -B, -C, and IFNAR1, we transfect GR-535 cells with plasmids to express Cas9, sgRNAs targeting common sites of HLA-A, -B, and -C genes (CGGCTACTACAACCAGAGCG) and targeting IFNAR1 gene (CCAGATGATGGTCGTCCTCC) with a IFNAR1 donor dsDNA to express puromycin resistance gene after successful recombination (see Supplementary Fig. 9). After selection with 2.8 μg/mL of puromycin for two weeks, the cells that did not respond to IFNα stimulation nor NY-ESO-1 peptide load against Jurkat-NFAT-Nluc/anti-ESO-scFv-28BBZ were isolated (GRkh-134).

ELISA assay

To measure IFNγ secreted from activated T cells, an ELISA assay was performed according to the manufactured instruction (PeproTech #900-K27). In short, the indicated numbers of Jurkat-CD8/anti-ESO-TCR cells were treated with 100 ng/mL PMA and 300 ng/mL calcium ionophore (PMA/Ca), or medium only (-) for 13 h in a 96-well plate. The supernatants were collected, and applied to a 96-well plate pre-coated with a monoclonal anti-human IFN-γ antibody. The IFNγ secreted in the medium was captured, detected by another anti-human IFNγ antibody, and visualized with HRP and TMB. The color development was measured by a plate reader at 404 nm, calibrated with a series of purified IFNγ; the IFNγ concentration was calculated using a third-degree polynomial equation.

Supplementary Figures

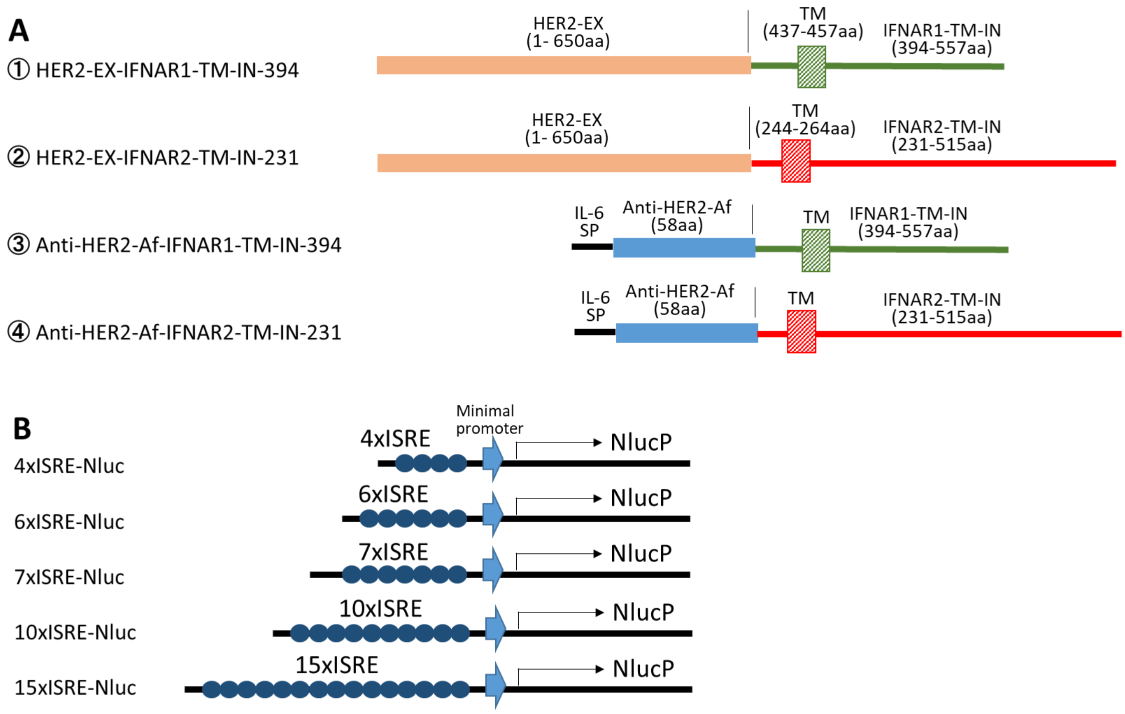

Supplementary Fig. 1

Chimeric receptor constructs for IFNARRS using an extracellular domain of HER2 and anti-HER2 affibody. A. An extracellular domain of HER2 (HER2-EX) and anti-HER2 affibody (anti-HER2 Af) were fused to the transmembrane and intracellular domains of IFNAR1 and IFNAR2 (IFNAR1-TM-IN and IFNAR2-TM-IN) ([1]-[4]), as in Fig. 1B. To express anti-HER2 affibody extracellularly, an IL-6 signal peptide (29 amino acids) was added at the N-terminus. Their DNA sequences are listed in Supplementary Table 2. **B. Constructs of nxISRE NlucP gene.** Indicated numbers of ISRE were placed immediately before the minimal promoter of the NlucP gene (a destabilized form of Nluc with a PEST sequence to respond more quickly to changes in transcriptional activity). They were used in Fig. 1D. Their DNA sequences are listed in Supplementary Table 2. EX, extracellular domain; TM, transmembrane region; IN, intracellular domain; PM, plasma membrane; IL-6 SP. IL-6 signal peptide; ISRE, interferon-stimulated response element.

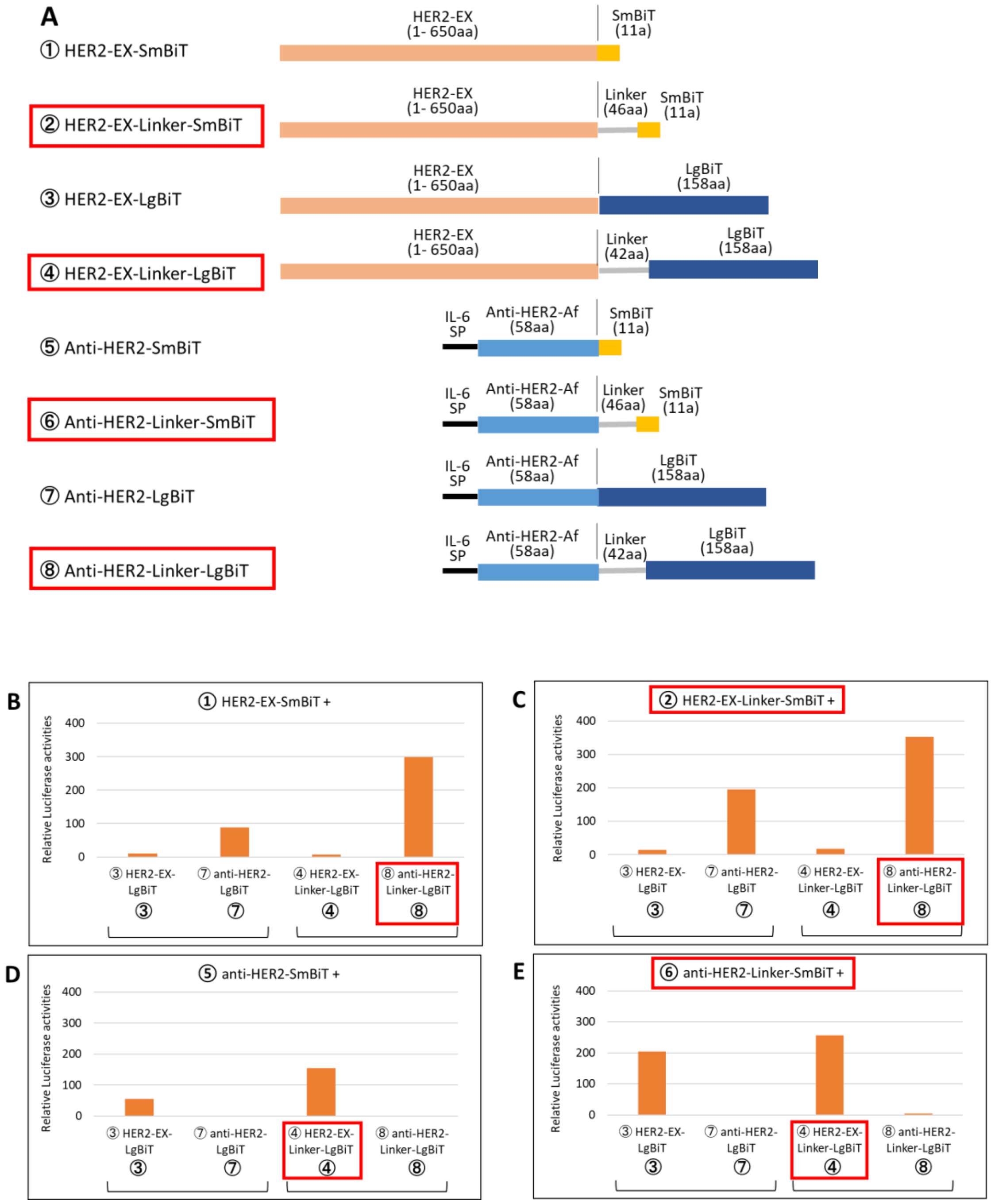

Supplementary Fig. 2

Constructs of HER2-EX- and anti-HER2 affibody-fused SmBiT and LgBiT with or without a flexible linker peptide. A. We constructed HER2-EX- and anti-HER2 affibody-fused SmBiT and LgBiT with or without a flexible linker peptide for comparative analysis of IFNARRS (IFNAR1/2 reconstitution system). Their DNA sequences are listed in Supplementary Table 3. **B-E.** Each pair, including SmBiT and LgBiT, was transfected into HEK293T cells. Nluc activities were measured 36 h after transfection. The reconstituted Nluc activities by the binding of HER2-EX and anti-HER2 affibody were increased with the insertion of a flexible linker peptide (enclosed in red). They were used in Fig. 2A and C.

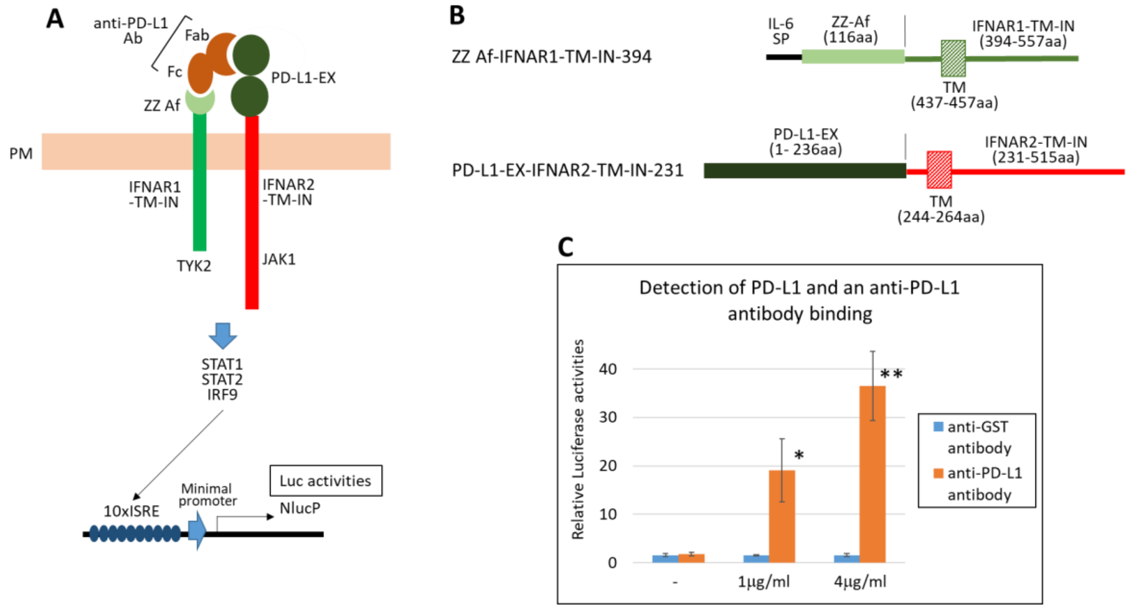

Supplementary Fig. 3

Interaction of PD-L1 with an anti-PD-L1 antibody. A. A ZZ-affibody (ZZ Af) was fused to IFNAR1-TM-IN to capture the Fc region of an anti-PD-L1 antibody (Ab), and an extracellular domain of PD-L1 (PD-L1-EX) was fused to IFNAR2-TM-IN. To express ZZ affibody extracellularly, an IL-6 signal peptide (29 amino acids) was added at the N-terminus. The constructs are shown in **B.** Their DNA sequences are listed in Supplementary Table 4. When the anti-PD-L1 antibody is added, a link is established between PD-L1-EX and ZZ Af, and Nluc activities are induced by proximity of the IFNAR1-TM-IN and IFNAR2-TM-IN domains. **C.** HEK293T cells seeded in a 96-well plate were transfected with the plasmids to express ZZ Af-IFNAR1-TM-IN, PD-L1-EX-IFNAR2-TM-IN, and 10xISRE-Nluc. After 24 h, the indicated amounts of the anti-PD-L1 antibody or control anti-GST antibody were added to the cells. Nluc assay was performed after 12 h of incubation with the antibodies (mean ± sd of three independent experiments). The PD-L1 antibody but not the control anti-GST antibody activated the luciferase activities significantly. Asterisks indicate significant difference compared with the control (*$p<0.01$, **$p<0.001$). ZZ Af, ZZ affibody that binds to the Fc region of immunoglobulin G; and PD-L1, programmed cell death ligand 1.

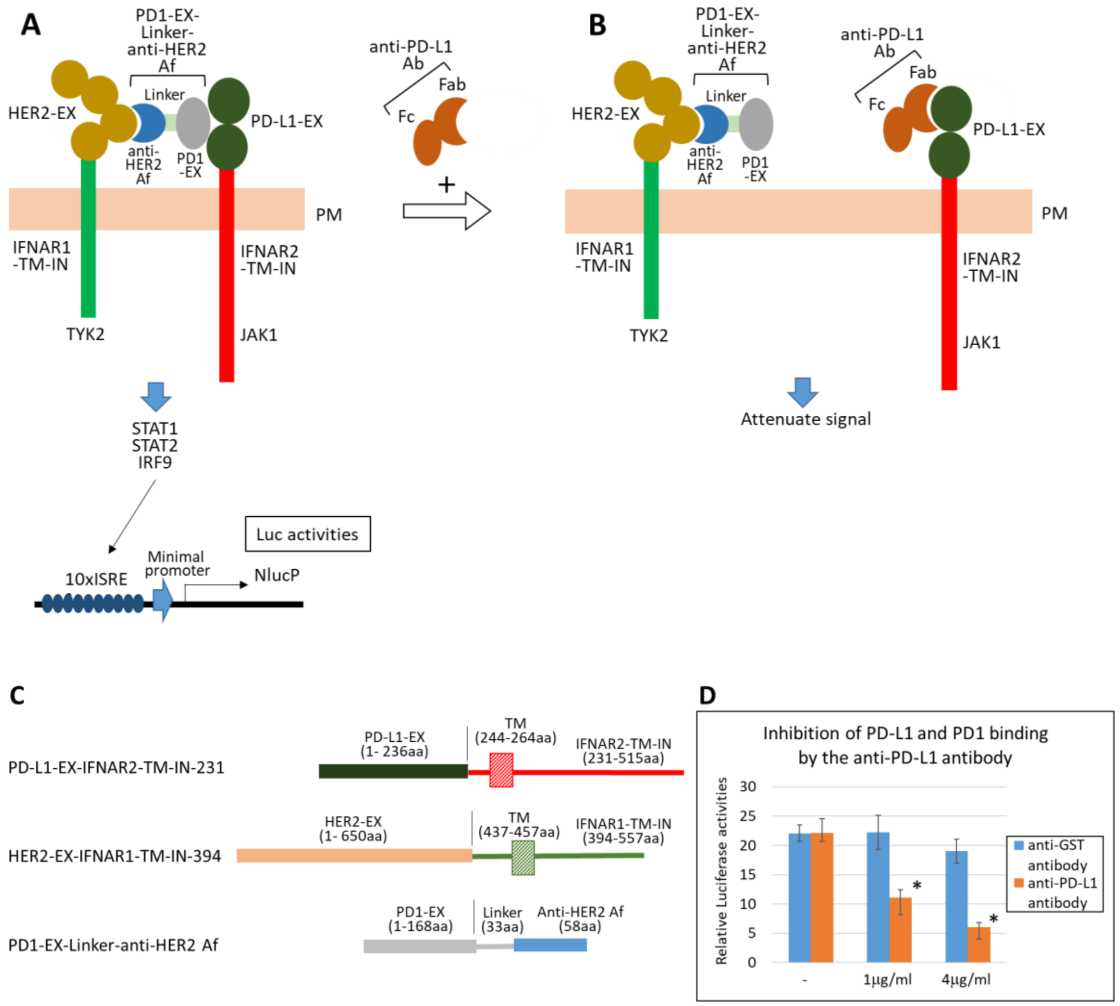

Supplementary Fig. 4

Inhibition of PD-L1 and PD1 binding by the anti-PD-L1 antibody. A. When PD1-EX linked to anti-HER2 Af via a short linker peptide (PD1-EX-Linker-anti-HER2 Af), HER2-EX-IFNAR1-TM-IN, and PD-L1-EX-IFNAR2-TM-IN, were expressed, Nluc activities are induced by the proximity of the IFNAR1-TM-IN and IFNAR2-TM-IN domains. The constructs are shown in **C.** Their DNA sequences are listed in Supplementary Table 4. **B.** When the anti-PD-L1 antibody inhibits the binding of PD-L1 and PD1, Nluc activities will attenuate. **D.** HEK293T cells seeded in a 96-well plate were transfected with the plasmids to express PD1-EX-Linker-anti-HER2 Af, HER2-EX-IFNAR1-TM-IN, PD-L1-EX-IFNAR2-TM-IN, and 10xISRE-Nluc. After 24 h, the indicated amounts of anti-PD-L1 antibody or control anti-GST antibody were added, Nluc assay was performed after 12 h of incubation with the antibodies (mean ± sd of three independent experiments). The PD-L1 antibody but not the control anti-GST antibody inhibited the luciferase activities significantly. Asterisks indicate significant difference compared with the control (*$p<0.001$). PD1, programmed cell death 1.

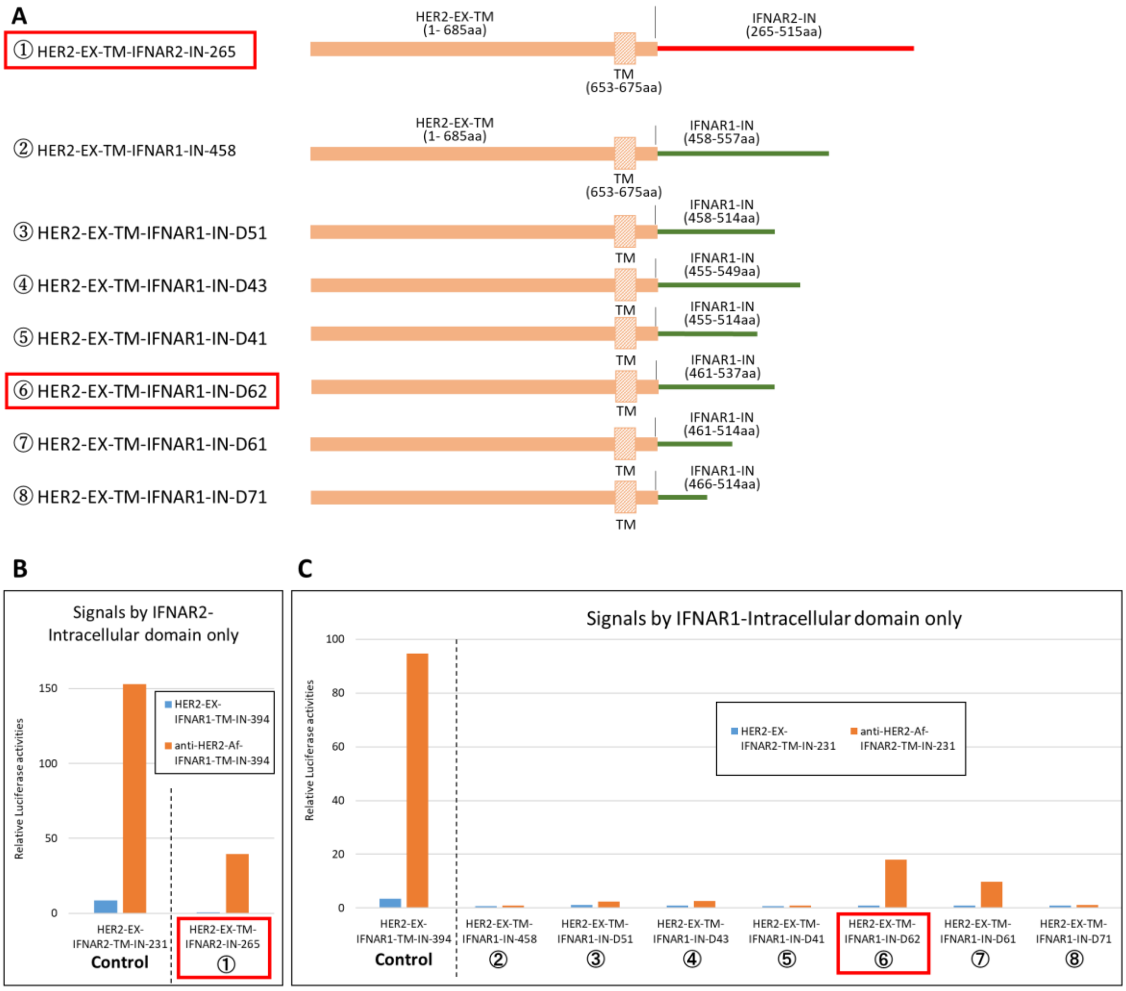

Supplementary Fig. 5

Intracellular domains of IFNAR1/2 are sufficient for signal transmittance (IFNARRS). A. The extracellular and transmembrane regions of HER2 (HER2-EX-TM) were fused to the IFNAR1 and IFNAR2 intracellular domains (IFNAR1-IN and IFNAR2-IN). We used a series of deletion mutants of the IFNAR1 and IFNAR2 intracellular domains ([1]-[8]) as conjugation of unrelated two domains sometimes impair the function of each domain. Their DNA sequences are listed in Supplementary Table 5. **B.** [1] HER2-EX-TM-IFNAR2-IN-265 (265-515 amino acid) worked well for the transmittance of the JAK-STAT signal when co-transfected with 6xISRE-NlucP into HEK293T cells, with anti-HER2-IFNAR1-TM-IN-394 (enclosed in red). HER2-EX-IFNAR2-TM-IN-231 was used as positive control. Nluc activities were measured 36 h after transfection. **C.** [6] HER2-EX-TM-IFNAR1-IN-D62 (461-537 amino acid) worked well for the transmittance of the JAK-STAT signal when co-transfected with 6xISRE-NlucP into HEK293T cells, with anti-HER2-IFNAR2-TM-IN-231 (enclosed in red). HER2-EX-IFNAR1-TM-IN-394 was used as positive control. Nluc activities were measured 36 h after transfection. The intracellular domains of IFNAR2 (265-515 amino acid) and IFNAR1 (461-537 amino acid) are sufficient for signal transmittance. The [1] and [6] were used in Fig. 2E and F.

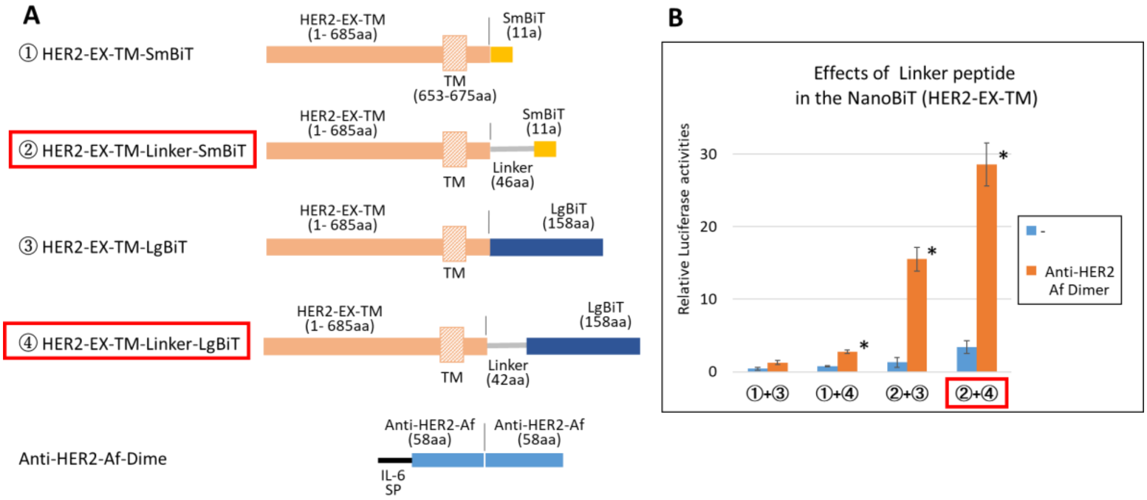

Supplementary Fig. 6

HER2-EX-TM dimer formation by anti-HER2 affibody dimer in NanoBiT system. A. Constructs of [1] HER2-EX-TM-SmBiT, [2] HER2-EX-TM-Linker-SmBiT, [3] HER2-EX-TM-LgBiT, and [4] HER2-EX-TM-Linker-LgBiT for the NanoBiT assay. Their DNA sequences are listed in Supplementary Table 6. **B.** HEK293T cells seeded in a 96-well plate were transfected with the indicated pairs of plasmids (each pair containing SmBiT and LgBiT) with or without the anti-HER2 affibody dimer. Reconstituted Nluc activities were measured 36h after transfection (mean ± sd of three independent experiments). By inserting the linkers enclosed in red, the signals were enhanced ([2] and [4]). They were used in Fig. 2D and F. Asterisks indicate significant difference compared with the control (*p<0.001).

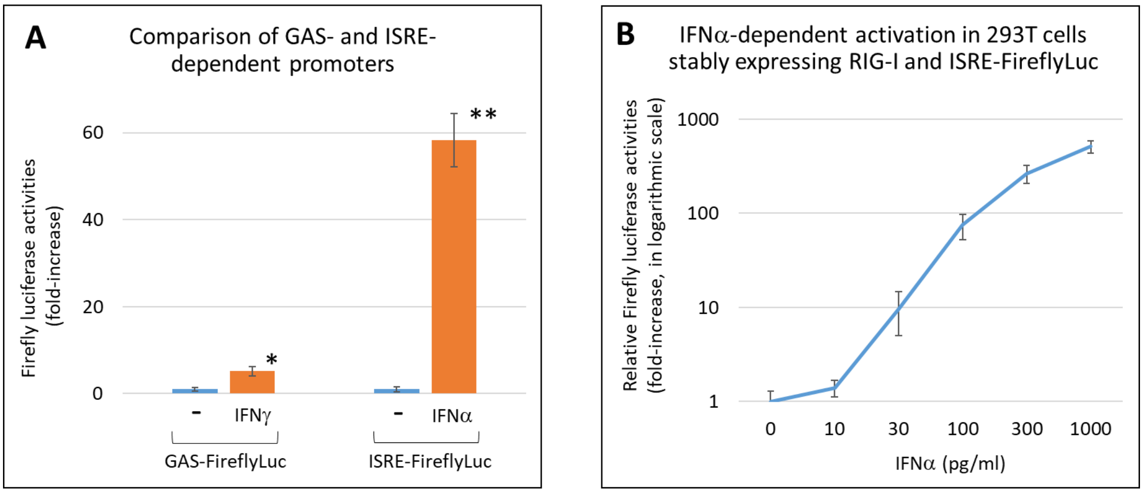

Supplementary Fig. 7

Comparison between GAS- and ISRE-dependent promoters. **A**. The Firefly luciferase reporter plasmid (GAS-FireflyLuc or ISRE-FireflyLuc, 40 ng each) was transfected to HEK293T cells in a 96-well plate. After 24 h, the cells were stimulated with 1,000 pg/mL of IFNγ or IFNα, respectively, for 12 h. Firefly luciferase activities were measured. Asterisks indicate a significant difference from the control (*$p < 0.01$, **$p < 0.00001$). **B.** HEK293 cells stably expressing RIG-I and an ISRE-FireflyLuc gene were treated in a 96-well plate with indicated amounts of IFNα for 12 h. Firefly luciferase activities were measured and shown, taking medium only as 1 on a logarithmic scale. The IFN-dependent activation of luciferase activities was significantly enhanced.

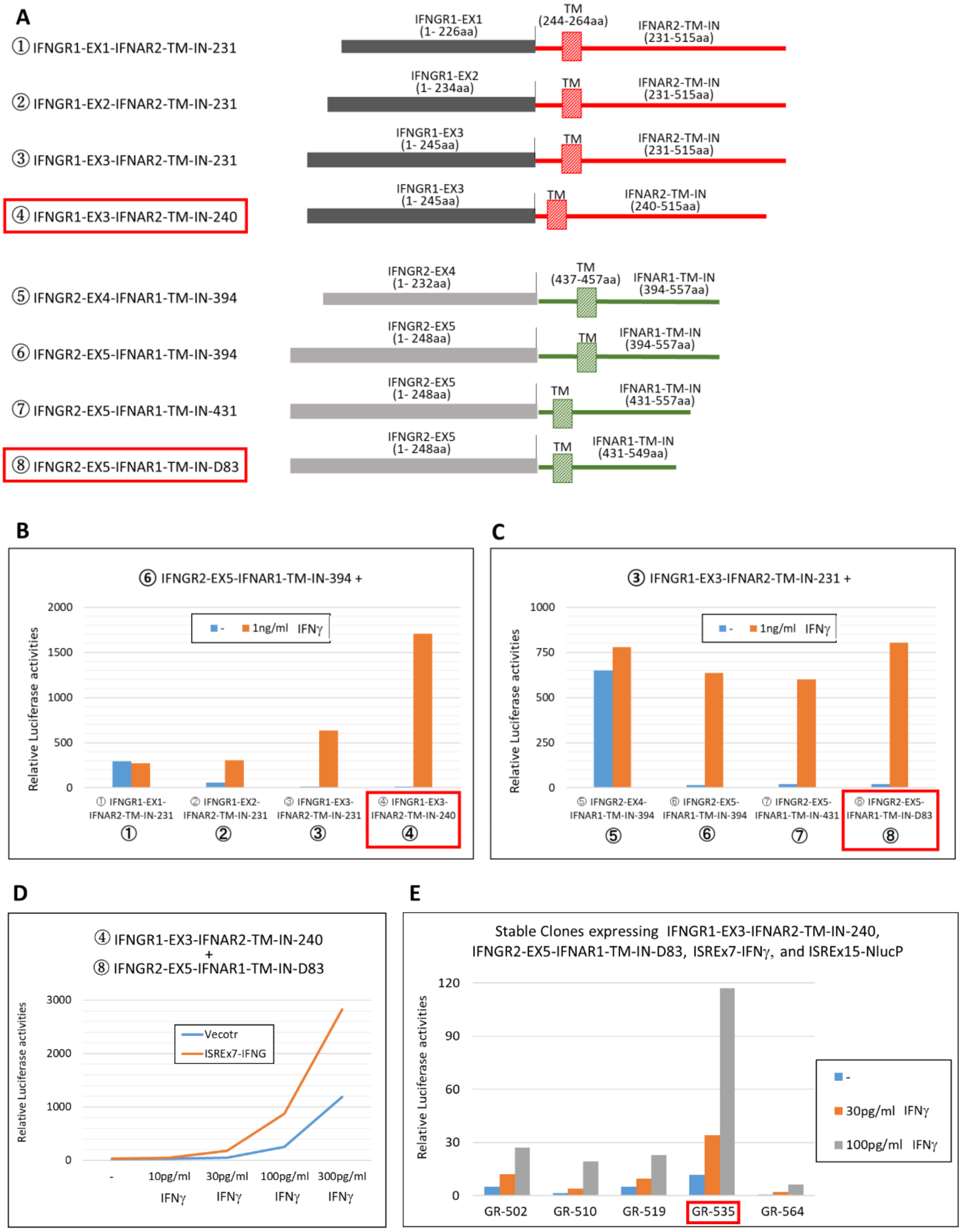

Supplementary Fig. 8

Application for the ligand-dependent clustering of IFNARRS (IFNGR1/IFNGR2 complex formation). **A.** Different lengths of the extracellular regions of IFNGR1 and IFNGR2 were fused to the transmembrane and intracellular regions of IFNAR2 and IFNAR1, respectively ([1]-[8]). Their DNA sequences are listed in Supplementary Table 7. **B.** Among the four IFNGR1-EX containing constructs ([1]-[4]), [4] resulted in the best signal/noise ratio for IFNγ-dependent activation when co-transfected with IFNGR2-EX5-IFNAR1-TM-IN-394 ([6]) and 10xISRE-NlucP

into HEK293T cells (enclosed in red). The cells were treated with the indicated concentration of IFNγ for 12h after 24 h of transfection. The Nluc assay was performed. **C.** Among the four IFNGR2-EX- containing constructs ([5]-[8]), [8] resulted in the best signal/noise ratio for IFNγ-dependent activation for 12 h after transfection, when co-transfected with IFNGR1-EX3-IFNAR2-TM-IN-231 ([3]), and 10xISRE-NlucP into HEK293T cells (enclosed in red). **D.** The best combination of [4] (IFNGR1-EX-IFNAR2-TM-IN-240) and [8] (IFNGR2-EX-IFNAR1-TM-IN-D83), and 10xISRE-NlucP were transfected with or without 7xISRE-IFNγ. 7xISRE-IFNγ enhanced the IFNγ-dependent activation signals. They were schematically illustrated in Figs. 3A and 4A. **E.** To obtain stable cell lines, [4] and [8] were transfected with 15xISRE-NlucP, 7xISRE-IFNγ, and a hygromycin resistance gene-expressing plasmid. After hygromycin B selection, the stable cell line, GR-535 (enclosed in red), which exhibited the highest signal/noise ratio (IFNγ-dependent activation), was isolated among several stable cell lines. The isolated stable cell lines were seeded in a 96-well plate. Nluc assay was performed after treatment with the indicate concentrations of IFNγ for 12h.

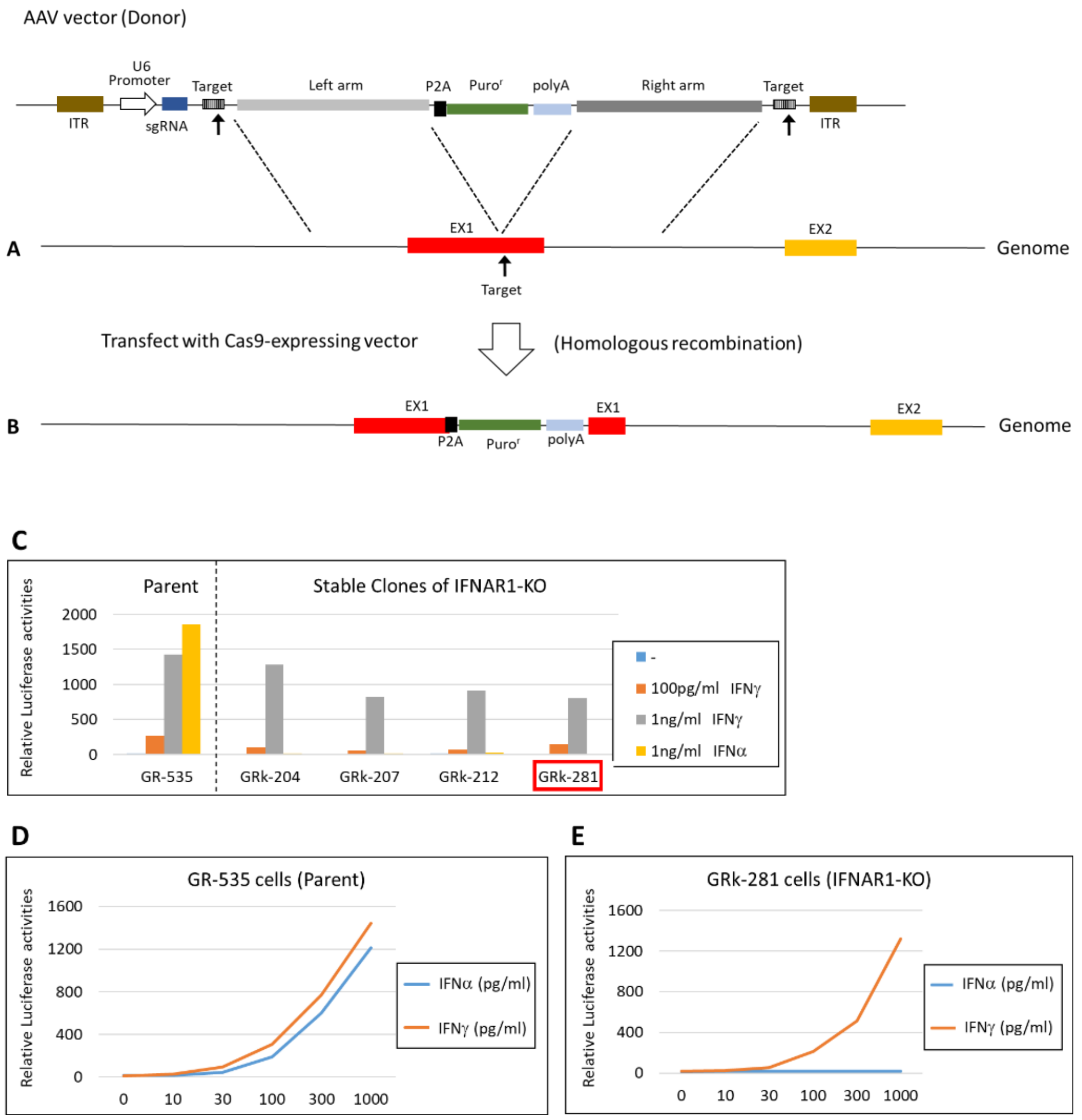

Supplementary Fig. 9

Knockout of the endogenous *IFNAR1* gene. We constructed a donor plasmid using an adeno-associated virus (AAV) vector with a specific sgRNA targeting the endogenous *IFNAR1* gene (**A**) in HEK293T cells. The target site is adjacent to the ATG initiation codon in exon1 (EX1). Their DNA sequences are listed in Supplementary Table 8. To facilitate homologous recombination, the targeting sequences indicated in arrows were also added adjacent to the homology arms for creating a double-cut donor dsDNA (25). The donor plasmid enabled expression of a puromycin resistance gene after successful homologous recombination (**B**). **C.** GR-535 cells were transfected with a Cas9-expressing plasmid and the sgRNA targeting IFNAR1 with donor dsDNA. After puromycin selection, the cell line, GRk-281(enclosed in red), which exhibited the highest signal/noise ratio (IFNγ-stimulated Nluc activities) and no response to IFNα via knockout of endogenous *IFNAR1*, was isolated among several stable cell lines. The parent (GR-535) cells and isolated stable cell lines were seeded in a 96-well plate. Nluc assay was performed after

treatment with the indicate concentrations of IFNγ or IFNα for 12h. **D,E.** Both GR-535 and GRk-281 cells exhibited IFNγ dose-dependent activation for 12 h; however, IFNα-dependent activation in GR-535 was completely abolished in GRk-281 cells. ITR, inverted terminal repeat of AAV; P2A, porcine teschovirus-1 2A self-cleaving peptide; $Puro^r$, puromycin resistance gene; polyA, a polyadenylation signal sequence; EX1, exon1 of the endogenous *IFNAR1* gene.

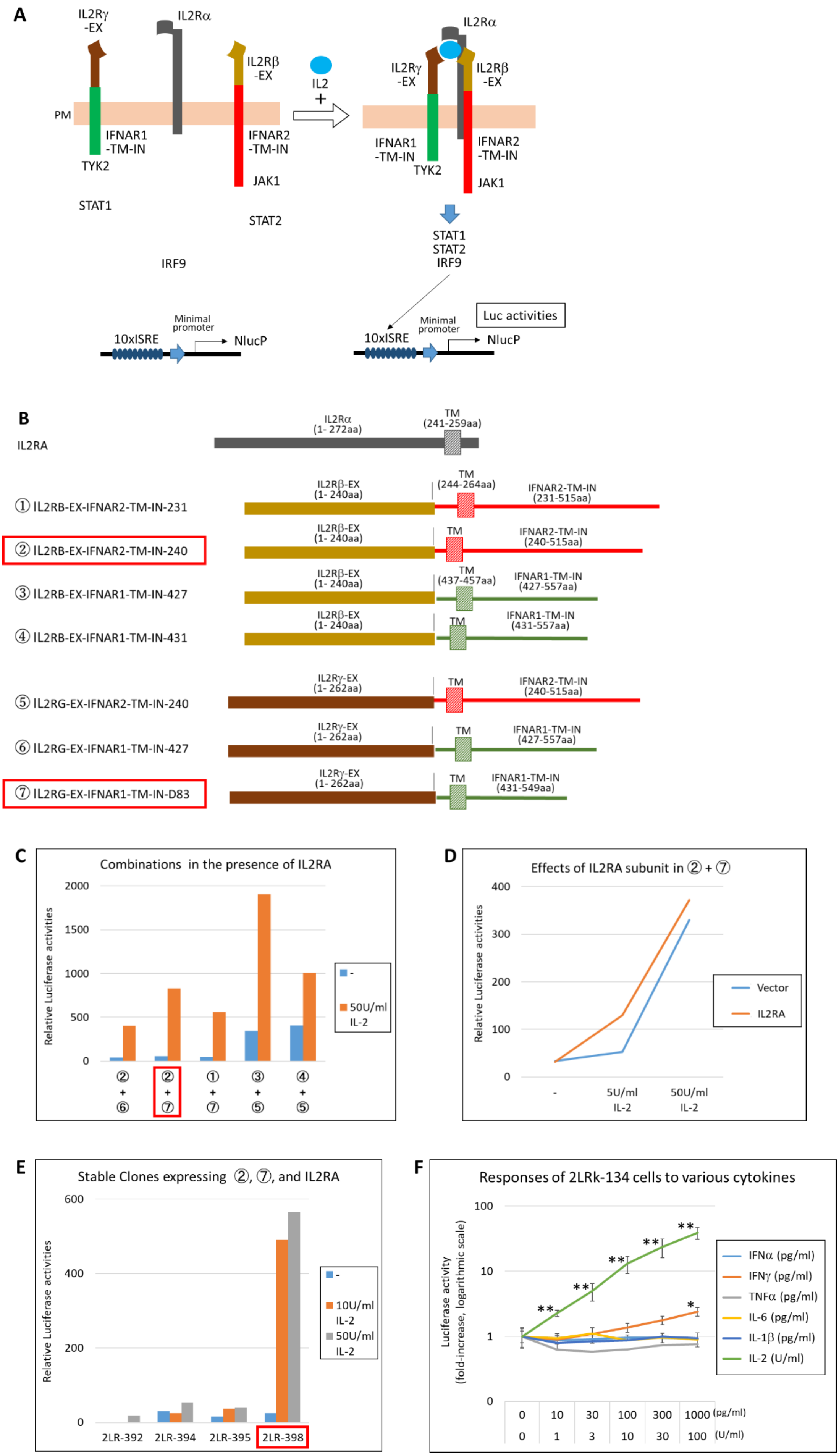
A
IL2Rγ -EX
IL2Rα
IL2Rβ -EX
IL2
+
PM
IFNAR1 -TM-IN
TYK2
IFNAR2 -TM-IN
JAK1
STAT1
STAT2
IRF9
Luc activities
10xISRE
Minimal promoter
NlucP
B
IL2RA
IL2Rα (1- 272aa)
TM (241-259aa)
① IL2RB-EX-IFNAR2-TM-IN-231
② IL2RB-EX-IFNAR2-TM-IN-240
③ IL2RB-EX-IFNAR1-TM-IN-427
④ IL2RB-EX-IFNAR1-TM-IN-431
⑤ IL2RG-EX-IFNAR2-TM-IN-240
⑥ IL2RG-EX-IFNAR1-TM-IN-427
⑦ IL2RG-EX-IFNAR1-TM-IN-D83
IL2Rβ-EX (1- 240aa)
IL2Rγ-EX (1- 262aa)
TM (244-264aa)
TM (437-457aa)
IFNAR2-TM-IN (231-515aa)
IFNAR2-TM-IN (240-515aa)
IFNAR1-TM-IN (427-557aa)
IFNAR1-TM-IN (431-557aa)
IFNAR1-TM-IN (431-549aa)
C
Combinations in the presence of IL2RA
Relative Luciferase activities
-
50U/ml IL-2
②+⑥ ②+⑦ ①+⑦ ③+⑤ ④+⑤
D
Effects of IL2RA subunit in ② + ⑦
Relative Luciferase activities
Vector
IL2RA
5U/ml IL-2
50U/ml IL-2
E
Stable Clones expressing ②, ⑦, and IL2RA
Relative Luciferase activities
-
10U/ml IL-2
50U/ml IL-2
2LR-392 2LR-394 2LR-395 2LR-398
F
Responses of 2LRk-134 cells to various cytokines
Luciferase activity (fold-increase, logarithmic scale)
IFNα (pg/ml)
IFNγ (pg/ml)
TNFα (pg/ml)
IL-6 (pg/ml)
IL-1β (pg/ml)
IL-2 (U/ml)
0 10 30 100 300 1000 (pg/ml)
0 1 3 10 30 100 (U/ml)

Supplementary Fig. 10

Application for the ligand-dependent clustering of IFNARRS (IL2RA, IL2RB, and IL2RG complex formation). A. Extracellular domains of IL2RG and IL2RB (IL2Rγ-EX and IL2Rβ-EX) were fused to IFNAR1-TM-IN and IFNAR2-TM-IN, respectively. When the IL2RG-IFNAR1-TM-IN and IL2RB-IFNAR2-TM-IN are expressed with IL2RA, the extra domains of IL2RA, IL2RB, and IL2RG are clustered by the addition of IL-2. **B.** Different lengths of the extracellular regions of IL2RB and IL2RG were fused to the transmembrane and intracellular regions of IFNAR2 and IFNAR1 ([1]-[8]). Their DNA sequences are listed in Supplementary Table 9. **C.** We compared five combinations of the constructs by co-transfecting each combination with 2ILRA and 10xISRE-NlucP. After 24 h of transfection, [2] (IL2RB-EX-IFNAR2-TM-IN-240) + [7] (IL2RG-EX-IFNAR1-TM-IN-D83) induced the best signal/noise ratio for IL-2-dependent activation for 12 h (enclosed in red). **D.** Co-expression of the IL2RA subunit with [2] + [7] + 10xISRE-NlucP, enhanced IL-2-dependent activation. **E.** To obtain stable cell lines, [2] + [7] + IL2RA were transfected with 10xISRE-NlucP, and a hygromycin resistance gene-expressing plasmid. After hygromycin B selection, the stable cell line, 2LR-398 (enclosed in red), which exhibited the highest signal/noise ratio (IL-2-stimulated Nluc activities), was isolated among several stable cell lines. Nluc assay was performed after treatment with the indicate concentrations of IL-2 for 12h. **F.** 2LRk-134 cells stably expressing IL2RA, IL2RB-EX-IFNAR2-TM-IN, IL2RG-EX-IFNAR1-TM-IN, and 10xISRE-NlucP, were established by knocked out of the endogenous *IFNAR1* gene in 2LR-398 cells. 2LRk-134 cells were stimulated with the indicated concentrations of various cytokines for 12 h. The Nluc assay was then performed and shown, taking medium only as 1 on a logarithmic scale (mean ± sd of three independent experiments). 2LRk-134 cells exhibited dose-dependent responses to IL-2 (1-100 U/mL), with no responses to other cytokines except a very high concentration of IFNγ (1,000 pg/mL). Asterisks indicate significant difference compared with the control (*$p<0.01$, **$p<0.0001$). IL-2, interleukin 2; IL2RA, IL2RB, and IL2RG, interleukin 2 receptor subunit alpha, beta, and gamma, respectively.

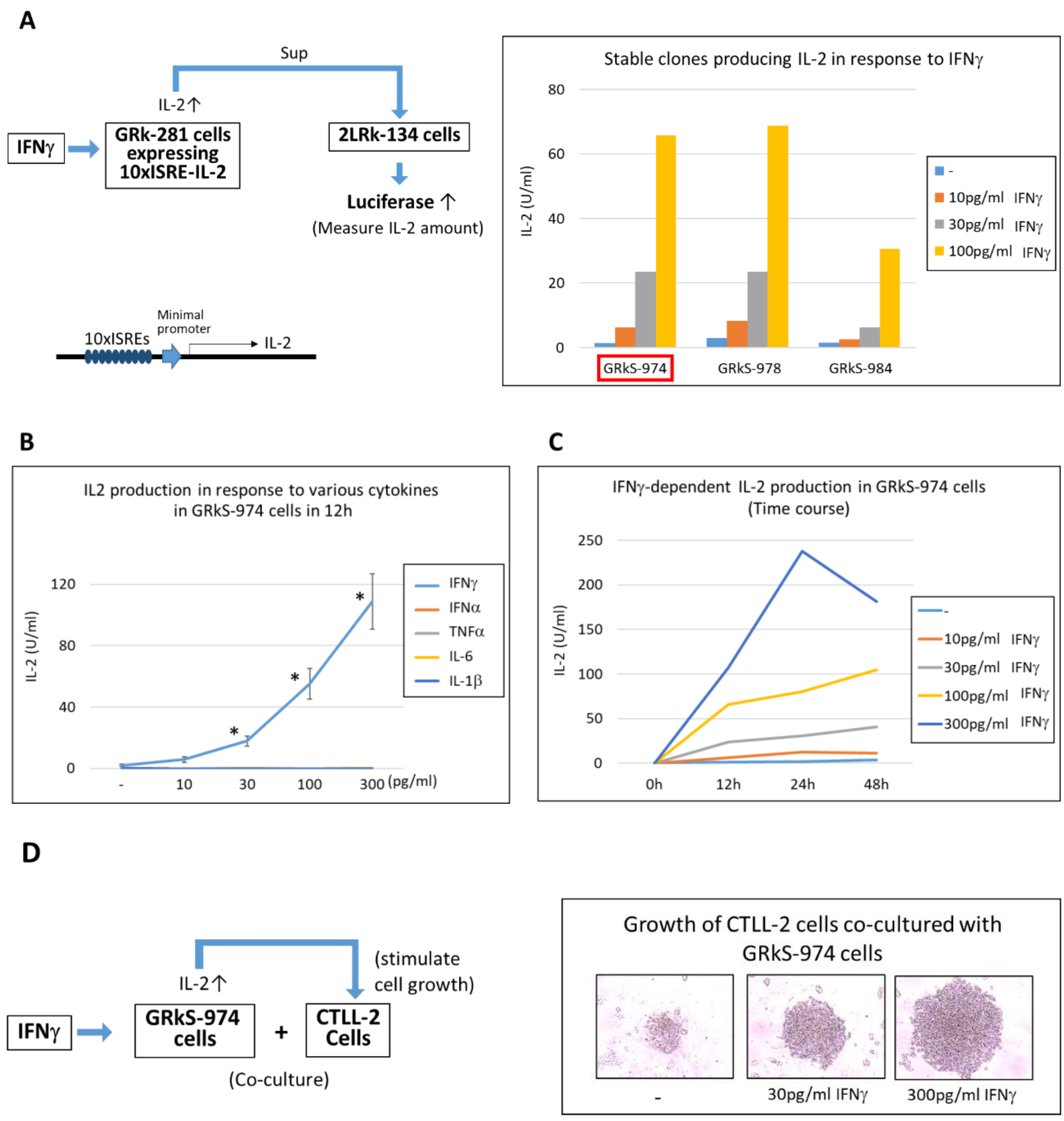

Supplementary Fig. 11. Stable GRkS-974 cells secreted IL-2 in response to IFN-γ. A. IL-2 production was measured using 2LRk-134 cells. To confer IL-2 production in response to IFNγ, GRk-281 cells (Supplementary Fig. 9C and E) were further transfected with 10xISRE-IL-2 (Supplementary Table 10) and a blasticidin resistance gene-expressing plasmid. After blasticidin S selection, the GRkS-974 cell line (enclosed in red), which exhibited the highest signal/noise ratio (IFNγ-dependent IL-2 production), was established among several stable cell lines. The concentrations of IL-2 were estimated by adding the supernatants of the stable cell lines that were treated with the indicated concentrations of IFNγ for 24 h to 2LRk-134 cells (Supplementary Fig. 10F). 2LRk-134 cells responded according to the amount of IL-2 in the supernatant. Nluc assay was performed after treatment with the supernatants for 12h, and the IL-2 concentration was calculated. **B.** The supernatants of GRkS-974 cells, after treatment with the indicated concentrations of various cytokines for 24 h, were added to 2LRk-134 cells to measure IL-2 levels. Nluc assay was

performed after treatment with the supernatants for 12h, and the IL-2 concentration was calculated (mean ± sd of three independent experiments). GRkS-974 cells secreted IL-2 responding only to IFNγ among the cytokines. **C.** GRkS-974 cells were treated with indicated IFNγ concentrations for the time indicated, and the supernatants were added to 2LRk-134 cells to measure IL-2 levels. Nluc assay was performed after 12h, and the IL-2 concentration was calculated. GRkS-974 cells secrete a large amount of IL-2 in response to 10-300 pg/mL IFNγ at least for 48 h after stimulation. **D**. CTLL-2 cells (a mouse cell line known to proliferate in response to IL-2) were co-cultured with GRkS-974 cells. When the cells (GRkS-974: 20, CTLL2: 400) were stimulated with the indicated concentrations of IFNγ, the CTLL-2 cells proliferated significantly (*$p < 0.0001$). Photos were captured 48 hours after stimulation.

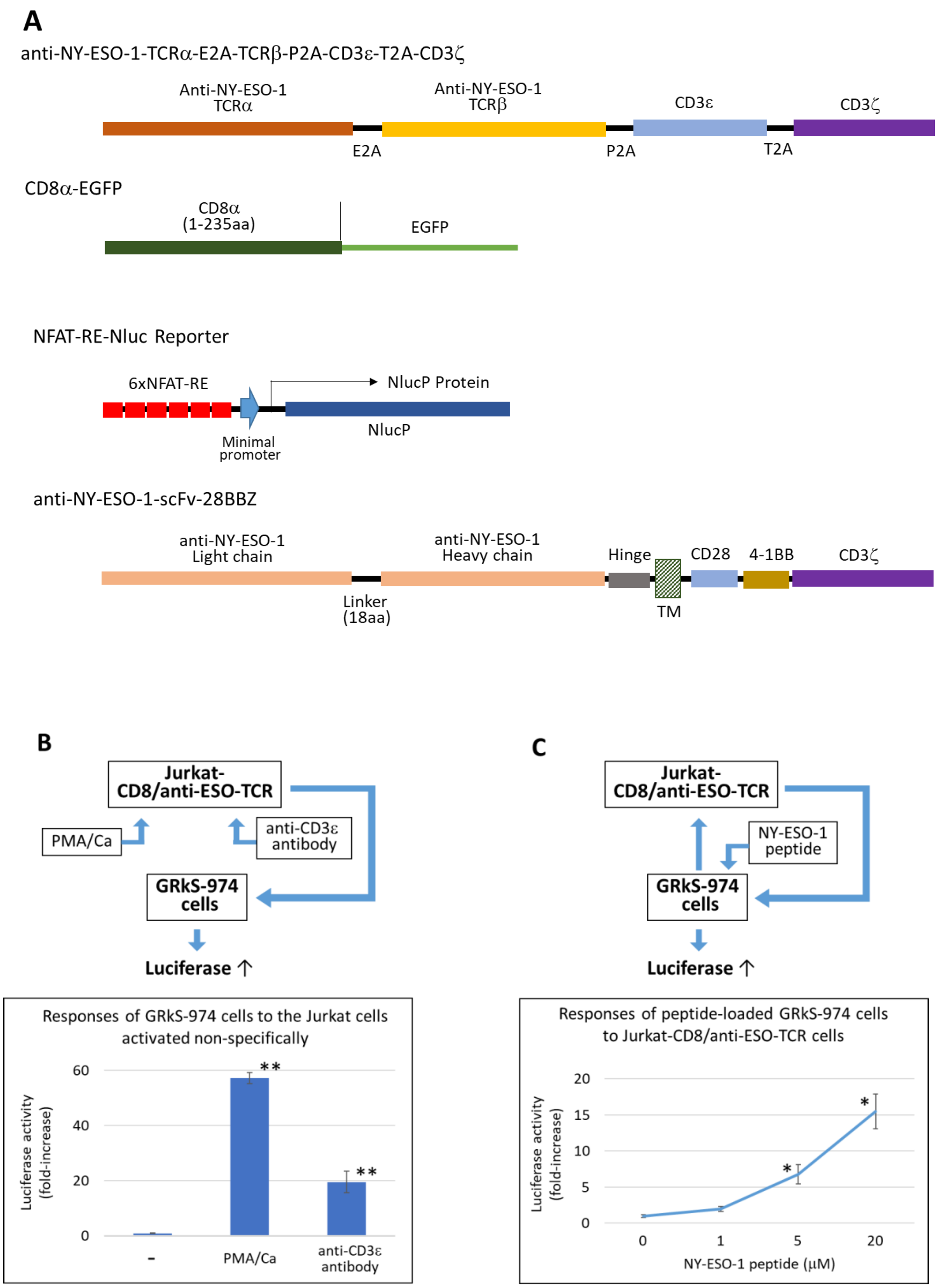

Supplementary Fig. 12. A. Vector constructs for recombinant virus. The lentiviruses were designed to express an anti-NY-ESO-1 TCRα/β with CD3ε/ζ and CD8α-EGFP (23) in Jurkat T cells. The variable regions of TCRα/β in pLentivirus for TCRα/β/CD3ε/ζ expression (24) was replaced with an anti-NY-ESO-1 TCRα/β (14). Six-tandem repeats NFAT-RE (30 bp) was used as NlucP reporter (29). For CAR-T, an anti-NY-ESO-1 scFv (3M4E5) (31) was fused to

a chimeric CD28-4-1BB-CD3ζ (32). Some DNA sequences are shown in Supplementary Table 11. **B.** Jurkat cells expressing CD8α-EGFP and anti-NY-ESO-1 TCRα/β were co-cultured with GRkS-974 cells seeded on a 96-well plate 12 h before addition of Jurkat cells. The co-cultured cells were stimulated with 100 ng/mL Phorbol 12-myristate 13-acetate (PMA) and 300 ng/mL calcium ionophore (Ca), or 100 ng/mL anti-CD3ε antibody (22) for 12 h. Nluc assay was performed and shown, taking the medium only (-) as 1 on a logarithmic scale (mean ± sd of three independent experiments). **C.** GRkS-974 cells were seeded on a 96-well plate. After 12 h, the cells were treated with the indicated concentrations of the NY-ESO-1 peptide for 12 h, and then co-cultured with Jurkat cells expressing the CD8α-EGFP and anti-NY-ESO-1 TCRα/β for another 12 h. The Nluc assay was then performed (mean ± sd of three independent experiments). GRkS-974 cells showed significant activation of luciferase in response to those stimuli. Luciferase activity was shown taking the medium only as 1 (mean ± sd of three independent experiments). Asterisks indicate significant difference compared with the control (**$p<0.00001$, *$p<0.001$). E2A, P2A, and T2A, self-cleaving peptides derived from equine rhinitis A virus, porcine teschovirus-1, and thosea asigna virus, respectively. NFAT-RE, Nuclear factor of activated T cells-response element.

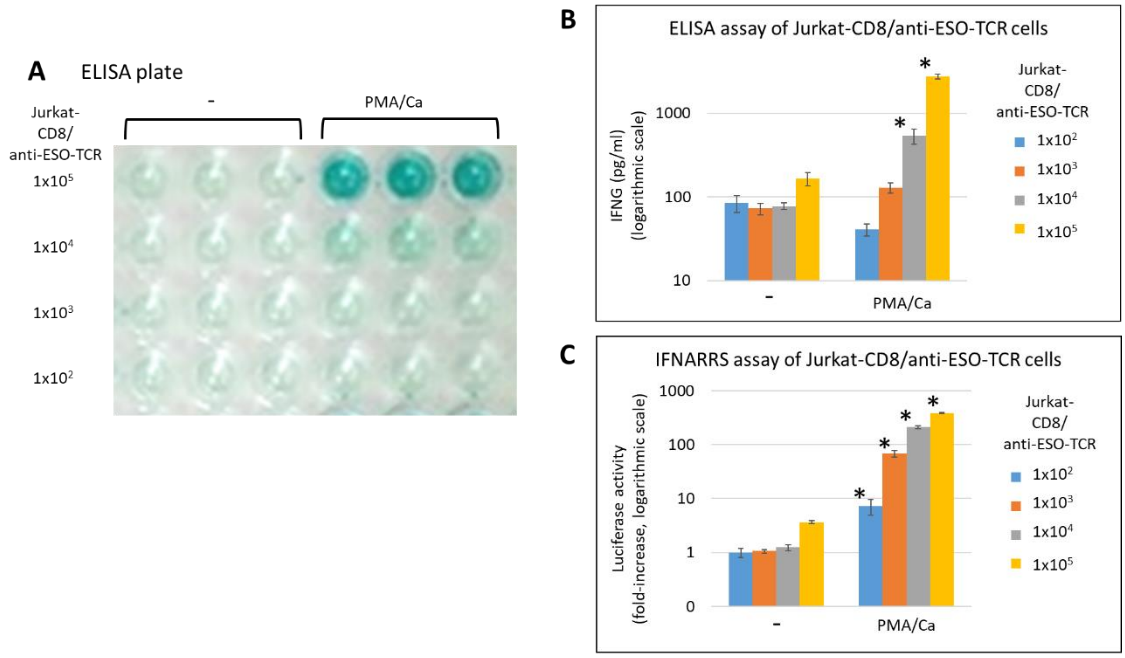

Supplementary Fig. 13. IFNARRS was compared with ELISA assay. A. Indicated numbers of Jurkat-CD8/anti-ESO-TCR cells were stimulated with 100 ng/mL PMA and 300 ng/mL calcium ionophore (PMA/Ca) or medium only (-) for 13 h in a 96-well plate. IFNγ secreted in the supernatants were captured by pre-coating a monoclonal anti-human IFNγ antibody on a 96-well plate for ELISA assay. The plate visualized with HRP and TMB is shown. **B.** A plate reader measured the color development at 404 nm, the IFNγ concentration was calculated and shown on a logarithmic scale (mean ± sd of three wells). **C.** Simultaneously, the same numbers of Jurkat-CD8/anti-ESO-TCR cells were added to the GRkS-974 cells seeded in a 96-well plate in advance (2 x 10^4 cells/well), as IFNARRS. After the same treatments as in **A**, Nluc assay was performed and shown, taking the count of medium only with 1 x 10^2 Jurkat cells as 1 on a logarithmic scale (mean ± sd of four independent experiments). Asterisks indicate a significant difference from the control (*$p < 0.0001$).

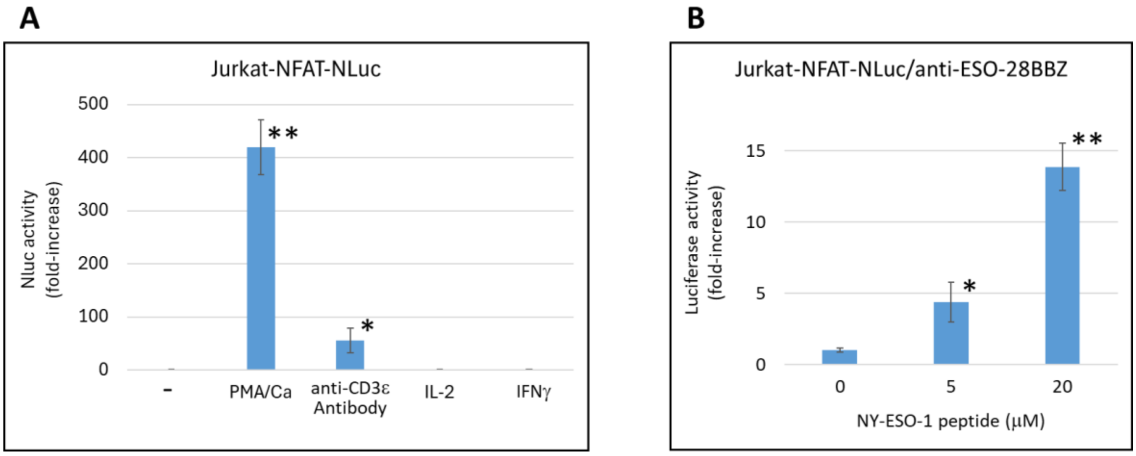

Supplementary Fig. 14. Jurkat-NFAT-Nluc and Jurkat-NFAT-Nluc/ani-ESO-28BBZ cells to monitor its TCR activation. A. Jurkat-NFAT-Nluc cells. To monitor TCR activation in T cell, NFAT-RE (nuclear factor of activated T cells-response element) was widely used (29). We constructed a stable Jurkat cell line expressing a NlucP gene under the control of 6xNFAT-RE (Jurkat-NFAT-Nluc). When the Jurkat-NFAT-Nluc cells were treated with PMA/Ca or anti-CD3ε antibody for 12 h, Nluc activities increased significantly. However, treatment with IL-2 or IFN-γ had no effects on the NFAT-RE. **B. Jurkat-NFAT-Nluc/anti-ESO-28BBZ cells.** To assess the peptide-MHC complex on HEK293T cells, Jurkat-NFAT-Nluc cells were further transduced with anti-NY-ESO-1-28BBZ (CAR-T). The resultant Jurkat-NFAT-Nluc/anti-ESO-28BBZ cells responded significantly to NY-ESO-1 peptide load to HEK293T cells. Asterisks indicate a significant difference from the control (*$p < 0.001$, **$p < 0.00001$).

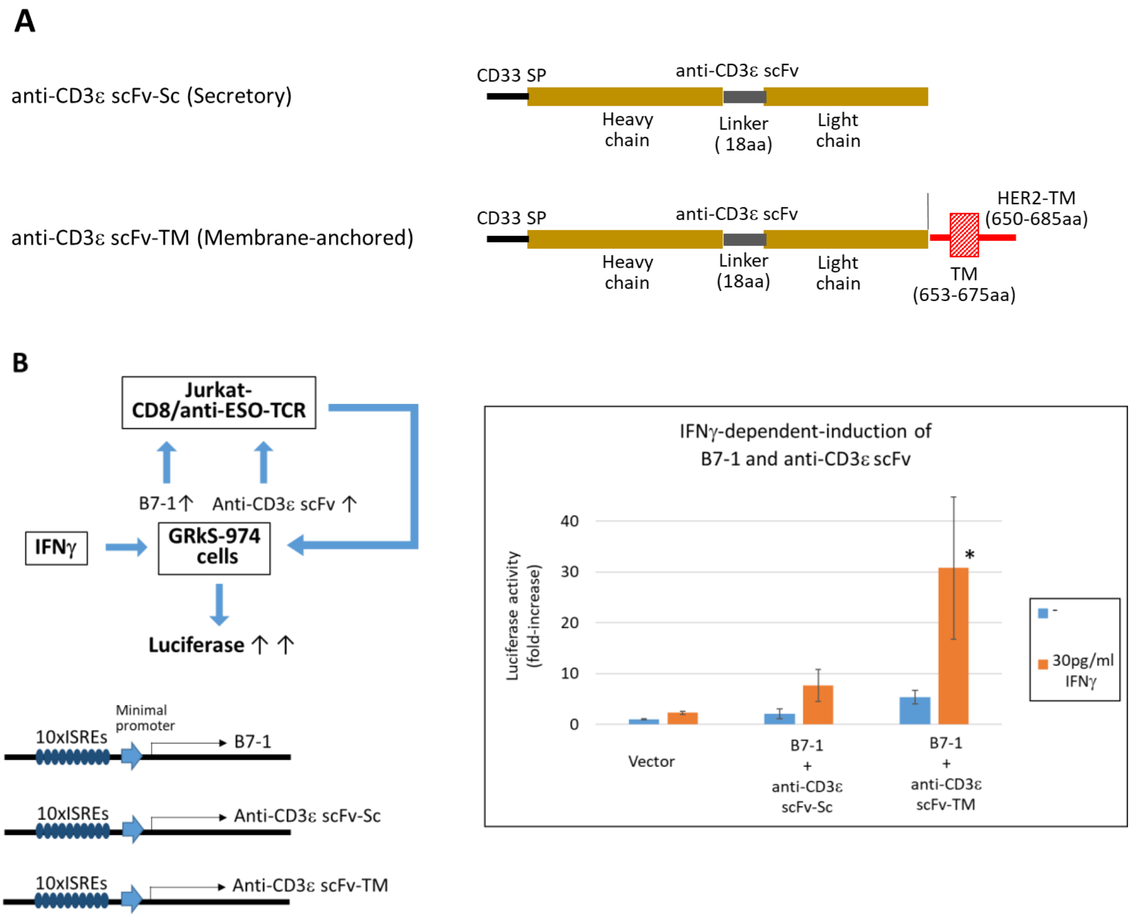

Supplementary Fig. 15. Activation with the anti-CD3ε antibody or anti-CD3ε scFv was significantly enhanced by B7-1. A. Constructs of anti-CD3ε scFvs. An anti-CD3ε scFv (27) was designed for secretion in the medium (anti-CD3ε scFv-Sc) by placing a stop codon after the end of the light chain. The same anti-CD3ε scFv was designed for retention on the cell surface (anti-CD3ε scFv-TM) by addition of a HER2 transmembrane region. To express the anti-CD3ε scFvs extracellularly, an CD33 signal peptide (SP, 17 amino acids) was added at the N-terminus. The DNA sequences are shown in Supplementary Table 12. **B.** As depicted, we opted to confer IFNγ-dependent expression of a co-stimulator, B7-1, anti-CD3ε scFv-Sc, and anti-CD3ε scFv-TM via ISRE in GRkS-974 cells. Thus, B7-1, anti-CD3ε scFv-Sc, and anti-CD3ε scFv-TM genes were placed under the 10xISRE promoter. GRkS-974 cells transfected with these plasmids were co-cultured with Jurkat cells expressing CD8α-EGFP and anti-NY-ESO-1 TCRα/β for 24 h with or without 30 pg/mL IFNγ after 24 h of transfection. Luciferase activities were significantly enhanced by co-transfection of B7-1 with anti-CD3ε scFv-TM in an IFNγ-dependent manner. Anti-CD3ε scFv-TM was more potent for signal activation than anti-CD3ε scFv-Sc. These results support the scheme in Fig. 9. Asterisks indicate significant difference compared with the control (*$p<0.01$). CD33 SP, a signal peptide of CD33.

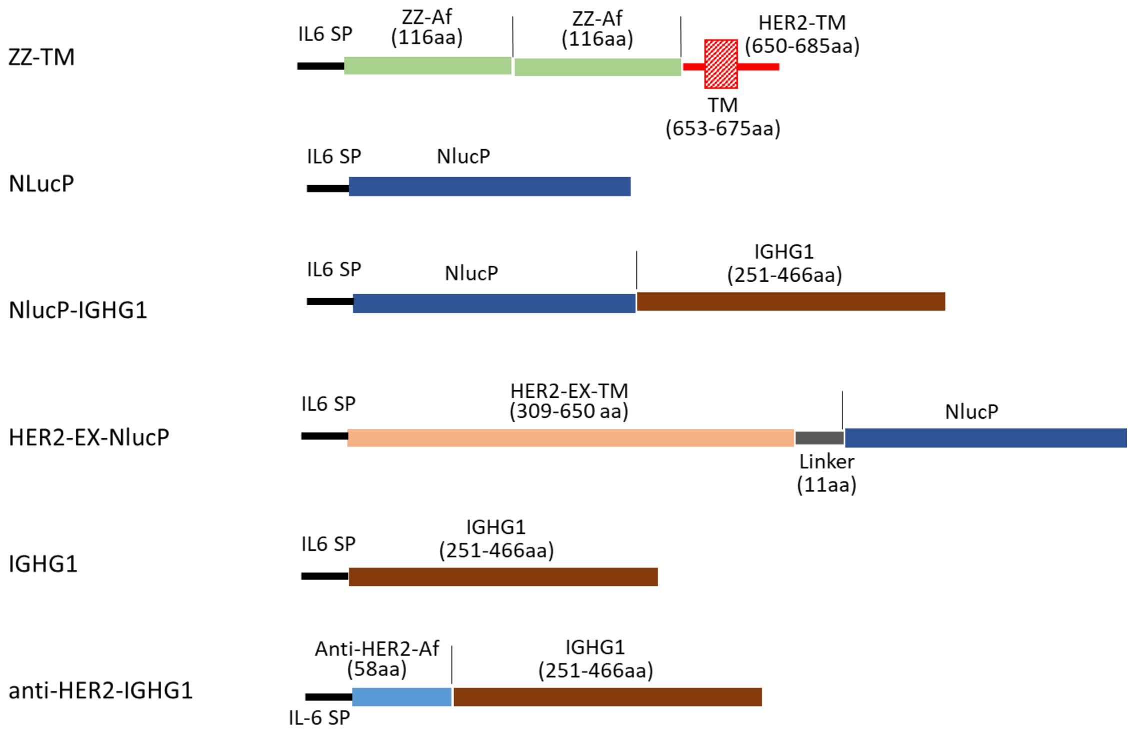

Supplementary Fig. 16. Constructs for the binding experiments in Fig. 7C and D. To express tandem ZZ affibodies on the cell surface, the IL-6 signal peptide, and the part of HER2, including the transmembrane region were added to the ZZ-Af. All other constructs are designed to secrete to the medium by adding the IL6 SP. CH2 and CH3 domains of IGHG1 (251-466 amino acids) that bind to the ZZ affibody and domains III and IV of HER2-EX (309-650 amino acids) that bind to the anti-HER2 affibody were used for the constructs. IGHG1, immunoglobulin heavy constant gamma 1 (28); HER2-EX, extracellular domain of HER2 (8).

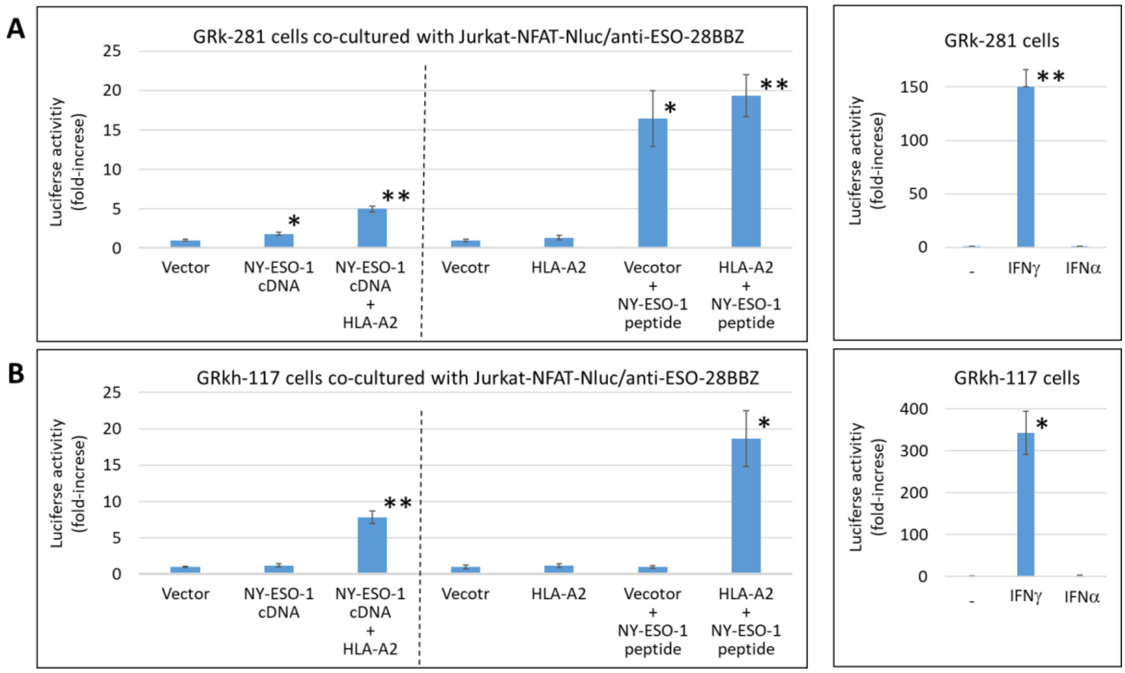

Supplementary Fig. 17. GRkh-117 cells can present specific antigens with the individual HLA type. We established a stable cell line by knockout of endogenous HLA-A, HLA-B, HLA-C, and IFNAR1 of GR-535 cells to present specific antigens with the individual HLA type. **A**. GRk-281 cells responded to the transfected NY-ESO-1 cDNA or NY-OSO-1 peptide load without exogenous HLA-A2 expression. **B**. The GRkh-117 cells did not respond to the transfected NY-ESO-1 cDNA or NY-OSO-1 peptide load due to a lack of endogenous HLAs against the T cells expressing anti-NY-ESO-1-scFv-28BBZ. However, the responses recovered by transfecting HLA-A2 at the same time. Both GRk-281 and GRkh-117 cells lack IFNα-responses because of endogenous IFNAR1 knockout (Right panels). Asterisks indicate a significant difference from the control (*p < 0.001, **p < 0.0001).

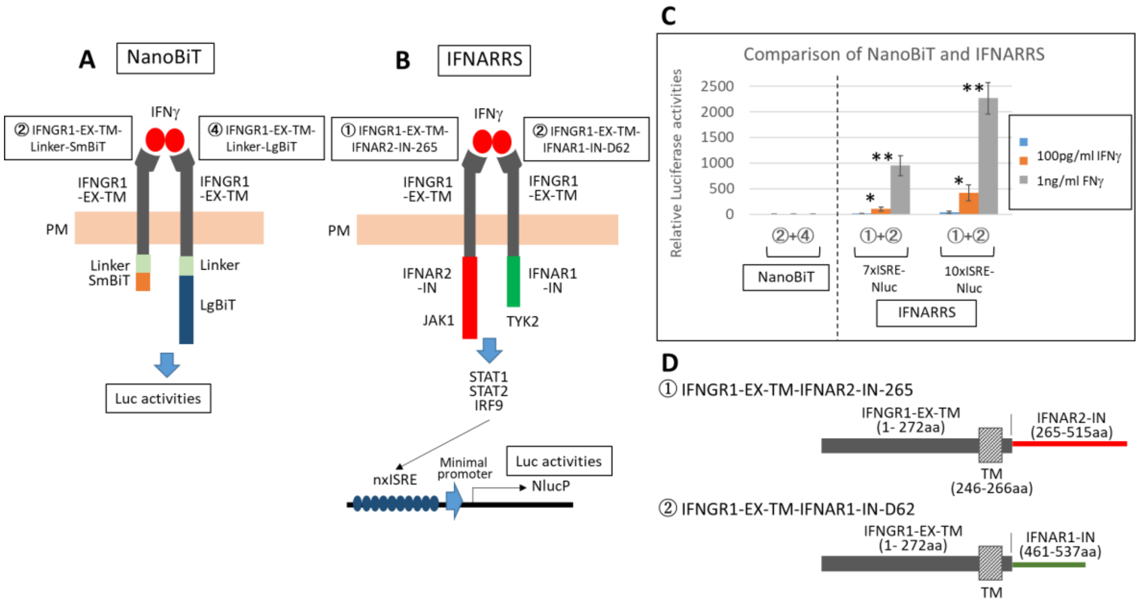

Supplementary Fig. 18. Application for the ligand-dependent clustering (IFNGR1-dimer formation). In the **IFNARRS (B)**, the extracellular and transmembrane regions of IFNGR1 (IFNGR1-EX-TM) were fused to the intracellular domains of IFNAR2 and IFNAR1 (IFNAR2-IN-265 and IFNAR1-IN-D62). The constructs are shown in **D.** Their DNA sequences are listed in Supplementary Table 13. Binding of dimeric IFNγ to the extracellular IFNGR1 brings the intracellular domains of IFNAR1 and IFNAR2 into proximity to send the JAK-STAT-dependent signal. In the **NanoBiT system (A)**, the same IFNGR1-EX-TM was fused to SmBiT and LgBiT via a short peptide linker (Linker), as depicted in Supplementary Fig. 19A. **C. Comparison of IFNRRS to NanoBiT.** HEK293T cells seeded in a 96-well plate were transfected with the indicated plasmids. After 24 h, the cells were incubated for 12 h with the indicated concentrations of IFNγ. The Nluc assay was performed with the same amount of substrate (mean ± sd of three independent experiments). IFNγ induced strong Nluc activities in IFNARRS but not in NanoBiT. Asterisks indicate significant difference compared with the control (*p<0.01, **p<0.0001). IFNGR1, IFNγ receptor 1.

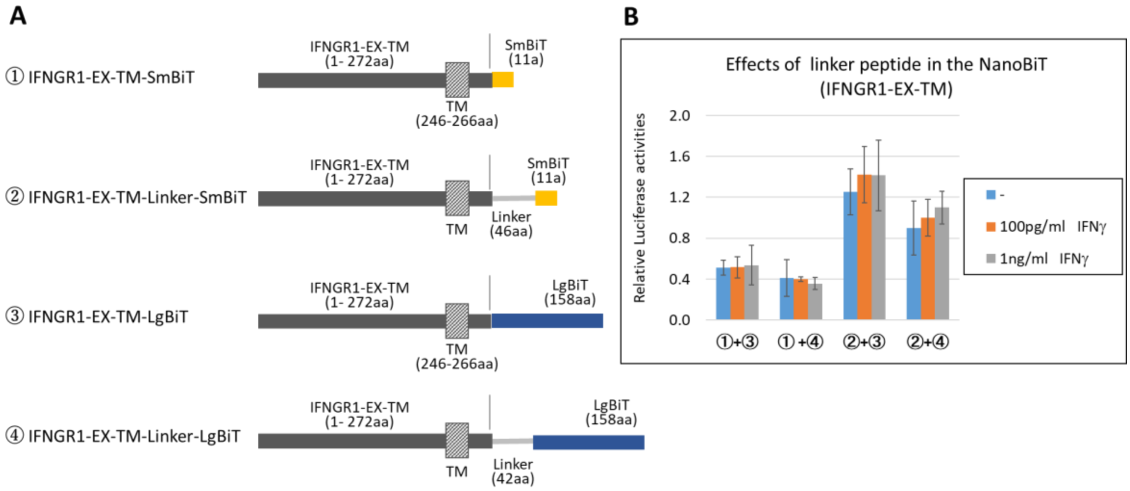

Supplementary Fig. 19. Application for the ligand-dependent clustering (IFNGR1-dimer formation). A. Constructs for NanoBiT; The same extracellular and transmembrane regions of IFNGR1 were fused to SmBiT and LgBiT with or without the linker peptide ([1], [2], [3], and [4]). Their DNA sequences are listed in Supplementary Table 14. **B. NanoBiT;** The indicated pairs of plasmids were transfected, and treated with the indicated concentration of IFNγ for 12h, after 24h of transfection. Any combinations of EX-IFNGR1-LgBiT and EX-IFNGR1-SmBiT with or without insertion of the linker peptide did not increase Nluc activities due to the addition of IFNγ.

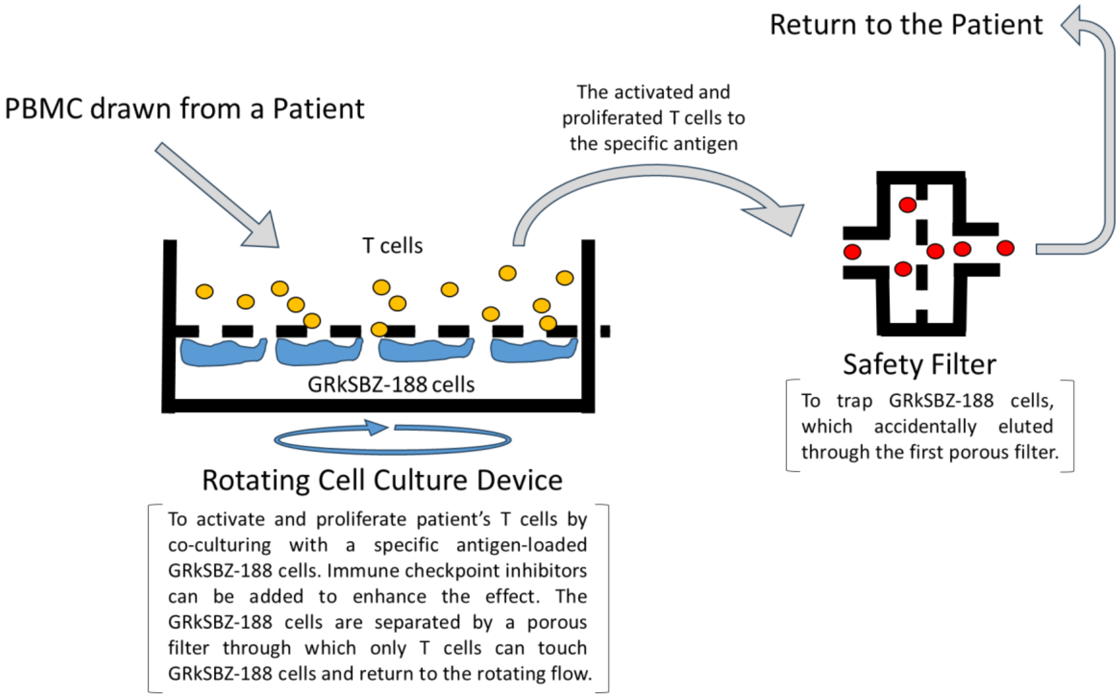

Supplementary Fig 20. Possible use of GRkSBZ-188 cells *ex vivo*. GRkSBZ-188 cells stably express IL-2, B7-1, and anti-CD3ε-scFv-TM in response to IFN-γ, and express constitutively ZZ-TM to recruit on the cell surface any antibodies via the Fc-region and can be used to activate and proliferate T cells to a specific antigen by co-culturing after loading with the specific antigen *ex vivo.* Recruiting immune checkpoint inhibitors, including anti-PD-1 antibodies on the cell surface, GRkSBZ-188 cells could prevent T cell exhaustion more effectively. As T cells are markedly smaller than HEK293T cells, the activated T cells could be separated using a porous filter through which only T cells can contact GRkSBZ-188 cells and return to the rotating flow. For safety, GRkSBZ-188 cells, which accidentally eluted through the first porous filter, could be trapped in another porous filter that allowed the passage of T cells but not GRkSBZ-188 cells.

Supplementary Table Captions

Supplementary Table 1 contains the primer sequences for amplifying the respective genes. Supplementary Tables 2–14 contain DNA sequences of the plasmid constructs in Supplementary Figs. 1–19.

Supplementary Table 2: Supplementary Fig. 1.
Supplementary Table 3: Supplementary Fig. 2.
Supplementary Table 4: Supplementary Figs. 3 and 4.
Supplementary Table 5: Supplementary Fig. 5.
Supplementary Table 6: Supplementary Fig. 6.
Supplementary Table 7: Supplementary Fig. 8.
Supplementary Table 8: Supplementary Fig. 9.
Supplementary Table 9: Supplementary Fig. 10.
Supplementary Table 10: Supplementary Fig. 11.
Supplementary Table 11: Supplementary Fig. 12.
Supplementary Table 12: Supplementary Fig. 15.
Supplementary Table 13: Supplementary Fig.18.
Supplementary Table 14: Supplementary Fig.19.

Supplementary Table 1

Primer sequences for amplifying the respective genes.

Primers to clone cDNAs

		5'→3'
IFNAR1-TM-IN-394	Forward	cccctcgagTCCTAATTTGAAACCACTGACTGT
	Reverse	ccctctagagTCATACAAAGTCCTGCTGTAGTTCT
IFNAR2-TM-IN-231	Forward	cccctcgagtCCACCTGGCCAGGAATCAGAAT
	Reverse	cggtctagagTCATCTCATTATATAACCATCCCCAAGG
IFNGR1-EX3	Forward	cccaagcttgccaccATGGCTCTCCTCTTTCTCCTAC
	Reverse	ccgctcgagCCTTTTATACTGCTATTGAAAATGGTAATAC
IFNGR2-EX5	Forward	ggcaagcttgccaccATGCGACCGACGCTGCTGT
	Reverse	gcgctcgaGACTTGCTGAAGCTCAGTGGAGG
IFNG (full-length)	Forward	ccgAATTCTCTCGGAAACGATGAAATATAC
	Reverse	gggctcgAGGACAACCATTACTGGGATGCT
IL-2 (full-length)	Forward	cccggatccgccaccATGTACAGGATGCAACTCCTGTCT
	Reverse	cccgcggccgcTCAAGTCAGTGTTGAGATGATGCT
IL2RA (full-length)	Forward	gggaagcttgccaccATGGATTCATACCTGCTGATGTGG
	Reverse	ggcctcgagCTAGATTGTTCTTCTACTCTTCCTCTG
IL2RB-EX	Forward	gggaagcttgccaccATGGCGGCCCCTGCTCTGT
	Reverse	ggcgtcgacccGGTGTCCTTCCCAAGGGCTGCAG
IL2RG-EX	Forward	gggaagcttgccaccATGTTGAAGCCATCATTACCATTCACA
	Reverse	gggctcgagccGGCTTCCAATGCAAACAGGAAA
B7-1 (full-length)	Forward	gggaagcttgccaccATGGGCCACACACGGAGGCA
	Reverse	gcgtctagaTTATACAGGGCGTACACTTTCCCTTCTC
Anti-CD3ε-scFv-TM (0.35 kb fragment for RT-PCR)	Forward	ggcgctagcgagGTCCAGCTGCAGCAGTCTG
	Reverse	gggctcgagccTGAGGAGACTGTGAGAGTGG

Supplementary Table 2

DNA sequences in the plasmid constructs of Supplementary Fig 1A.

① HER2-EX-IFNAR1-TM-IN-394

ATGGAGCTGGCGGCCTTGTGCCGCTGGGGGCTCCTCCTCGCCCTCTTGCCCCCCGG
AGCCGCGAGCACCCAAGTGTGCACCGGCACAGACATGAAGCTGCGGCTCCCTGCCA
GTCCCGAGACCCACCTGGACATGCTCCGCCACCTCTACCAGGGCTGCCAGGTGGTG
CAGGGAAACCTGGAACTCACCTACCTGCCCACCAATGCCAGCCTGTCCTTCCTGCAG
GATATCCAGGAGGTGCAGGGCTACGTGCTCATCGCTCACAACCAAGTGAGGCAGGT
CCCACTGCAGAGGCTGCGGATTGTGCGAGGCACCCAGCTCTTTGAGGACAACTATGC
CCTGGCCGTGCTAGACAATGGAGACCCGCTGAACAATACCACCCCTGTCACAGGGGC
CTCCCCAGGAGGCCTGCGGGAGCTGCAGCTTCGAAGCCTCACAGAGATCTTGAAAG
GAGGGGTCTTGATCCAGCGGAACCCCCAGCTCTGCTACCAGGACACGATTTTGTGGA
AGGACATCTTCCACAAGAACAACCAGCTGGCTCTCACACTGATAGACACCAACCGCT
CTCGGGCCTGCCACCCCTGTTCTCCGATGTGTAAGGGCTCCCGCTGCTGGGGAGAG
AGTTCTGAGGATTGTCAGAGCCTGACGCGCACTGTCTGTGCCGGTGGCTGTGCCCG
CTGCAAGGGGCCACTGCCCACTGACTGCTGCCATGAGCAGTGTGCTGCCGGCTGCA
CGGGCCCCAAGCACTCTGACTGCCTGGCCTGCCTCCACTTCAACCACAGTGGCATCT
GTGAGCTGCACTGCCCAGCCCTGGTCACCTACAACACAGACACGTTTGAGTCCATGC
CCAATCCCGAGGGCCGGTATACATTCGGCGCCAGCTGTGTGACTGCCTGTCCCTACA
ACTACCTTTCTACGGACGTGGGATCCTGCACCCTCGTCTGCCCCCTGCACAACCAAG
AGGTGACAGCAGAGGATGGAACACAGCGGTGTGAGAAGTGCAGCAAGCCCTGTGCC
CGAGTGTGCTATGGTCTGGGCATGGAGCACTTGCGAGAGGTGAGGGCAGTTACCAG
TGCCAATATCCAGGAGTTTGCTGGCTGCAAGAAGATCTTTGGGAGCCTGGCATTTCT
GCCGGAGAGCTTTGATGGGGACCCAGCCTCCAACACTGCCCCGCTCCAGCCAGAGC
AGCTCCAAGTGTTTGAGACTCTGGAAGAGATCACAGGTTACCTATACATCTCAGCAT
GGCCGGACAGCCTGCCTGACCTCAGCGTCTTCCAGAACCTGCAAGTAATCCGGGGA
CGAATTCTGCACAATGGCGCCTACTCGCTGACCCTGCAAGGGCTGGGCATCAGCTG
GCTGGGGCTGCGCTCACTGAGGGAACTGGGCAGTGGACTGGCCCTCATCCACCATA
ACACCCACCTCTGCTTCGTGCACACGGTGCCCTGGGACCAGCTCTTTCGGAACCCGC
ACCAAGCTCTGCTCCACACTGCCAACCGGCCAGAGGACGAGTGTGTGGGCGAGGGC
CTGGCCTGCCACCAGCTGTGCGCCCGAGGGCACTGCTGGGGTCCAGGGCCCACCCA
GTGTGTCAACTGCAGCCAGTTCCTTCGGGGCCAGGAGTGCGTGGAGGAATGCCGAG
TACTGCAGGGGCTCCCCAGGGAGTATGTGAATGCCAGGCACTGTTTGCCGTGCCAC
CCTGAGTGTCAGCCCCAGAATGGCTCAGTGACCTGTTTTGGACCGGAGGCTGACCA
GTGTGTGGCCTGTGCCCACTATAAGGACCCTCCCTTCTGCGTGGCCCGCTGCCCCA
GCGGTGTGAAACCTGACCTCTCCTACATGCCCATCTGGAAGTTTCCAGATGAGGAG
GCGCATGCCAGCCTTGCCCCATCAACTGCACCCACTCCTGTGTGGACCTGGATGACA
AGGGCTGCCCCGCCGAGCAGAGAGCCAGCCCTggcggccgctcgagtCCTAATTTGAAAC
CACTGACTGTATATTGTGTGAAAGCCAGAGCACACACCATGGATGAAAAGCTGAATA
AAAGCAGTGTTTTTAGTGACGCTGTATGTGAGAAAACAAAACCAGGAAATACCTCTAA
AATTTGGCTTATAGTTGGAATTTGTATTGCATTATTTGCTCTCCCGTTTGTCATTTAT
GCTGCGAAAGTCTTCTTGAGATGCATCAATTATGTCTTCTTTCCATCACTTAAACCTT
CTTCCAGTATAGATGAGTATTTCTCTGAACAGCCATTGAAGAATCTTCTGCTTTCAAC
TTCTGAGGAACAAATCGAAAAATGTTTCATAATTGAAAATATAAGCACAATTGCTACA
GTAGAAGAAACTAATCAAACTGATGAAGATCATAAAAAATACAGTTCCCAAACTAGCC
AAGATTCAGGAAATTATTCTAATGAAGATGAAAGCGAAAGTAAAACAAGTGAAGAACT
ACAGCAGGACTTTGTATGA

② HER2-EX-IFNAR2-TM-IN-231

ATGGAGCTGGCGGCCTTGTGCCGCTGGGGGCTCCTCCTCGCCCTCTTGCCCCCCGG
AGCCGCGAGCACCCAAGTGTGCACCGGCACAGACATGAAGCTGCGGCTCCCTGCCA
GTCCCGAGACCCACCTGGACATGCTCCGCCACCTCTACCAGGGCTGCCAGGTGGTG
CAGGGAAACCTGGAACTCACCTACCTGCCCACCAATGCCAGCCTGTCCTTCCTGCAG

GATATCCAGGAGGTGCAGGGCTACGTGCTCATCGCTCACAACCAAGTGAGGCAGGT
CCCACTGCAGAGGCTGCGGATTGTGCGAGGCACCCAGCTCTTTGAGGACAACTATGC
CCTGGCCGTGCTAGACAATGGAGACCCGCTGAACAATACCACCCCTGTCACAGGGGC
CTCCCCAGGAGGCCTGCGGGAGCTGCAGCTTCGAAGCCTCACAGAGATCTTGAAAG
GAGGGGTCTTGATCCAGCGGAACCCCCAGCTCTGCTACCAGGACACGATTTTGTGGA
AGGACATCTTCCACAAGAACAACCAGCTGGCTCTCACACTGATAGACACCAACCGCT
CTCGGGCCTGCCACCCCTGTTCTCCGATGTGTAAGGGCTCCCGCTGCTGGGGAGAG
AGTTCTGAGGATTGTCAGAGCCTGACGCGCACTGTCTGTGCCGGTGGCTGTGCCCG
CTGCAAGGGGCCACTGCCCACTGACTGCTGCCATGAGCAGTGTGCTGCCGGCTGCA
CGGGCCCCAAGCACTCTGACTGCCTGGCCTGCCTCCACTTCAACCACAGTGGCATCT
GTGAGCTGCACTGCCCAGCCCTGGTCACCTACAACACAGACACGTTTGAGTCCATGC
CCAATCCCGAGGGCCGGTATACATTCGGCGCCAGCTGTGTGACTGCCTGTCCCTACA
ACTACCTTTCTACGGACGTGGGATCCTGCACCCTCGTCTGCCCCCTGCACAACCAAG
AGGTGACAGCAGAGGATGGAACACAGCGGTGTGAGAAGTGCAGCAAGCCCTGTGCC
CGAGTGTGCTATGGTCTGGGCATGGAGCACTTGCGAGAGGTGAGGGCAGTTACCAG
TGCCAATATCCAGGAGTTTGCTGGCTGCAAGAAGATCTTTGGGAGCCTGGCATTTCT
GCCGGAGAGCTTTGATGGGGACCCAGCCTCCAACACTGCCCCGCTCCAGCCAGAGC
AGCTCCAAGTGTTTGAGACTCTGGAAGAGATCACAGGTTACCTATACATCTCAGCAT
GGCCGGACAGCCTGCCTGACCTCAGCGTCTTCCAGAACCTGCAAGTAATCCGGGGA
CGAATTCTGCACAATGGCGCCTACTCGCTGACCCTGCAAGGGCTGGGCATCAGCTG
GCTGGGGCTGCGCTCACTGAGGGAACTGGGCAGTGGACTGGCCCTCATCCACCATA
ACACCCACCTCTGCTTCGTGCACACGGTGCCCTGGGACCAGCTCTTTCGGAACCCGC
ACCAAGCTCTGCTCCACACTGCCAACCGGCCAGAGGACGAGTGTGTGGGCGAGGGC
CTGGCCTGCCACCAGCTGTGCGCCCGAGGGCACTGCTGGGGTCCAGGGCCCACCCA
GTGTGTCAACTGCAGCCAGTTCCTTCGGGGCCAGGAGTGCGTGGAGGAATGCCGAG
TACTGCAGGGGCTCCCCAGGGAGTATGTGAATGCCAGGCACTGTTTGCCGTGCCAC
CCTGAGTGTCAGCCCCAGAATGGCTCAGTGACCTGTTTTGGACCGGAGGCTGACCA
GTGTGTGGCCTGTGCCCACTATAAGGACCCTCCCTTCTGCGTGGCCCGCTGCCCCA
GCGGTGTGAAACCTGACCTCTCCTACATGCCCATCTGGAAGTTTCCAGATGAGGAG
GCGCATGCCAGCCTTGCCCCATCAACTGCACCCACTCCTGTGTGGACCTGGATGACA
AGGGCTGCCCCGCCGAGCAGAGAGCCAGCCCTggcggccgctcgagtCCACCTGGCCAGG
AATCAGAATCAGCAGAATCTGCCAAAATAGGAGGAATAATTACTGTGTTTTTGATAGC
ATTGGTCTTGACAAGCACCATAGTGACACTGAAATGGATTGGTTATATATGCTTAAGA
AATAGCCTCCCCAAAGTCTTGAATTTTCATAACTTTTTAGCCTGGCCATTTCCTAACC
TGCCACCGTTGGAAGCCATGGATATGGTGGAGGTCATTTACATCAACAGAAAGAAGA
AAGTGTGGGATTATAATTATGATGATGAAAGTGATAGCGATACTGAGGCAGCGCCCA
GGACAAGTGGCGGTGGCTATACCATGCATGGACTGACTGTCAGGCCTCTGGGTCAG
GCCTCTGCCACCTCTACAGAATCCCAGTTGATAGACCCGGAGTCCGAGGAGGAGCCT
GACCTGCCTGAGGTTGATGTGGAGCTCCCCACGATGCCAAAGGACAGCCCTCAGCA
GTTGGAACTCTTGAGTGGGCCCTGTGAGAGGAGAAAGAGTCCACTCCAGGACCCTTT
TCCCGAAGAGGACTACAGCTCCACGGAGGGGTCTGGGGGCAGAATTACCTTCAATGT
GGACTTAAACTCTGTGTTTTTGAGAGTTCTTGATGACGAGGACAGTGACGACTTAGA
AGCCCCTCTGATGCTATCGTCTCATCTGGAAGAGATGGTTGACCCAGAGGATCCTGA
TAATGTGCAATCAAACCATTTGCTGGCCAGCGGGGAAGGGACACAGCCAACCTTTCC
CAGCCCCTCTTCAGAGGGCCTGTGGTCCGAAGATGCTCCATCTGATCAAAGTGACAC
TTCTGAGTCAGATGTTGACCTTGGGGATGGTTATATAATGAGATGA

③　IL6SP-anti-HER2-Af-IFNAR1-TM-IN-394

ATGAACTCCTTCTCCACAAGCGCCTTCGGTCCAGTTGCCTTCTCCCTGGGCCTGCTC
CTGGTGTTGCCTGCTGCCTTCCCTGCCCCAgtcccctccggaggtggagggtcgtcaggtGTGG
ACAATAAATTCAACAAAGAAATGAGAAACGCCTACTGGGAGATCGCCCTGCTACCCA
ACCTGAACAACCAACAGAAGAGAGCGTTTATCAGAAGCCTGTACGACGACCCTAGCC
AATCTGCTAACCTGCTGGCAGAGGCTAAGAAACTGAATGACGCCCAAGCCCCTAAGg
gcggtggaggctcgagtCCTAATTTGAAACCACTGACTGTATATTGTGTGAAAGCCAGAGCA
CACACCATGGATGAAAAGCTGAATAAAAGCAGTGTTTTTAGTGACGCTGTATGTGAG
AAAACAAAACCAGGAAATACCTCTAAAATTTGGCTTATAGTTGGAATTTGTATTGCAT

TATTTGCTCTCCCGTTTGTCATTTATGCTGCGAAAGTCTTCTTGAGATGCATCAATTA
TGTCTTCTTTCCATCACTTAAACCTTCTTCCAGTATAGATGAGTATTTCTCTGAACAG
CCATTGAAGAATCTTCTGCTTTCAACTTCTGAGGAACAAATCGAAAAATGTTTCATAA
TTGAAAATATAAGCACAATTGCTACAGTAGAAGAAACTAATCAAACTGATGAAGATCA
TAAAAAATACAGTTCCCAAACTAGCCAAGATTCAGGAAATTATTCTAATGAAGATGAA
AGCGAAAGTAAAACAAGTGAAGAACTACAGCAGGACTTTGTATGA

④ anti-HER2-Af-IFNAR2-TM-IN-231

ATGAACTCCTTCTCCACAAGCGCCTTCGGTCCAGTTGCCTTCTCCCTGGGCCTGCTC
CTGGTGTTGCCTGCTGCCTTCCCTGCCCCAgtcccctccggaggtggagggtcgtcaggtGTGG
ACAATAAATTCAACAAAGAAATGAGAAACGCCTACTGGGAGATCGCCCTGCTACCCA
ACCTGAACAACCAACAGAAGAGAGCGTTTATCAGAAGCCTGTACGACGACCCTAGCC
AATCTGCTAACCTGCTGGCAGAGGCTAAGAAACTGAATGACGCCCAAGCCCCTAAGg
gcggtggaggctcgagtCCACCTGGCCAGGAATCAGAATCAGCAGAATCTGCCAAAATAGG
AGGAATAATTACTGTGTTTTTGATAGCATTGGTCTTGACAAGCACCATAGTGACACTG
AAATGGATTGGTTATATATGCTTAAGAAATAGCCTCCCCAAAGTCTTGAATTTTCATA
ACTTTTTAGCCTGGCCATTTCCTAACCTGCCACCGTTGGAAGCCATGGATATGGTGG
AGGTCATTTACATCAACAGAAAGAAGAAAGTGTGGGATTATAATTATGATGATGAAAG
TGATAGCGATACTGAGGCAGCGCCCAGGACAAGTGGCGGTGGCTATACCATGCATG
GACTGACTGTCAGGCCTCTGGGTCAGGCCTCTGCCACCTCTACAGAATCCCAGTTGA
TAGACCCGGAGTCCGAGGAGGAGCCTGACCTGCCTGAGGTTGATGTGGAGCTCCCC
ACGATGCCAAAGGACAGCCCTCAGCAGTTGGAACTCTTGAGTGGGCCCTGTGAGAG
GAGAAAGAGTCCACTCCAGGACCCTTTTCCCGAAGAGGACTACAGCTCCACGGAGGG
GTCTGGGGGCAGAATTACCTTCAATGTGGACTTAAACTCTGTGTTTTTGAGAGTTCTT
GATGACGAGGACAGTGACGACTTAGAAGCCCCTCTGATGCTATCGTCTCATCTGGAA
GAGATGGTTGACCCAGAGGATCCTGATAATGTGCAATCAAACCATTTGCTGGCCAGC
GGGGAAGGGACACAGCCAACCTTTCCCAGCCCCTCTTCAGAGGGCCTGTGGTCCGA
AGATGCTCCATCTGATCAAAGTGACACTTCTGAGTCAGATGTTGACCTTGGGGATGG
TTATATAATGAGATGA

DNA sequences in the plasmid constructs of Supplementary Fig 1B.

4xISRE-NlucP

GGGAAAGTGAAACTAGGGAAAGTGAAACTAGGGAAAGTGAAACTAGGGAAAGTGAAA
CTActcgaggaattcaagcttagacactAGAGGGTATATAATGGAAGCTCGACTTCCAGcttggca
atccggtactgttggtaaagccaccATGGTCTTCACACTCGAAGATTTCGTTGGGGACTGGCGA
CAGACAGCCGGCTACAACCTGGACCAAGTCCTTGAACAGGGAGGTGTGTCCAGTTTG
TTTCAGAATCTCGGGGTGTCCGTAACTCCGATCCAAAGGATTGTCCTGAGCGGTGAA
AATGGGCTGAAGATCGACATCCATGTCATCATCCCGTATGAAGGTCTGAGCGGCGAC
CAAATGGGCCAGATCGAAAAAATTTTTAAGGTGGTGTACCCTGTGGATGATCATCAC
TTTAAGGTGATCCTGCACTATGGCACACTGGTAATCGACGGGGTTACGCCGAACATG
ATCGACTATTTCGGACGGCCGTATGAAGGCATCGCCGTGTTCGACGGCAAAAAGATC
ACTGTAACAGGGACCCTGTGGAACGGCAACAAAATTATCGACGAGCGCCTGATCAAC
CCCGACGGCTCCCTGCTGTTCCGAGTAACCATCAACGGAGTGACCGGCTGGCGGCT
GTGCGAACGCATTCTGGCGAATTCTCACGGCTTTCCGCCTGAGGTTGAAGAGCAAGC
CGCCGGTACATTGCCTATGTCCTGCGCACAAGAAAGCGGTATGGACCGGCACCCAG
CCGCTTGTGCTTCAGCTCGCATCAACGTCTAA

6xISRE-NlucP

GGGAAAGTGAAACTAGGGAAAGTGAAACTAGGGAAAGTGAAACTAcagcttGGGAAAG
TGAAACTAGGGAAAGTGAAACTAGGGAAAGTGAAACTAcagcttagacactAGAGGGTAT
ATAATGGAAGCTCGACTTCCAGcttggcaatccggtactgttggtaaagccaccATGGTCTTCACA
CTCGAAGATTTCGTTGGGGACTGGCGACAGACAGCCGGCTACAACCTGGACCAAGTC
CTTGAACAGGGAGGTGTGTCCAGTTTGTTTCAGAATCTCGGGGTGTCCGTAACTCCG
ATCCAAAGGATTGTCCTGAGCGGTGAAAATGGGCTGAAGATCGACATCCATGTCATC
ATCCCGTATGAAGGTCTGAGCGGCGACCAAATGGGCCAGATCGAAAAAATTTTTAAG
GTGGTGTACCCTGTGGATGATCATCACTTTAAGGTGATCCTGCACTATGGCACACTG
GTAATCGACGGGGTTACGCCGAACATGATCGACTATTTCGGACGGCCGTATGAAGGC
ATCGCCGTGTTCGACGGCAAAAAGATCACTGTAACAGGGACCCTGTGGAACGGCAAC
AAAATTATCGACGAGCGCCTGATCAACCCCGACGGCTCCCTGCTGTTCCGAGTAACC
ATCAACGGAGTGACCGGCTGGCGGCTGTGCGAACGCATTCTGGCGAATTCTCACGG
CTTTCCGCCTGAGGTTGAAGAGCAAGCCGCCGGTACATTGCCTATGTCCTGCGCACA
AGAAAGCGGTATGGACCGGCACCCAGCCGCTTGTGCTTCAGCTCGCATCAACGTCTA
A

7xISRE-NlucP

GGGAAAGTGAAACTAGGGAAAGTGAAACTAGGGAAAGTGAAACTAGGGAAAGTGAAA
CTActcgaggaattcaagcttGGGAAAGTGAAACTAGGGAAAGTGAAACTAGGGAAAGTGAA
ACTAcagcttagacactAGAGGGTATATAATGGAAGCTCGACTTCCAGcttggcaatccggtactg
ttggtaaagccaccATGGTCTTCACACTCGAAGATTTCGTTGGGGACTGGCGACAGACAG
CCGGCTACAACCTGGACCAAGTCCTTGAACAGGGAGGTGTGTCCAGTTTGTTTCAGA
ATCTCGGGGTGTCCGTAACTCCGATCCAAAGGATTGTCCTGAGCGGTGAAAATGGGC
TGAAGATCGACATCCATGTCATCATCCCGTATGAAGGTCTGAGCGGCGACCAAATGG
GCCAGATCGAAAAAATTTTTAAGGTGGTGTACCCTGTGGATGATCATCACTTTAAGG
TGATCCTGCACTATGGCACACTGGTAATCGACGGGGTTACGCCGAACATGATCGACT
ATTTCGGACGGCCGTATGAAGGCATCGCCGTGTTCGACGGCAAAAAGATCACTGTAA
CAGGGACCCTGTGGAACGGCAACAAAATTATCGACGAGCGCCTGATCAACCCCGACG
GCTCCCTGCTGTTCCGAGTAACCATCAACGGAGTGACCGGCTGGCGGCTGTGCGAA
CGCATTCTGGCGAATTCTCACGGCTTTCCGCCTGAGGTTGAAGAGCAAGCCGCCGGT
ACATTGCCTATGTCCTGCGCACAAGAAAGCGGTATGGACCGGCACCCAGCCGCTTGT
GCTTCAGCTCGCATCAACGTCTAA

10xISRE-NlucP

GGGAAAGTGAAACTAGGGAAAGTGAAACTAGGGAAAGTGAAACTAGGGAAAGTGAAA
CTActcgaggaattcaagcttGGGAAAGTGAAACTAGGGAAAGTGAAACTAGGGAAAGTGAA
ACTAcagcttGGGAAAGTGAAACTAGGGAAAGTGAAACTAGGGAAAGTGAAACTAcagctt
agacactAGAGGGTATATAATGGAAGCTCGACTTCCAGcttggcaatccggtactgttggtaaagcc
accATGGTCTTCACACTCGAAGATTTCGTTGGGGACTGGCGACAGACAGCCGGCTAC
AACCTGGACCAAGTCCTTGAACAGGGAGGTGTGTCCAGTTTGTTTCAGAATCTCGGG
GTGTCCGTAACTCCGATCCAAAGGATTGTCCTGAGCGGTGAAAATGGGCTGAAGATC
GACATCCATGTCATCATCCCGTATGAAGGTCTGAGCGGCGACCAAATGGGCCAGATC
GAAAAAATTTTTAAGGTGGTGTACCCTGTGGATGATCATCACTTTAAGGTGATCCTGC
ACTATGGCACACTGGTAATCGACGGGGTTACGCCGAACATGATCGACTATTTCGGAC
GGCCGTATGAAGGCATCGCCGTGTTCGACGGCAAAAAGATCACTGTAACAGGGACCC
TGTGGAACGGCAACAAAATTATCGACGAGCGCCTGATCAACCCCGACGGCTCCCTGC
TGTTCCGAGTAACCATCAACGGAGTGACCGGCTGGCGGCTGTGCGAACGCATTCTG
GCGAATTCTCACGGCTTTCCGCCTGAGGTTGAAGAGCAAGCCGCCGGTACATTGCCT

ATGTCCTGCGCACAAGAAAGCGGTATGGACCGGCACCCAGCCGCTTGTGCTTCAGCT
CGCATCAACGTCTAA

15xISRE-NlucP

GGGAAAGTGAAACTAGGGAAAGTGAAACTAGGGAAAGTGAAACTAcagcttGGGAAAG
TGAAACTAGGGAAAGTGAAACTAGGGAAAGTGAAACTAcagcttGGGAAAGTGAAACTA
GGGAAAGTGAAACTAGGGAAAGTGAAACTAcagcttGGGAAAGTGAAACTAGGGAAAG
TGAAACTAGGGAAAGTGAAACTAcagcttGGGAAAGTGAAACTAGGGAAAGTGAAACTA
GGGAAAGTGAAACTAcagcttagacactAGAGGGTATATAATGGAAGCTCGACTTCCAGctt
ggcaatccggtactgttggtaaagccaccATGGTCTTCACACTCGAAGATTTCGTTGGGGACTG
GCGACAGACAGCCGGCTACAACCTGGACCAAGTCCTTGAACAGGGAGGTGTGTCCA
GTTTGTTTCAGAATCTCGGGGTGTCCGTAACTCCGATCCAAAGGATTGTCCTGAGCG
GTGAAAATGGGCTGAAGATCGACATCCATGTCATCATCCCGTATGAAGGTCTGAGCG
GCGACCAAATGGGCCAGATCGAAAAAATTTTTAAGGTGGTGTACCCTGTGGATGATC
ATCACTTTAAGGTGATCCTGCACTATGGCACACTGGTAATCGACGGGGTTACGCCGA
ACATGATCGACTATTTCGGACGGCCGTATGAAGGCATCGCCGTGTTCGACGGCAAAA
AGATCACTGTAACAGGGACCCTGTGGAACGGCAACAAAATTATCGACGAGCGCCTGA
TCAACCCCGACGGCTCCCTGCTGTTCCGAGTAACCATCAACGGAGTGACCGGCTGGC
GGCTGTGCGAACGCATTCTGGCGAATTCTCACGGCTTTCCGCCTGAGGTTGAAGAGC
AAGCCGCCGGTACATTGCCTATGTCCTGCGCACAAGAAAGCGGTATGGACCGGCACC
CAGCCGCTTGTGCTTCAGCTCGCATCAACGTCTAA

Supplementary Table 3

DNA sequences in the plasmid constructs of Supplementary Fig 2.

① HER2-EX-SmBiT

ATGGAGCTGGCGGCCTTGTGCCGCTGGGGGCTCCTCCTCGCCCTCTTGCCCCCCGG
AGCCGCGAGCACCCAAGTGTGCACCGGCACAGACATGAAGCTGCGGCTCCCTGCCA
GTCCCGAGACCCACCTGGACATGCTCCGCCACCTCTACCAGGGCTGCCAGGTGGTG
CAGGGAAACCTGGAACTCACCTACCTGCCCACCAATGCCAGCCTGTCCTTCCTGCAG
GATATCCAGGAGGTGCAGGGCTACGTGCTCATCGCTCACAACCAAGTGAGGCAGGT
CCCACTGCAGAGGCTGCGGATTGTGCGAGGCACCCAGCTCTTTGAGGACAACTATGC
CCTGGCCGTGCTAGACAATGGAGACCCGCTGAACAATACCACCCCTGTCACAGGGGC
CTCCCCAGGAGGCCTGCGGGAGCTGCAGCTTCGAAGCCTCACAGAGATCTTGAAAG
GAGGGGTCTTGATCCAGCGGAACCCCCAGCTCTGCTACCAGGACACGATTTTGTGGA
AGGACATCTTCCACAAGAACAACCAGCTGGCTCTCACACTGATAGACACCAACCGCT
CTCGGGCCTGCCACCCCTGTTCTCCGATGTGTAAGGGCTCCCGCTGCTGGGGAGAG
AGTTCTGAGGATTGTCAGAGCCTGACGCGCACTGTCTGTGCCGGTGGCTGTGCCCG
CTGCAAGGGGCCACTGCCCACTGACTGCTGCCATGAGCAGTGTGCTGCCGGCTGCA
CGGGCCCCAAGCACTCTGACTGCCTGGCCTGCCTCCACTTCAACCACAGTGGCATCT
GTGAGCTGCACTGCCCAGCCCTGGTCACCTACAACACAGACACGTTTGAGTCCATGC
CCAATCCCGAGGGCCGGTATACATTCGGCGCCAGCTGTGTGACTGCCTGTCCCTACA
ACTACCTTTCTACGGACGTGGGATCCTGCACCCTCGTCTGCCCCCTGCACAACCAAG
AGGTGACAGCAGAGGATGGAACACAGCGGTGTGAGAAGTGCAGCAAGCCCTGTGCC
CGAGTGTGCTATGGTCTGGGCATGGAGCACTTGCGAGAGGTGAGGGCAGTTACCAG
TGCCAATATCCAGGAGTTTGCTGGCTGCAAGAAGATCTTTGGGAGCCTGGCATTTCT
GCCGGAGAGCTTTGATGGGGACCCAGCCTCCAACACTGCCCCGCTCCAGCCAGAGC
AGCTCCAAGTGTTTGAGACTCTGGAAGAGATCACAGGTTACCTATACATCTCAGCAT
GGCCGGACAGCCTGCCTGACCTCAGCGTCTTCCAGAACCTGCAAGTAATCCGGGGA
CGAATTCTGCACAATGGCGCCTACTCGCTGACCCTGCAAGGGCTGGGCATCAGCTG
GCTGGGGCTGCGCTCACTGAGGGAACTGGGCAGTGGACTGGCCCTCATCCACCATA

ACACCCACCTCTGCTTCGTGCACACGGTGCCCTGGGACCAGCTCTTTCGGAACCCGC
ACCAAGCTCTGCTCCACACTGCCAACCGGCCAGAGGACGAGTGTGTGGGCGAGGGC
CTGGCCTGCCACCAGCTGTGCGCCCGAGGGCACTGCTGGGGTCCAGGGCCCACCCA
GTGTGTCAACTGCAGCCAGTTCCTTCGGGGCCAGGAGTGCGTGGAGGAATGCCGAG
TACTGCAGGGGCTCCCCAGGGAGTATGTGAATGCCAGGCACTGTTTGCCGTGCCAC
CCTGAGTGTCAGCCCCAGAATGGCTCAGTGACCTGTTTTGGACCGGAGGCTGACCA
GTGTGTGGCCTGTGCCCACTATAAGGACCCTCCCTTCTGCGTGGCCCGCTGCCCCA
GCGGTGTGAAACCTGACCTCTCCTACATGCCCATCTGGAAGTTTCCAGATGAGGAGG
GCGCATGCCAGCCTTGCCCCATCAACTGCACCCACTCCTGTGTGGACCTGGATGACA
AGGGCTGCCCCGCCGAGCAGAGAGCCAGCCCTggcggccgctcgagcggtGTGACCGGCT
ACCGGCTGTTCGAGGAGATTCTGtaa

② HER2-EX-Linker-SmBiT

ATGGAGCTGGCGGCCTTGTGCCGCTGGGGGCTCCTCCTCGCCCTCTTGCCCCCCGG
AGCCGCGAGCACCCAAGTGTGCACCGGCACAGACATGAAGCTGCGGCTCCCTGCCA
GTCCCGAGACCCACCTGGACATGCTCCGCCACCTCTACCAGGGCTGCCAGGTGGTG
CAGGGAAACCTGGAACTCACCTACCTGCCCACCAATGCCAGCCTGTCCTTCCTGCAG
GATATCCAGGAGGTGCAGGGCTACGTGCTCATCGCTCACAACCAAGTGAGGCAGGT
CCCACTGCAGAGGCTGCGGATTGTGCGAGGCACCCAGCTCTTTGAGGACAACTATGC
CCTGGCCGTGCTAGACAATGGAGACCCGCTGAACAATACCACCCCTGTCACAGGGGC
CTCCCCAGGAGGCCTGCGGGAGCTGCAGCTTCGAAGCCTCACAGAGATCTTGAAAG
GAGGGGTCTTGATCCAGCGGAACCCCCAGCTCTGCTACCAGGACACGATTTTGTGGA
AGGACATCTTCCACAAGAACAACCAGCTGGCTCTCACACTGATAGACACCAACCGCT
CTCGGGCCTGCCACCCCTGTTCTCCGATGTGTAAGGGCTCCCGCTGCTGGGGAGAG
AGTTCTGAGGATTGTCAGAGCCTGACGCGCACTGTCTGTGCCGGTGGCTGTGCCCG
CTGCAAGGGGCCACTGCCCACTGACTGCTGCCATGAGCAGTGTGCTGCCGGCTGCA
CGGGCCCCAAGCACTCTGACTGCCTGGCCTGCCTCCACTTCAACCACAGTGGCATCT
GTGAGCTGCACTGCCCAGCCCTGGTCACCTACAACACAGACACGTTTGAGTCCATGC
CCAATCCCGAGGGCCGGTATACATTCGGCGCCAGCTGTGTGACTGCCTGTCCCTACA
ACTACCTTTCTACGGACGTGGGATCCTGCACCCTCGTCTGCCCCCTGCACAACCAAG
AGGTGACAGCAGAGGATGGAACACAGCGGTGTGAGAAGTGCAGCAAGCCCTGTGCC
CGAGTGTGCTATGGTCTGGGCATGGAGCACTTGCGAGAGGTGAGGGCAGTTACCAG
TGCCAATATCCAGGAGTTTGCTGGCTGCAAGAAGATCTTTGGGAGCCTGGCATTTCT
GCCGGAGAGCTTTGATGGGGACCCAGCCTCCAACACTGCCCCGCTCCAGCCAGAGC
AGCTCCAAGTGTTTGAGACTCTGGAAGAGATCACAGGTTACCTATACATCTCAGCAT
GGCCGGACAGCCTGCCTGACCTCAGCGTCTTCCAGAACCTGCAAGTAATCCGGGGA
CGAATTCTGCACAATGGCGCCTACTCGCTGACCCTGCAAGGGCTGGGCATCAGCTG
GCTGGGGCTGCGCTCACTGAGGGAACTGGGCAGTGGACTGGCCCTCATCCACCATA
ACACCCACCTCTGCTTCGTGCACACGGTGCCCTGGGACCAGCTCTTTCGGAACCCGC
ACCAAGCTCTGCTCCACACTGCCAACCGGCCAGAGGACGAGTGTGTGGGCGAGGGC
CTGGCCTGCCACCAGCTGTGCGCCCGAGGGCACTGCTGGGGTCCAGGGCCCACCCA
GTGTGTCAACTGCAGCCAGTTCCTTCGGGGCCAGGAGTGCGTGGAGGAATGCCGAG
TACTGCAGGGGCTCCCCAGGGAGTATGTGAATGCCAGGCACTGTTTGCCGTGCCAC
CCTGAGTGTCAGCCCCAGAATGGCTCAGTGACCTGTTTTGGACCGGAGGCTGACCA
GTGTGTGGCCTGTGCCCACTATAAGGACCCTCCCTTCTGCGTGGCCCGCTGCCCCA
GCGGTGTGAAACCTGACCTCTCCTACATGCCCATCTGGAAGTTTCCAGATGAGGAGG
GCGCATGCCAGCCTTGCCCCATCAACTGCACCCACTCCTGTGTGGACCTGGATGACA
AGGGCTGCCCCGCCGAGCAGAGAGCCAGCCCTggcggccgctcgagcGGTGGTGGAGGG
TCAGGAGGTGGAGGGtcgagcGGTGGTGGAGGGTCAGGAGGTGGAGGGtcgagcGGTG
GTGGAGGGTCAGGAGGTGGAGGGtcgagcgccATGGGCTCCGGAGGCGGATCCGGTG
GGtcgagcggtGTGACCGGCTACCGGCTGTTCGAGGAGATTCTGTAA

③ HER2-EX-LgBiT

ATGGAGCTGGCGGCCTTGTGCCGCTGGGGGCTCCTCCTCGCCCTCTTGCCCCCCGG
AGCCGCGAGCACCCAAGTGTGCACCGGCACAGACATGAAGCTGCGGCTCCCTGCCA
GTCCCGAGACCCACCTGGACATGCTCCGCCACCTCTACCAGGGCTGCCAGGTGGTG
CAGGGAAACCTGGAACTCACCTACCTGCCCACCAATGCCAGCCTGTCCTTCCTGCAG
GATATCCAGGAGGTGCAGGGCTACGTGCTCATCGCTCACAACCAAGTGAGGCAGGT
CCCACTGCAGAGGCTGCGGATTGTGCGAGGCACCCAGCTCTTTGAGGACAACTATGC
CCTGGCCGTGCTAGACAATGGAGACCCGCTGAACAATACCACCCCTGTCACAGGGGC
CTCCCCAGGAGGCCTGCGGGAGCTGCAGCTTCGAAGCCTCACAGAGATCTTGAAAG
GAGGGGTCTTGATCCAGCGGAACCCCCAGCTCTGCTACCAGGACACGATTTTGTGGA
AGGACATCTTCCACAAGAACAACCAGCTGGCTCTCACACTGATAGACACCAACCGCT
CTCGGGCCTGCCACCCCTGTTCTCCGATGTGTAAGGGCTCCCGCTGCTGGGGAGAG
AGTTCTGAGGATTGTCAGAGCCTGACGCGCACTGTCTGTGCCGGTGGCTGTGCCCG
CTGCAAGGGGCCACTGCCCACTGACTGCTGCCATGAGCAGTGTGCTGCCGGCTGCA
CGGGCCCCAAGCACTCTGACTGCCTGGCCTGCCTCCACTTCAACCACAGTGGCATCT
GTGAGCTGCACTGCCCAGCCCTGGTCACCTACAACACAGACACGTTTGAGTCCATGC
CCAATCCCGAGGGCCGGTATACATTCGGCGCCAGCTGTGTGACTGCCTGTCCCTACA
ACTACCTTTCTACGGACGTGGGATCCTGCACCCTCGTCTGCCCCCTGCACAACCAAG
AGGTGACAGCAGAGGATGGAACACAGCGGTGTGAGAAGTGCAGCAAGCCCTGTGCC
CGAGTGTGCTATGGTCTGGGCATGGAGCACTTGCGAGAGGTGAGGGCAGTTACCAG
TGCCAATATCCAGGAGTTTGCTGGCTGCAAGAAGATCTTTGGGAGCCTGGCATTTCT
GCCGGAGAGCTTTGATGGGGACCCAGCCTCCAACACTGCCCCGCTCCAGCCAGAGC
AGCTCCAAGTGTTTGAGACTCTGGAAGAGATCACAGGTTACCTATACATCTCAGCAT
GGCCGGACAGCCTGCCTGACCTCAGCGTCTTCCAGAACCTGCAAGTAATCCGGGGA
CGAATTCTGCACAATGGCGCCTACTCGCTGACCCTGCAAGGGCTGGGCATCAGCTG
GCTGGGGCTGCGCTCACTGAGGGAACTGGGCAGTGGACTGGCCCTCATCCACCATA
ACACCCACCTCTGCTTCGTGCACACGGTGCCCTGGGACCAGCTCTTTCGGAACCCGC
ACCAAGCTCTGCTCCACACTGCCAACCGGCCAGAGGACGAGTGTGTGGGCGAGGGC
CTGGCCTGCCACCAGCTGTGCGCCCGAGGGCACTGCTGGGGTCCAGGGCCCACCCA
GTGTGTCAACTGCAGCCAGTTCCTTCGGGGCCAGGAGTGCGTGGAGGAATGCCGAG
TACTGCAGGGGCTCCCCAGGGAGTATGTGAATGCCAGGCACTGTTTGCCGTGCCAC
CCTGAGTGTCAGCCCCAGAATGGCTCAGTGACCTGTTTTGGACCGGAGGCTGACCA
GTGTGTGGCCTGTGCCCACTATAAGGACCCTCCCTTCTGCGTGGCCCGCTGCCCCA
GCGGTGTGAAACCTGACCTCTCCTACATGCCCATCTGGAAGTTTCCAGATGAGGAG
GCGCATGCCAGCCTTGCCCCATCAACTGCACCCACTCCTGTGTGGACCTGGATGACA
AGGGCTGCCCCGCCGAGCAGAGAGCCAGCCCTggcggccgctcgagcggcGTCTTCACAC
TCGAAGATTTCGTTGGGGACTGGGAACAGACAGCCGCCTACAACCTGGACCAAGTCC
TTGAACAGGGAGGTGTGTCCAGTTTGCTGCAGAATCTCGCCGTGTCCGTAACTCCGA
TCCAAAGGATTGTCCGTAGCGGTGAAAATGCCCTGAAGATCGACATCCATGTCATCA
TCCCGTATGAAGGTCTGAGCGCCGACCAAATGGCCCAGATCGAAGAGGTGTTTAAGG
TGGTGTACCCTGTGGATGATCATCACTTTAAGGTGATCCTGCCCTATGGCACACTGG
TAATCGACGGGGTTACGCCGAACATGCTGAACTATTTCGGACGGCCGTATGAAGGCA
TCGCCGTGTTCGACGGCAAAAAGATCACTGTAACAGGGACCCTGTGGAACGGCAACA
AAATTATCGACGAGCGCCTGATCACCCCCGACGGCTCCATGCTGTTCCGAGTAACCA
TCAACAGTTAA

④ HER2-EX-Linker-LgBiT

ATGGAGCTGGCGGCCTTGTGCCGCTGGGGGCTCCTCCTCGCCCTCTTGCCCCCCGG
AGCCGCGAGCACCCAAGTGTGCACCGGCACAGACATGAAGCTGCGGCTCCCTGCCA
GTCCCGAGACCCACCTGGACATGCTCCGCCACCTCTACCAGGGCTGCCAGGTGGTG
CAGGGAAACCTGGAACTCACCTACCTGCCCACCAATGCCAGCCTGTCCTTCCTGCAG
GATATCCAGGAGGTGCAGGGCTACGTGCTCATCGCTCACAACCAAGTGAGGCAGGT
CCCACTGCAGAGGCTGCGGATTGTGCGAGGCACCCAGCTCTTTGAGGACAACTATGC
CCTGGCCGTGCTAGACAATGGAGACCCGCTGAACAATACCACCCCTGTCACAGGGGC

CTCCCCAGGAGGCCTGCGGGAGCTGCAGCTTCGAAGCCTCACAGAGATCTTGAAAG
GAGGGGTCTTGATCCAGCGGAACCCCCAGCTCTGCTACCAGGACACGATTTTGTGGA
AGGACATCTTCCACAAGAACAACCAGCTGGCTCTCACACTGATAGACACCAACCGCT
CTCGGGCCTGCCACCCCTGTTCTCCGATGTGTAAGGGCTCCCGCTGCTGGGGAGAG
AGTTCTGAGGATTGTCAGAGCCTGACGCGCACTGTCTGTGCCGGTGGCTGTGCCCG
CTGCAAGGGGCCACTGCCCACTGACTGCTGCCATGAGCAGTGTGCTGCCGGCTGCA
CGGGCCCCAAGCACTCTGACTGCCTGGCCTGCCTCCACTTCAACCACAGTGGCATCT
GTGAGCTGCACTGCCCAGCCCTGGTCACCTACAACACAGACACGTTTGAGTCCATGC
CCAATCCCGAGGGCCGGTATACATTCGGCGCCAGCTGTGTGACTGCCTGTCCCTACA
ACTACCTTTCTACGGACGTGGGATCCTGCACCCTCGTCTGCCCCCTGCACAACCAAG
AGGTGACAGCAGAGGATGGAACACAGCGGTGTGAGAAGTGCAGCAAGCCCTGTGCC
CGAGTGTGCTATGGTCTGGGCATGGAGCACTTGCGAGAGGTGAGGGCAGTTACCAG
TGCCAATATCCAGGAGTTTGCTGGCTGCAAGAAGATCTTTGGGAGCCTGGCATTTCT
GCCGGAGAGCTTTGATGGGGACCCAGCCTCCAACACTGCCCCGCTCCAGCCAGAGC
AGCTCCAAGTGTTTGAGACTCTGGAAGAGATCACAGGTTACCTATACATCTCAGCAT
GGCCGGACAGCCTGCCTGACCTCAGCGTCTTCCAGAACCTGCAAGTAATCCGGGGA
CGAATTCTGCACAATGGCGCCTACTCGCTGACCCTGCAAGGGCTGGGCATCAGCTG
GCTGGGGCTGCGCTCACTGAGGGAACTGGGCAGTGGACTGGCCCTCATCCACCATA
ACACCCACCTCTGCTTCGTGCACACGGTGCCCTGGGACCAGCTCTTTCGGAACCCGC
ACCAAGCTCTGCTCCACACTGCCAACCGGCCAGAGGACGAGTGTGTGGGCGAGGGC
CTGGCCTGCCACCAGCTGTGCGCCCGAGGGCACTGCTGGGGTCCAGGGCCCACCCA
GTGTGTCAACTGCAGCCAGTTCCTTCGGGGCCAGGAGTGCGTGGAGGAATGCCGAG
TACTGCAGGGGCTCCCCAGGGAGTATGTGAATGCCAGGCACTGTTTGCCGTGCCAC
CCTGAGTGTCAGCCCCAGAATGGCTCAGTGACCTGTTTTGGACCGGAGGCTGACCA
GTGTGTGGCCTGTGCCCACTATAAGGACCCTCCCTTCTGCGTGGCCCGCTGCCCCA
GCGGTGTGAAACCTGACCTCTCCTACATGCCCATCTGGAAGTTTCCAGATGAGGAGG
GCGCATGCCAGCCTTGCCCCATCAACTGCACCCACTCCTGTGTGGACCTGGATGACA
AGGGCTGCCCCGCCGAGCAGAGAGCCAGCCCTggcggccgctcgagcGGTGGTGGAGGG
TCAGGAGGTGGAGGGtcgagcGGTGGTGGAGGGTCAGGAGGTGGAGGGtcgagcGGTG
GTGGAGGGTCAGGAGGTGGAGGGtcgagcgccATGGCCAGATCTGGGtcgagcggcGTCT
TCACACTCGAAGATTTCGTTGGGGACTGGGAACAGACAGCCGCCTACAACCTGGACC
AAGTCCTTGAACAGGGAGGTGTGTCCAGTTTGCTGCAGAATCTCGCCGTGTCCGTAA
CTCCGATCCAAAGGATTGTCCGTAGCGGTGAAAATGCCCTGAAGATCGACATCCATG
TCATCATCCCGTATGAAGGTCTGAGCGCCGACCAAATGGCCCAGATCGAAGAGGTGT
TTAAGGTGGTGTACCCTGTGGATGATCATCACTTTAAGGTGATCCTGCCCTATGGCA
CACTGGTAATCGACGGGGTTACGCCGAACATGCTGAACTATTTCGGACGGCCGTATG
AAGGCATCGCCGTGTTCGACGGCAAAAAGATCACTGTAACAGGGACCCTGTGGAACG
GCAACAAAATTATCGACGAGCGCCTGATCACCCCCGACGGCTCCATGCTGTTCCGAG
TAACCATCAACAGTTAA

⑤　anti-HER2-SmBiT

ATGAACTCCTTCTCCACAAGCGCCTTCGGTCCAGTTGCCTTCTCCCTGGGCCTGCTC
CTGGTGTTGCCTGCTGCCTTCCCTGCCCCAgtcccctccggaggtggagggtcgtcaggtGTGG
ACAATAAATTCAACAAAGAAATGAGAAACGCCTACTGGGAGATCGCCCTGCTACCCA
ACCTGAACAACCAACAGAAGAGAGCGTTTATCAGAAGCCTGTACGACGACCCTAGCC
AATCTGCTAACCTGCTGGCAGAGGCTAAGAAACTGAATGACGCCCAAGCCCCTAAGg
gcggtggaggctcgagcggtGTGACCGGCTACCGGCTGTTCGAGGAGATTCTGtaa

⑥　anti-HER2-Linker-SmBiT

ATGAACTCCTTCTCCACAAGCGCCTTCGGTCCAGTTGCCTTCTCCCTGGGCCTGCTC
CTGGTGTTGCCTGCTGCCTTCCCTGCCCCAgtcccctccggaggtggagggtcgtcaggtGTGG
ACAATAAATTCAACAAAGAAATGAGAAACGCCTACTGGGAGATCGCCCTGCTACCCA

ACCTGAACAACCAACAGAAGAGAGCGTTTATCAGAAGCCTGTACGACGACCCTAGCC
AATCTGCTAACCTGCTGGCAGAGGCTAAGAAACTGAATGACGCCCAAGCCCCTAAGg
gcggtggaggctcgagcGGTGGTGGAGGGTCAGGAGGTGGAGGGtcgagcGGTGGTGGAGG
GTCAGGAGGTGGAGGGtcgagcGGTGGTGGAGGGTCAGGAGGTGGAGGGtcgagcgccA
TGGGCTCCGGAGGCGGATCCGGTGGGtcgagcggtGTGACCGGCTACCGGCTGTTCGA
GGAGATTCTGTAA

⑦ anti-HER2-LgBiT

ATGAACTCCTTCTCCACAAGCGCCTTCGGTCCAGTTGCCTTCTCCCTGGGCCTGCTC
CTGGTGTTGCCTGCTGCCTTCCCTGCCCCAgtcccctccggaggtggagggtcgtcaggtGTGG
ACAATAAATTCAACAAAGAAATGAGAAACGCCTACTGGGAGATCGCCCTGCTACCCA
ACCTGAACAACCAACAGAAGAGAGCGTTTATCAGAAGCCTGTACGACGACCCTAGCC
AATCTGCTAACCTGCTGGCAGAGGCTAAGAAACTGAATGACGCCCAAGCCCCTAAGg
gcggtggaggctcgagcggcGTCTTCACACTCGAAGATTTCGTTGGGGACTGGGAACAGAC
AGCCGCCTACAACCTGGACCAAGTCCTTGAACAGGGAGGTGTGTCCAGTTTGCTGCA
GAATCTCGCCGTGTCCGTAACTCCGATCCAAAGGATTGTCCGTAGCGGTGAAAATGC
CCTGAAGATCGACATCCATGTCATCATCCCGTATGAAGGTCTGAGCGCCGACCAAAT
GGCCCAGATCGAAGAGGTGTTTAAGGTGGTGTACCCTGTGGATGATCATCACTTTAA
GGTGATCCTGCCCTATGGCACACTGGTAATCGACGGGGTTACGCCGAACATGCTGAA
CTATTTCGGACGGCCGTATGAAGGCATCGCCGTGTTCGACGGCAAAAAGATCACTGT
AACAGGGACCCTGTGGAACGGCAACAAAATTATCGACGAGCGCCTGATCACCCCCGA
CGGCTCCATGCTGTTCCGAGTAACCATCAACAGTTAA

⑧ anti-HER2-Linker-LgBiT

ATGAACTCCTTCTCCACAAGCGCCTTCGGTCCAGTTGCCTTCTCCCTGGGCCTGCTC
CTGGTGTTGCCTGCTGCCTTCCCTGCCCCAgtcccctccggaggtggagggtcgtcaggtGTGG
ACAATAAATTCAACAAAGAAATGAGAAACGCCTACTGGGAGATCGCCCTGCTACCCA
ACCTGAACAACCAACAGAAGAGAGCGTTTATCAGAAGCCTGTACGACGACCCTAGCC
AATCTGCTAACCTGCTGGCAGAGGCTAAGAAACTGAATGACGCCCAAGCCCCTAAGg
gcggtggaggctcgagcGGTGGTGGAGGGTCAGGAGGTGGAGGGtcgagcGGTGGTGGAGG
GTCAGGAGGTGGAGGGtcgagcGGTGGTGGAGGGTCAGGAGGTGGAGGGtcgagcgccA
TGGCCAGATCTGGGtcgagcggcGTCTTCACACTCGAAGATTTCGTTGGGGACTGGGAA
CAGACAGCCGCCTACAACCTGGACCAAGTCCTTGAACAGGGAGGTGTGTCCAGTTTG
CTGCAGAATCTCGCCGTGTCCGTAACTCCGATCCAAAGGATTGTCCGTAGCGGTGAA
AATGCCCTGAAGATCGACATCCATGTCATCATCCCGTATGAAGGTCTGAGCGCCGAC
CAAATGGCCCAGATCGAAGAGGTGTTTAAGGTGGTGTACCCTGTGGATGATCATCAC
TTTAAGGTGATCCTGCCCTATGGCACACTGGTAATCGACGGGGTTACGCCGAACATG
CTGAACTATTTCGGACGGCCGTATGAAGGCATCGCCGTGTTCGACGGCAAAAAGATC
ACTGTAACAGGGACCCTGTGGAACGGCAACAAAATTATCGACGAGCGCCTGATCACC
CCCGACGGCTCCATGCTGTTCCGAGTAACCATCAACAGTTAA

Supplementary Table 4

DNA sequences in the plasmid constructs of Supplementary Fig 3.

ZZ-Af-IFNAR1-TM-IN-394

ATGAACTCCTTCTCCACAAGCGCCTTCGGTCCAGTTGCCTTCTCCCTGGGCCTGCTC
CTGGTGTTGCCTGCTGCCTTCCCTGCCCCAgtcccctccggaggtggagggtcgtcaggtGTAG

ACAACAAGTTCAACAAGGAACAACAAAACGCGTTCTATGAAATCTTACATTTACCTAA
CTTAAACGAAGAACAACGAAACGCCTTCATCCAAAGTTTAAAAGATGACCCAAGCCAA
AGCGCTAACCTTTTAGCAGAAGCTAAAAAGCTAAATGATGCTCAGGCGCCGAAAGTT
GATAATAAGTTTAATAAGGAGCAGCAGAATGCATTTTACGAAATTCTTCACCTTCCAA
ATCTTAATGAGGAACAGAGAAATGCATTTATTCAGTCACTTAAGGACGATCCTTCTCA
GTCTGCAAATTTACTTGCTGAAGCAAAGAAGTTAAACGATGCACAAGCTCCTAAGggct
cgagtCCTAATTTGAAACCACTGACTGTATATTGTGTGAAAGCCAGAGCACACACCATG
GATGAAAAGCTGAATAAAAGCAGTGTTTTTAGTGACGCTGTATGTGAGAAAACAAAAC
CAGGAAATACCTCTAAAATTTGGCTTATAGTTGGAATTTGTATTGCATTATTTGCTCT
CCCGTTTGTCATTTATGCTGCGAAAGTCTTCTTGAGATGCATCAATTATGTCTTCTTT
CCATCACTTAAACCTTCTTCCAGTATAGATGAGTATTTCTCTGAACAGCCATTGAAGA
ATCTTCTGCTTTCAACTTCTGAGGAACAAATCGAAAAATGTTTCATAATTGAAAATAT
AAGCACAATTGCTACAGTAGAAGAAACTAATCAAACTGATGAAGATCATAAAAAATAC
AGTTCCCAAACTAGCCAAGATTCAGGAAATTATTCTAATGAAGATGAAAGCGAAAGTA
AAACAAGTGAAGAACTACAGCAGGACTTTGTATGA

PD-L1-EX-IFNAR2-TM-IN-231

ATGAGGATATTTGCTGTCTTTATATTCATGACCTACTGGCATTTGCTGAACGCATTTA
CTGTCACGGTTCCCAAGGACCTATATGTGGTAGAGTATGGTAGCAATATGACAATTG
AATGCAAATTCCCAGTAGAAAAACAATTAGACCTGGCTGCACTAATTGTCTATTGGGA
AATGGAGGATAAGAACATTATTCAATTTGTGCATGGAGAGGAAGACCTGAAGGTTCA
GCATAGTAGCTACAGACAGAGGGCCCGGCTGTTGAAGGACCAGCTCTCCCTGGGAA
ATGCTGCACTTCAGATCACAGATGTGAAATTGCAGGATGCAGGGGTGTACCGCTGCA
TGATCAGCTATGGTGGTGCCGACTACAAGCGAATTACTGTGAAAGTCAATGCCCCAT
ACAACAAAATCAACCAAAGAATTTTGGTTGTGGATCCAGTCACCTCTGAACATGAACT
GACATGTCAGGCTGAGGGCTACCCCAAGGCCGAAGTCATCTGGACAAGCAGTGACC
ATCAAGTCCTGAGTGGTAAGACCACCACCACCAATTCCAAGAGAGAGGAGAAGCTTT
TCAATGTGACCAGCACACTGAGAATCAACACAACAACTAATGAGATTTTCTACTGCAC
TTTTAGGAGATTAGATCCTGAGGAAAACCATACAGCTGAATTGGTCATCCCAGAACTA
CCTCTGGCACATCCTCCAAActcgagtCCACCTGGCCAGGAATCAGAATCAGCAGAATC
TGCCAAAATAGGAGGAATAATTACTGTGTTTTTGATAGCATTGGTCTTGACAAGCACC
ATAGTGACACTGAAATGGATTGGTTATATATGCTTAAGAAATAGCCTCCCCAAAGTCT
TGAATTTTCATAACTTTTTAGCCTGGCCATTTCCTAACCTGCCACCGTTGGAAGCCAT
GGATATGGTGGAGGTCATTTACATCAACAGAAAGAAGAAAGTGTGGGATTATAATTA
TGATGATGAAAGTGATAGCGATACTGAGGCAGCGCCCAGGACAAGTGGCGGTGGCT
ATACCATGCATGGACTGACTGTCAGGCCTCTGGGTCAGGCCTCTGCCACCTCTACAG
AATCCCAGTTGATAGACCCGGAGTCCGAGGAGGAGCCTGACCTGCCTGAGGTTGAT
GTGGAGCTCCCCACGATGCCAAAGGACAGCCCTCAGCAGTTGGAACTCTTGAGTGG
GCCCTGTGAGAGGAGAAAGAGTCCACTCCAGGACCCTTTTCCCGAAGAGGACTACAG
CTCCACGGAGGGGTCTGGGGGCAGAATTACCTTCAATGTGGACTTAAACTCTGTGTT
TTTGAGAGTTCTTGATGACGAGGACAGTGACGACTTAGAAGCCCCTCTGATGCTATC
GTCTCATCTGGAAGAGATGGTTGACCCAGAGGATCCTGATAATGTGCAATCAAACCA
TTTGCTGGCCAGCGGGGAAGGGACACAGCCAACCTTTCCCAGCCCCTCTTCAGAGG
GCCTGTGGTCCGAAGATGCTCCATCTGATCAAAGTGACACTTCTGAGTCAGATGTTG
ACCTTGGGGATGGTTATATAATGAGATGA

DNA sequences in the plasmid constructs of Supplementary Fig 4.

HER2-EX-IFNAR1-TM-IN-394

ATGGAGCTGGCGGCCTTGTGCCGCTGGGGGCTCCTCCTCGCCCTCTTGCCCCCCGG
AGCCGCGAGCACCCAAGTGTGCACCGGCACAGACATGAAGCTGCGGCTCCCTGCCA
GTCCCGAGACCCACCTGGACATGCTCCGCCACCTCTACCAGGGCTGCCAGGTGGTG

CAGGGAAACCTGGAACTCACCTACCTGCCCACCAATGCCAGCCTGTCCTTCCTGCAG
GATATCCAGGAGGTGCAGGGCTACGTGCTCATCGCTCACAACCAAGTGAGGCAGGT
CCCACTGCAGAGGCTGCGGATTGTGCGAGGCACCCAGCTCTTTGAGGACAACTATGC
CCTGGCCGTGCTAGACAATGGAGACCCGCTGAACAATACCACCCCTGTCACAGGGGC
CTCCCCAGGAGGCCTGCGGGAGCTGCAGCTTCGAAGCCTCACAGAGATCTTGAAAG
GAGGGGTCTTGATCCAGCGGAACCCCCAGCTCTGCTACCAGGACACGATTTTGTGGA
AGGACATCTTCCACAAGAACAACCAGCTGGCTCTCACACTGATAGACACCAACCGCT
CTCGGGCCTGCCACCCCTGTTCTCCGATGTGTAAGGGCTCCCGCTGCTGGGGAGAG
AGTTCTGAGGATTGTCAGAGCCTGACGCGCACTGTCTGTGCCGGTGGCTGTGCCCG
CTGCAAGGGGCCACTGCCCACTGACTGCTGCCATGAGCAGTGTGCTGCCGGCTGCA
CGGGCCCCAAGCACTCTGACTGCCTGGCCTGCCTCCACTTCAACCACAGTGGCATCT
GTGAGCTGCACTGCCCAGCCCTGGTCACCTACAACACAGACACGTTTGAGTCCATGC
CCAATCCCGAGGGCCGGTATACATTCGGCGCCAGCTGTGTGACTGCCTGTCCCTACA
ACTACCTTTCTACGGACGTGGGATCCTGCACCCTCGTCTGCCCCCTGCACAACCAAG
AGGTGACAGCAGAGGATGGAACACAGCGGTGTGAGAAGTGCAGCAAGCCCTGTGCC
CGAGTGTGCTATGGTCTGGGCATGGAGCACTTGCGAGAGGTGAGGGCAGTTACCAG
TGCCAATATCCAGGAGTTTGCTGGCTGCAAGAAGATCTTTGGGAGCCTGGCATTTCT
GCCGGAGAGCTTTGATGGGGACCCAGCCTCCAACACTGCCCCGCTCCAGCCAGAGC
AGCTCCAAGTGTTTGAGACTCTGGAAGAGATCACAGGTTACCTATACATCTCAGCAT
GGCCGGACAGCCTGCCTGACCTCAGCGTCTTCCAGAACCTGCAAGTAATCCGGGGA
CGAATTCTGCACAATGGCGCCTACTCGCTGACCCTGCAAGGGCTGGGCATCAGCTG
GCTGGGGCTGCGCTCACTGAGGGAACTGGGCAGTGGACTGGCCCTCATCCACCATA
ACACCCACCTCTGCTTCGTGCACACGGTGCCCTGGGACCAGCTCTTTCGGAACCCGC
ACCAAGCTCTGCTCCACACTGCCAACCGGCCAGAGGACGAGTGTGTGGGCGAGGGC
CTGGCCTGCCACCAGCTGTGCGCCCGAGGGCACTGCTGGGGTCCAGGGCCCACCCA
GTGTGTCAACTGCAGCCAGTTCCTTCGGGGCCAGGAGTGCGTGGAGGAATGCCGAG
TACTGCAGGGGCTCCCCAGGGAGTATGTGAATGCCAGGCACTGTTTGCCGTGCCAC
CCTGAGTGTCAGCCCCAGAATGGCTCAGTGACCTGTTTTGGACCGGAGGCTGACCA
GTGTGTGGCCTGTGCCCACTATAAGGACCCTCCCTTCTGCGTGGCCCGCTGCCCCA
GCGGTGTGAAACCTGACCTCTCCTACATGCCCATCTGGAAGTTTCCAGATGAGGAG
GCGCATGCCAGCCTTGCCCCATCAACTGCACCCACTCCTGTGTGGACCTGGATGACA
AGGGCTGCCCCGCCGAGCAGAGAGCCAGCCCTggcggccgctcgagtCCTAATTTGAAAC
CACTGACTGTATATTGTGTGAAAGCCAGAGCACACACCATGGATGAAAAGCTGAATA
AAAGCAGTGTTTTTAGTGACGCTGTATGTGAGAAAACAAAACCAGGAAATACCTCTAA
AATTTGGCTTATAGTTGGAATTTGTATTGCATTATTTGCTCTCCCGTTTGTCATTTAT
GCTGCGAAAGTCTTCTTGAGATGCATCAATTATGTCTTCTTTCCATCACTTAAACCTT
CTTCCAGTATAGATGAGTATTTCTCTGAACAGCCATTGAAGAATCTTCTGCTTTCAAC
TTCTGAGGAACAAATCGAAAAATGTTTCATAATTGAAAATATAAGCACAATTGCTACA
GTAGAAGAAACTAATCAAACTGATGAAGATCATAAAAAATACAGTTCCCAAACTAGCC
AAGATTCAGGAAATTATTCTAATGAAGATGAAAGCGAAAGTAAAACAAGTGAAGAACT
ACAGCAGGACTTTGTATGA

PD1-EX-Linker-anti-HER2-Af

ATGCAGATCCCACAGGCGCCCTGGCCAGTCGTCTGGGCGGTGCTACAACTGGGCTG
GCGGCCAGGATGGTTCTTAGACTCCCCAGACAGGCCCTGGAACCCCCCCACCTTCTC
CCCAGCCCTGCTCGTGGTGACCGAAGGGGACAACGCCACCTTCACCTGCAGCTTCTC
CAACACATCGGAGAGCTTCGTGCTAAACTGGTACCGCATGAGCCCCAGCAACCAGAC
GGACAAGCTGGCCGCCTTCCCCGAGGACCGCAGCCAGCCCGGCCAGGACTGCCGCT
TCCGTGTCACACAACTGCCCAACGGGCGTGACTTCCACATGAGCGTGGTCAGGGCC
CGGCGCAATGACAGCGGCACCTACCTCTGTGGGGCCATCTCCCTGGCCCCCAAGGC
GCAGATCAAAGAGAGCCTGCGGGCAGAGCTCAGGGTGACAGAGAGAAGGGCAGAAG
TGCCCACAGCCCACCCCAGCCCCTCACCCAGGCCAGCCGGCCAGTTCCAAACctcgag
cGGTGGTGGAGGGTCAGGAGGTGGAGGGtcgagcGGTGGTGGAGGGTCAGGAGGTGG
AGGGtcgagcGGTGGTGGAGGGTCAGGAGGTGGAGGGtcgagcgccATGGCCGGAACTA
CCGTGGACAATAAATTCAACAAAGAAATGAGAAACGCCTACTGGGAGATCGCCCTGC
TACCCAACCTGAACAACCAACAGAAGAGAGCGTTTATCAGAAGCCTGTACGACGACC

CTAGCCAATCTGCTAACCTGCTGGCAGAGGCTAAGAAACTGAATGACGCCCAAGCCC
CTAAGGGCGGCCGCTAA

Supplementary Table 5

DNA sequences in the plasmid constructs of Supplementary Fig 5.

① HER2-EX-TM-IFNAR2-IN-265

ATGGAGCTGGCGGCCTTGTGCCGCTGGGGGCTCCTCCTCGCCCTCTTGCCCCCCGG
AGCCGCGAGCACCCAAGTGTGCACCGGCACAGACATGAAGCTGCGGCTCCCTGCCA
GTCCCGAGACCCACCTGGACATGCTCCGCCACCTCTACCAGGGCTGCCAGGTGGTG
CAGGGAAACCTGGAACTCACCTACCTGCCCACCAATGCCAGCCTGTCCTTCCTGCAG
GATATCCAGGAGGTGCAGGGCTACGTGCTCATCGCTCACAACCAAGTGAGGCAGGT
CCCACTGCAGAGGCTGCGGATTGTGCGAGGCACCCAGCTCTTTGAGGACAACTATGC
CCTGGCCGTGCTAGACAATGGAGACCCGCTGAACAATACCACCCCTGTCACAGGGGC
CTCCCCAGGAGGCCTGCGGGAGCTGCAGCTTCGAAGCCTCACAGAGATCTTGAAAG
GAGGGGTCTTGATCCAGCGGAACCCCCAGCTCTGCTACCAGGACACGATTTTGTGGA
AGGACATCTTCCACAAGAACAACCAGCTGGCTCTCACACTGATAGACACCAACCGCT
CTCGGGCCTGCCACCCCTGTTCTCCGATGTGTAAGGGCTCCCGCTGCTGGGGAGAG
AGTTCTGAGGATTGTCAGAGCCTGACGCGCACTGTCTGTGCCGGTGGCTGTGCCCG
CTGCAAGGGGCCACTGCCCACTGACTGCTGCCATGAGCAGTGTGCTGCCGGCTGCA
CGGGCCCCAAGCACTCTGACTGCCTGGCCTGCCTCCACTTCAACCACAGTGGCATCT
GTGAGCTGCACTGCCCAGCCCTGGTCACCTACAACACAGACACGTTTGAGTCCATGC
CCAATCCCGAGGGCCGGTATACATTCGGCGCCAGCTGTGTGACTGCCTGTCCCTACA
ACTACCTTTCTACGGACGTGGGATCCTGCACCCTCGTCTGCCCCCTGCACAACCAAG
AGGTGACAGCAGAGGATGGAACACAGCGGTGTGAGAAGTGCAGCAAGCCCTGTGCC
CGAGTGTGCTATGGTCTGGGCATGGAGCACTTGCGAGAGGTGAGGGCAGTTACCAG
TGCCAATATCCAGGAGTTTGCTGGCTGCAAGAAGATCTTTGGGAGCCTGGCATTTCT
GCCGGAGAGCTTTGATGGGGACCCAGCCTCCAACACTGCCCCGCTCCAGCCAGAGC
AGCTCCAAGTGTTTGAGACTCTGGAAGAGATCACAGGTTACCTATACATCTCAGCAT
GGCCGGACAGCCTGCCTGACCTCAGCGTCTTCCAGAACCTGCAAGTAATCCGGGGA
CGAATTCTGCACAATGGCGCCTACTCGCTGACCCTGCAAGGGCTGGGCATCAGCTG
GCTGGGGCTGCGCTCACTGAGGGAACTGGGCAGTGGACTGGCCCTCATCCACCATA
ACACCCACCTCTGCTTCGTGCACACGGTGCCCTGGGACCAGCTCTTTCGGAACCCGC
ACCAAGCTCTGCTCCACACTGCCAACCGGCCAGAGGACGAGTGTGTGGGCGAGGGC
CTGGCCTGCCACCAGCTGTGCGCCCGAGGGCACTGCTGGGGTCCAGGGCCCACCCA
GTGTGTCAACTGCAGCCAGTTCCTTCGGGGCCAGGAGTGCGTGGAGGAATGCCGAG
TACTGCAGGGGCTCCCCAGGGAGTATGTGAATGCCAGGCACTGTTTGCCGTGCCAC
CCTGAGTGTCAGCCCCAGAATGGCTCAGTGACCTGTTTTGGACCGGAGGCTGACCA
GTGTGTGGCCTGTGCCCACTATAAGGACCCTCCCTTCTGCGTGGCCCGCTGCCCCA
GCGGTGTGAAACCTGACCTCTCCTACATGCCCATCTGGAAGTTTCCAGATGAGGAG
GCGCATGCCAGCCTTGCCCCATCAACTGCACCCACTCCTGTGTGGACCTGGATGACA
AGGGCTGCCCCGCCGAGCAGAGAGCCAGCCCTCTGACGTCCATCATCTCTGCGGTG
GTTGGCATTCTGCTGGTCGTGGTCTTGGGGGTGGTCTTTGGGATCCTCATCAAGCGA
CGGCAGCAGAAGATCCGGAAGTActcgagcAAATGGATTGGTTATATATGCTTAAGAAA
TAGCCTCCCCAAAGTCTTGAATTTTCATAACTTTTTAGCCTGGCCATTTCCTAACCTG
CCACCGTTGGAAGCCATGGATATGGTGGAGGTCATTTACATCAACAGAAAGAAGAAA
GTGTGGGATTATAATTATGATGATGAAAGTGATAGCGATACTGAGGCAGCGCCCAGG
ACAAGTGGCGGTGGCTATACCATGCATGGACTGACTGTCAGGCCTCTGGGTCAGGC
CTCTGCCACCTCTACAGAATCCCAGTTGATAGACCCGGAGTCCGAGGAGGAGCCTGA
CCTGCCTGAGGTTGATGTGGAGCTCCCCACGATGCCAAAGGACAGCCCTCAGCAGTT
GGAACTCTTGAGTGGGCCCTGTGAGAGGAGAAAGAGTCCACTCCAGGACCCTTTTCC
CGAAGAGGACTACAGCTCCACGGAGGGGTCTGGGGGCAGAATTACCTTCAATGTGG
ACTTAAACTCTGTGTTTTTGAGAGTTCTTGATGACGAGGACAGTGACGACTTAGAAG
CCCCTCTGATGCTATCGTCTCATCTGGAAGAGATGGTTGACCCAGAGGATCCTGATA
ATGTGCAATCAAACCATTTGCTGGCCAGCGGGGAAGGGACACAGCCAACCTTTCCCA

GCCCCTCTTCAGAGGGCCTGTGGTCCGAAGATGCTCCATCTGATCAAAGTGACACTT
CTGAGTCAGATGTTGACCTTGGGGATGGTTATATAATGAGATGA

② HER2-EX-TM-IFNAR1-IN-458

ATGGAGCTGGCGGCCTTGTGCCGCTGGGGGCTCCTCCTCGCCCTCTTGCCCCCCGG
AGCCGCGAGCACCCAAGTGTGCACCGGCACAGACATGAAGCTGCGGCTCCCTGCCA
GTCCCGAGACCCACCTGGACATGCTCCGCCACCTCTACCAGGGCTGCCAGGTGGTG
CAGGGAAACCTGGAACTCACCTACCTGCCCACCAATGCCAGCCTGTCCTTCCTGCAG
GATATCCAGGAGGTGCAGGGCTACGTGCTCATCGCTCACAACCAAGTGAGGCAGGT
CCCACTGCAGAGGCTGCGGATTGTGCGAGGCACCCAGCTCTTTGAGGACAACTATGC
CCTGGCCGTGCTAGACAATGGAGACCCGCTGAACAATACCACCCCTGTCACAGGGGC
CTCCCCAGGAGGCCTGCGGGAGCTGCAGCTTCGAAGCCTCACAGAGATCTTGAAAG
GAGGGGTCTTGATCCAGCGGAACCCCCAGCTCTGCTACCAGGACACGATTTTGTGGA
AGGACATCTTCCACAAGAACAACCAGCTGGCTCTCACACTGATAGACACCAACCGCT
CTCGGGCCTGCCACCCCTGTTCTCCGATGTGTAAGGGCTCCCGCTGCTGGGGAGAG
AGTTCTGAGGATTGTCAGAGCCTGACGCGCACTGTCTGTGCCGGTGGCTGTGCCCG
CTGCAAGGGGCCACTGCCCACTGACTGCTGCCATGAGCAGTGTGCTGCCGGCTGCA
CGGGCCCCAAGCACTCTGACTGCCTGGCCTGCCTCCACTTCAACCACAGTGGCATCT
GTGAGCTGCACTGCCCAGCCCTGGTCACCTACAACACAGACACGTTTGAGTCCATGC
CCAATCCCGAGGGCCGGTATACATTCGGCGCCAGCTGTGTGACTGCCTGTCCCTACA
ACTACCTTTCTACGGACGTGGGATCCTGCACCCTCGTCTGCCCCCTGCACAACCAAG
AGGTGACAGCAGAGGATGGAACACAGCGGTGTGAGAAGTGCAGCAAGCCCTGTGCC
CGAGTGTGCTATGGTCTGGGCATGGAGCACTTGCGAGAGGTGAGGGCAGTTACCAG
TGCCAATATCCAGGAGTTTGCTGGCTGCAAGAAGATCTTTGGGAGCCTGGCATTTCT
GCCGGAGAGCTTTGATGGGGACCCAGCCTCCAACACTGCCCCGCTCCAGCCAGAGC
AGCTCCAAGTGTTTGAGACTCTGGAAGAGATCACAGGTTACCTATACATCTCAGCAT
GGCCGGACAGCCTGCCTGACCTCAGCGTCTTCCAGAACCTGCAAGTAATCCGGGGA
CGAATTCTGCACAATGGCGCCTACTCGCTGACCCTGCAAGGGCTGGGCATCAGCTG
GCTGGGGCTGCGCTCACTGAGGGAACTGGGCAGTGGACTGGCCCTCATCCACCATA
ACACCCACCTCTGCTTCGTGCACACGGTGCCCTGGGACCAGCTCTTTCGGAACCCGC
ACCAAGCTCTGCTCCACACTGCCAACCGGCCAGAGGACGAGTGTGTGGGCGAGGGC
CTGGCCTGCCACCAGCTGTGCGCCCGAGGGCACTGCTGGGGTCCAGGGCCCACCCA
GTGTGTCAACTGCAGCCAGTTCCTTCGGGGCCAGGAGTGCGTGGAGGAATGCCGAG
TACTGCAGGGGCTCCCCAGGGAGTATGTGAATGCCAGGCACTGTTTGCCGTGCCAC
CCTGAGTGTCAGCCCCAGAATGGCTCAGTGACCTGTTTTGGACCGGAGGCTGACCA
GTGTGTGGCCTGTGCCCACTATAAGGACCCTCCCTTCTGCGTGGCCCGCTGCCCCA
GCGGTGTGAAACCTGACCTCTCCTACATGCCCATCTGGAAGTTTCCAGATGAGGAG
GCGCATGCCAGCCTTGCCCCATCAACTGCACCCACTCCTGTGTGGACCTGGATGACA
AGGGCTGCCCCGCCGAGCAGAGAGCCAGCCCTCTGACGTCCATCATCTCTGCGGTG
GTTGGCATTCTGCTGGTCGTGGTCTTGGGGGTGGTCTTTGGGATCCTCATCAAGCGA
CGGCAGCAGAAGATCCGGAAGTActcgagcAAAGTCTTCTTGAGATGCATCAATTATGT
CTTCTTTCCATCACTTAAACCTTCTTCCAGTATAGATGAGTATTTCTCTGAACAGCCA
TTGAAGAATCTTCTGCTTTCAACTTCTGAGGAACAAATCGAAAAATGTTTCATAATTG
AAAATATAAGCACAATTGCTACAGTAGAAGAAACTAATCAAACTGATGAAGATCATAA
AAAATACAGTTCCCAAACTAGCCAAGATTCAGGAAATTATTCTAATGAAGATGAAAGC
GAAAGTAAAACAAGTGAAGAACTACAGCAGGACTTTGTATGA

③ HER2-EX-TM-IFNAR1-IN-D51

ATGGAGCTGGCGGCCTTGTGCCGCTGGGGGCTCCTCCTCGCCCTCTTGCCCCCCGG
AGCCGCGAGCACCCAAGTGTGCACCGGCACAGACATGAAGCTGCGGCTCCCTGCCA
GTCCCGAGACCCACCTGGACATGCTCCGCCACCTCTACCAGGGCTGCCAGGTGGTG
CAGGGAAACCTGGAACTCACCTACCTGCCCACCAATGCCAGCCTGTCCTTCCTGCAG

GATATCCAGGAGGTGCAGGGCTACGTGCTCATCGCTCACAACCAAGTGAGGCAGGT
CCCACTGCAGAGGCTGCGGATTGTGCGAGGCACCCAGCTCTTTGAGGACAACTATGC
CCTGGCCGTGCTAGACAATGGAGACCCGCTGAACAATACCACCCCTGTCACAGGGGC
CTCCCCAGGAGGCCTGCGGGAGCTGCAGCTTCGAAGCCTCACAGAGATCTTGAAAG
GAGGGGTCTTGATCCAGCGGAACCCCCAGCTCTGCTACCAGGACACGATTTTGTGGA
AGGACATCTTCCACAAGAACAACCAGCTGGCTCTCACACTGATAGACACCAACCGCT
CTCGGGCCTGCCACCCCTGTTCTCCGATGTGTAAGGGCTCCCGCTGCTGGGGAGAG
AGTTCTGAGGATTGTCAGAGCCTGACGCGCACTGTCTGTGCCGGTGGCTGTGCCCG
CTGCAAGGGGCCACTGCCCACTGACTGCTGCCATGAGCAGTGTGCTGCCGGCTGCA
CGGGCCCCAAGCACTCTGACTGCCTGGCCTGCCTCCACTTCAACCACAGTGGCATCT
GTGAGCTGCACTGCCCAGCCCTGGTCACCTACAACACAGACACGTTTGAGTCCATGC
CCAATCCCGAGGGCCGGTATACATTCGGCGCCAGCTGTGTGACTGCCTGTCCCTACA
ACTACCTTTCTACGGACGTGGGATCCTGCACCCTCGTCTGCCCCCTGCACAACCAAG
AGGTGACAGCAGAGGATGGAACACAGCGGTGTGAGAAGTGCAGCAAGCCCTGTGCC
CGAGTGTGCTATGGTCTGGGCATGGAGCACTTGCGAGAGGTGAGGGCAGTTACCAG
TGCCAATATCCAGGAGTTTGCTGGCTGCAAGAAGATCTTTGGGAGCCTGGCATTTCT
GCCGGAGAGCTTTGATGGGGACCCAGCCTCCAACACTGCCCCGCTCCAGCCAGAGC
AGCTCCAAGTGTTTGAGACTCTGGAAGAGATCACAGGTTACCTATACATCTCAGCAT
GGCCGGACAGCCTGCCTGACCTCAGCGTCTTCCAGAACCTGCAAGTAATCCGGGGA
CGAATTCTGCACAATGGCGCCTACTCGCTGACCCTGCAAGGGCTGGGCATCAGCTG
GCTGGGGCTGCGCTCACTGAGGGAACTGGGCAGTGGACTGGCCCTCATCCACCATA
ACACCCACCTCTGCTTCGTGCACACGGTGCCCTGGGACCAGCTCTTTCGGAACCCGC
ACCAAGCTCTGCTCCACACTGCCAACCGGCCAGAGGACGAGTGTGTGGGCGAGGGC
CTGGCCTGCCACCAGCTGTGCGCCCGAGGGCACTGCTGGGGTCCAGGGCCCACCCA
GTGTGTCAACTGCAGCCAGTTCCTTCGGGGCCAGGAGTGCGTGGAGGAATGCCGAG
TACTGCAGGGGCTCCCCAGGGAGTATGTGAATGCCAGGCACTGTTTGCCGTGCCAC
CCTGAGTGTCAGCCCCAGAATGGCTCAGTGACCTGTTTTGGACCGGAGGCTGACCA
GTGTGTGGCCTGTGCCCACTATAAGGACCCTCCCTTCTGCGTGGCCCGCTGCCCCA
GCGGTGTGAAACCTGACCTCTCCTACATGCCCATCTGGAAGTTTCCAGATGAGGAG
GCGCATGCCAGCCTTGCCCCATCAACTGCACCCACTCCTGTGTGGACCTGGATGACA
AGGGCTGCCCCGCCGAGCAGAGAGCCAGCCCTCTGACGTCCATCATCTCTGCGGTG
GTTGGCATTCTGCTGGTCGTGGTCTTGGGGGTGGTCTTTGGGATCCTCATCAAGCGA
CGGCAGCAGAAGATCCGGAAGTActcgagcAAAGTCTTCTTGAGATGCATCAATTATGT
CTTCTTTCCATCACTTAAACCTTCTTCCAGTATAGATGAGTATTTCTCTGAACAGCCA
TTGAAGAATCTTCTGCTTTCAACTTCTGAGGAACAAATCGAAAAATGTTTCATAATTG
AAAATATAAGCACAATTGCTACAGTAtaa

④　HER2-EX-TM-IFNAR1-IN-D43

ATGGAGCTGGCGGCCTTGTGCCGCTGGGGGCTCCTCCTCGCCCTCTTGCCCCCCGG
AGCCGCGAGCACCCAAGTGTGCACCGGCACAGACATGAAGCTGCGGCTCCCTGCCA
GTCCCGAGACCCACCTGGACATGCTCCGCCACCTCTACCAGGGCTGCCAGGTGGTG
CAGGGAAACCTGGAACTCACCTACCTGCCCACCAATGCCAGCCTGTCCTTCCTGCAG
GATATCCAGGAGGTGCAGGGCTACGTGCTCATCGCTCACAACCAAGTGAGGCAGGT
CCCACTGCAGAGGCTGCGGATTGTGCGAGGCACCCAGCTCTTTGAGGACAACTATGC
CCTGGCCGTGCTAGACAATGGAGACCCGCTGAACAATACCACCCCTGTCACAGGGGC
CTCCCCAGGAGGCCTGCGGGAGCTGCAGCTTCGAAGCCTCACAGAGATCTTGAAAG
GAGGGGTCTTGATCCAGCGGAACCCCCAGCTCTGCTACCAGGACACGATTTTGTGGA
AGGACATCTTCCACAAGAACAACCAGCTGGCTCTCACACTGATAGACACCAACCGCT
CTCGGGCCTGCCACCCCTGTTCTCCGATGTGTAAGGGCTCCCGCTGCTGGGGAGAG
AGTTCTGAGGATTGTCAGAGCCTGACGCGCACTGTCTGTGCCGGTGGCTGTGCCCG
CTGCAAGGGGCCACTGCCCACTGACTGCTGCCATGAGCAGTGTGCTGCCGGCTGCA
CGGGCCCCAAGCACTCTGACTGCCTGGCCTGCCTCCACTTCAACCACAGTGGCATCT
GTGAGCTGCACTGCCCAGCCCTGGTCACCTACAACACAGACACGTTTGAGTCCATGC
CCAATCCCGAGGGCCGGTATACATTCGGCGCCAGCTGTGTGACTGCCTGTCCCTACA
ACTACCTTTCTACGGACGTGGGATCCTGCACCCTCGTCTGCCCCCTGCACAACCAAG
AGGTGACAGCAGAGGATGGAACACAGCGGTGTGAGAAGTGCAGCAAGCCCTGTGCC

CGAGTGTGCTATGGTCTGGGCATGGAGCACTTGCGAGAGGTGAGGGCAGTTACCAG
TGCCAATATCCAGGAGTTTGCTGGCTGCAAGAAGATCTTTGGGAGCCTGGCATTTCT
GCCGGAGAGCTTTGATGGGGACCCAGCCTCCAACACTGCCCCGCTCCAGCCAGAGC
AGCTCCAAGTGTTTGAGACTCTGGAAGAGATCACAGGTTACCTATACATCTCAGCAT
GGCCGGACAGCCTGCCTGACCTCAGCGTCTTCCAGAACCTGCAAGTAATCCGGGGA
CGAATTCTGCACAATGGCGCCTACTCGCTGACCCTGCAAGGGCTGGGCATCAGCTG
GCTGGGGCTGCGCTCACTGAGGGAACTGGGCAGTGGACTGGCCCTCATCCACCATA
ACACCCACCTCTGCTTCGTGCACACGGTGCCCTGGGACCAGCTCTTTCGGAACCCGC
ACCAAGCTCTGCTCCACACTGCCAACCGGCCAGAGGACGAGTGTGTGGGCGAGGGC
CTGGCCTGCCACCAGCTGTGCGCCCGAGGGCACTGCTGGGGTCCAGGGCCCACCCA
GTGTGTCAACTGCAGCCAGTTCCTTCGGGGCCAGGAGTGCGTGGAGGAATGCCGAG
TACTGCAGGGGCTCCCCAGGGAGTATGTGAATGCCAGGCACTGTTTGCCGTGCCAC
CCTGAGTGTCAGCCCCAGAATGGCTCAGTGACCTGTTTTGGACCGGAGGCTGACCA
GTGTGTGGCCTGTGCCCACTATAAGGACCCTCCCTTCTGCGTGGCCCGCTGCCCCA
GCGGTGTGAAACCTGACCTCTCCTACATGCCCATCTGGAAGTTTCCAGATGAGGAG
GCGCATGCCAGCCTTGCCCCATCAACTGCACCCACTCCTGTGTGGACCTGGATGACA
AGGGCTGCCCCGCCGAGCAGAGAGCCAGCCCTCTGACGTCCATCATCTCTGCGGTG
GTTGGCATTCTGCTGGTCGTGGTCTTGGGGGTGGTCTTTGGGATCCTCATCAAGCGA
CGGCAGCAGAAGATCCGGAAGTActcgagcTATGCTGCGAAAGTCTTCTTGAGATGCAT
CAATTATGTCTTCTTTCCATCACTTAAACCTTCTTCCAGTATAGATGAGTATTTCTCT
GAACAGCCATTGAAGAATCTTCTGCTTTCAACTTCTGAGGAACAAATCGAAAAATGTT
TCATAATTGAAAATATAAGCACAATTGCTACAGTAGAAGAAACTAATCAAACTGATGA
AGATCATAAAAAATACAGTTCCCAAACTAGCCAAGATTCAGGAAATTATTCTAATGAA
GATGAAAGCGAAAGTAAAACAAGTgtcgactaa

⑤ HER2-EX-TM-IFNAR1-IN-D41

ATGGAGCTGGCGGCCTTGTGCCGCTGGGGGCTCCTCCTCGCCCTCTTGCCCCCCGG
AGCCGCGAGCACCCAAGTGTGCACCGGCACAGACATGAAGCTGCGGCTCCCTGCCA
GTCCCGAGACCCACCTGGACATGCTCCGCCACCTCTACCAGGGCTGCCAGGTGGTG
CAGGGAAACCTGGAACTCACCTACCTGCCCACCAATGCCAGCCTGTCCTTCCTGCAG
GATATCCAGGAGGTGCAGGGCTACGTGCTCATCGCTCACAACCAAGTGAGGCAGGT
CCCACTGCAGAGGCTGCGGATTGTGCGAGGCACCCAGCTCTTTGAGGACAACTATGC
CCTGGCCGTGCTAGACAATGGAGACCCGCTGAACAATACCACCCCTGTCACAGGGGC
CTCCCCAGGAGGCCTGCGGGAGCTGCAGCTTCGAAGCCTCACAGAGATCTTGAAAG
GAGGGGTCTTGATCCAGCGGAACCCCCAGCTCTGCTACCAGGACACGATTTTGTGGA
AGGACATCTTCCACAAGAACAACCAGCTGGCTCTCACACTGATAGACACCAACCGCT
CTCGGGCCTGCCACCCCTGTTCTCCGATGTGTAAGGGCTCCCGCTGCTGGGGAGAG
AGTTCTGAGGATTGTCAGAGCCTGACGCGCACTGTCTGTGCCGGTGGCTGTGCCCG
CTGCAAGGGGCCACTGCCCACTGACTGCTGCCATGAGCAGTGTGCTGCCGGCTGCA
CGGGCCCCAAGCACTCTGACTGCCTGGCCTGCCTCCACTTCAACCACAGTGGCATCT
GTGAGCTGCACTGCCCAGCCCTGGTCACCTACAACACAGACACGTTTGAGTCCATGC
CCAATCCCGAGGGCCGGTATACATTCGGCGCCAGCTGTGTGACTGCCTGTCCCTACA
ACTACCTTTCTACGGACGTGGGATCCTGCACCCTCGTCTGCCCCCTGCACAACCAAG
AGGTGACAGCAGAGGATGGAACACAGCGGTGTGAGAAGTGCAGCAAGCCCTGTGCC
CGAGTGTGCTATGGTCTGGGCATGGAGCACTTGCGAGAGGTGAGGGCAGTTACCAG
TGCCAATATCCAGGAGTTTGCTGGCTGCAAGAAGATCTTTGGGAGCCTGGCATTTCT
GCCGGAGAGCTTTGATGGGGACCCAGCCTCCAACACTGCCCCGCTCCAGCCAGAGC
AGCTCCAAGTGTTTGAGACTCTGGAAGAGATCACAGGTTACCTATACATCTCAGCAT
GGCCGGACAGCCTGCCTGACCTCAGCGTCTTCCAGAACCTGCAAGTAATCCGGGGA
CGAATTCTGCACAATGGCGCCTACTCGCTGACCCTGCAAGGGCTGGGCATCAGCTG
GCTGGGGCTGCGCTCACTGAGGGAACTGGGCAGTGGACTGGCCCTCATCCACCATA
ACACCCACCTCTGCTTCGTGCACACGGTGCCCTGGGACCAGCTCTTTCGGAACCCGC
ACCAAGCTCTGCTCCACACTGCCAACCGGCCAGAGGACGAGTGTGTGGGCGAGGGC
CTGGCCTGCCACCAGCTGTGCGCCCGAGGGCACTGCTGGGGTCCAGGGCCCACCCA
GTGTGTCAACTGCAGCCAGTTCCTTCGGGGCCAGGAGTGCGTGGAGGAATGCCGAG
TACTGCAGGGGCTCCCCAGGGAGTATGTGAATGCCAGGCACTGTTTGCCGTGCCAC

CCTGAGTGTCAGCCCCAGAATGGCTCAGTGACCTGTTTTGGACCGGAGGCTGACCA
GTGTGTGGCCTGTGCCCACTATAAGGACCCTCCCTTCTGCGTGGCCCGCTGCCCCA
GCGGTGTGAAACCTGACCTCTCCTACATGCCCATCTGGAAGTTTCCAGATGAGGAGG
GCGCATGCCAGCCTTGCCCCATCAACTGCACCCACTCCTGTGTGGACCTGGATGACA
AGGGCTGCCCCGCCGAGCAGAGAGCCAGCCCTCTGACGTCCATCATCTCTGCGGTG
GTTGGCATTCTGCTGGTCGTGGTCTTGGGGGTGGTCTTTGGGATCCTCATCAAGCGA
CGGCAGCAGAAGATCCGGAAGTActcgagcTATGCTGCGAAAGTCTTCTTGAGATGCAT
CAATTATGTCTTCTTTCCATCACTTAAACCTTCTTCCAGTATAGATGAGTATTTCTCT
GAACAGCCATTGAAGAATCTTCTGCTTTCAACTTCTGAGGAACAAATCGAAAAATGTT
TCATAATTGAAAATATAAGCACAATTGCTACAGTAtaa

⑥ HER2-EX-TM-IFNAR1-IN-D62

ATGGAGCTGGCGGCCTTGTGCCGCTGGGGGCTCCTCCTCGCCCTCTTGCCCCCCGG
AGCCGCGAGCACCCAAGTGTGCACCGGCACAGACATGAAGCTGCGGCTCCCTGCCA
GTCCCGAGACCCACCTGGACATGCTCCGCCACCTCTACCAGGGCTGCCAGGTGGTG
CAGGGAAACCTGGAACTCACCTACCTGCCCACCAATGCCAGCCTGTCCTTCCTGCAG
GATATCCAGGAGGTGCAGGGCTACGTGCTCATCGCTCACAACCAAGTGAGGCAGGT
CCCACTGCAGAGGCTGCGGATTGTGCGAGGCACCCAGCTCTTTGAGGACAACTATGC
CCTGGCCGTGCTAGACAATGGAGACCCGCTGAACAATACCACCCCTGTCACAGGGGC
CTCCCCAGGAGGCCTGCGGGAGCTGCAGCTTCGAAGCCTCACAGAGATCTTGAAAG
GAGGGGTCTTGATCCAGCGGAACCCCCAGCTCTGCTACCAGGACACGATTTTGTGGA
AGGACATCTTCCACAAGAACAACCAGCTGGCTCTCACACTGATAGACACCAACCGCT
CTCGGGCCTGCCACCCCTGTTCTCCGATGTGTAAGGGCTCCCGCTGCTGGGGAGAG
AGTTCTGAGGATTGTCAGAGCCTGACGCGCACTGTCTGTGCCGGTGGCTGTGCCCG
CTGCAAGGGGCCACTGCCCACTGACTGCTGCCATGAGCAGTGTGCTGCCGGCTGCA
CGGGCCCCAAGCACTCTGACTGCCTGGCCTGCCTCCACTTCAACCACAGTGGCATCT
GTGAGCTGCACTGCCCAGCCCTGGTCACCTACAACACAGACACGTTTGAGTCCATGC
CCAATCCCGAGGGCCGGTATACATTCGGCGCCAGCTGTGTGACTGCCTGTCCCTACA
ACTACCTTTCTACGGACGTGGGATCCTGCACCCTCGTCTGCCCCCTGCACAACCAAG
AGGTGACAGCAGAGGATGGAACACAGCGGTGTGAGAAGTGCAGCAAGCCCTGTGCC
CGAGTGTGCTATGGTCTGGGCATGGAGCACTTGCGAGAGGTGAGGGCAGTTACCAG
TGCCAATATCCAGGAGTTTGCTGGCTGCAAGAAGATCTTTGGGAGCCTGGCATTTCT
GCCGGAGAGCTTTGATGGGGACCCAGCCTCCAACACTGCCCCGCTCCAGCCAGAGC
AGCTCCAAGTGTTTGAGACTCTGGAAGAGATCACAGGTTACCTATACATCTCAGCAT
GGCCGGACAGCCTGCCTGACCTCAGCGTCTTCCAGAACCTGCAAGTAATCCGGGGA
CGAATTCTGCACAATGGCGCCTACTCGCTGACCCTGCAAGGGCTGGGCATCAGCTG
GCTGGGGCTGCGCTCACTGAGGGAACTGGGCAGTGGACTGGCCCTCATCCACCATA
ACACCCACCTCTGCTTCGTGCACACGGTGCCCTGGGACCAGCTCTTTCGGAACCCGC
ACCAAGCTCTGCTCCACACTGCCAACCGGCCAGAGGACGAGTGTGTGGGCGAGGGC
CTGGCCTGCCACCAGCTGTGCGCCCGAGGGCACTGCTGGGGTCCAGGGCCCACCCA
GTGTGTCAACTGCAGCCAGTTCCTTCGGGGCCAGGAGTGCGTGGAGGAATGCCGAG
TACTGCAGGGGCTCCCCAGGGAGTATGTGAATGCCAGGCACTGTTTGCCGTGCCAC
CCTGAGTGTCAGCCCCAGAATGGCTCAGTGACCTGTTTTGGACCGGAGGCTGACCA
GTGTGTGGCCTGTGCCCACTATAAGGACCCTCCCTTCTGCGTGGCCCGCTGCCCCA
GCGGTGTGAAACCTGACCTCTCCTACATGCCCATCTGGAAGTTTCCAGATGAGGAGG
GCGCATGCCAGCCTTGCCCCATCAACTGCACCCACTCCTGTGTGGACCTGGATGACA
AGGGCTGCCCCGCCGAGCAGAGAGCCAGCCCTCTGACGTCCATCATCTCTGCGGTG
GTTGGCATTCTGCTGGTCGTGGTCTTGGGGGTGGTCTTTGGGATCCTCATCAAGCGA
CGGCAGCAGAAGATCCGGAAGTActcgagcTTGAGATGCATCAATTATGTCTTCTTTCC
ATCACTTAAACCTTCTTCCAGTATAGATGAGTATTTCTCTGAACAGCCATTGAAGAAT
CTTCTGCTTTCAACTTCTGAGGAACAAATCGAAAAATGTTTCATAATTGAAAATATAA
GCACAATTGCTACAGTAGAAGAAACTAATCAAACTGATGAAGATCATAAAAAATACAG
TTCCCAAACTAGCCAAGATTCAGGAAATtaa

⑦ HER2-EX-TM-IFNAR1-IN-D61

ATGGAGCTGGCGGCCTTGTGCCGCTGGGGGCTCCTCCTCGCCCTCTTGCCCCCCGG
AGCCGCGAGCACCCAAGTGTGCACCGGCACAGACATGAAGCTGCGGCTCCCTGCCA
GTCCCGAGACCCACCTGGACATGCTCCGCCACCTCTACCAGGGCTGCCAGGTGGTG
CAGGGAAACCTGGAACTCACCTACCTGCCCACCAATGCCAGCCTGTCCTTCCTGCAG
GATATCCAGGAGGTGCAGGGCTACGTGCTCATCGCTCACAACCAAGTGAGGCAGGT
CCCACTGCAGAGGCTGCGGATTGTGCGAGGCACCCAGCTCTTTGAGGACAACTATGC
CCTGGCCGTGCTAGACAATGGAGACCCGCTGAACAATACCACCCCTGTCACAGGGGC
CTCCCCAGGAGGCCTGCGGGAGCTGCAGCTTCGAAGCCTCACAGAGATCTTGAAAG
GAGGGGTCTTGATCCAGCGGAACCCCCAGCTCTGCTACCAGGACACGATTTTGTGGA
AGGACATCTTCCACAAGAACAACCAGCTGGCTCTCACACTGATAGACACCAACCGCT
CTCGGGCCTGCCACCCCTGTTCTCCGATGTGTAAGGGCTCCCGCTGCTGGGGAGAG
AGTTCTGAGGATTGTCAGAGCCTGACGCGCACTGTCTGTGCCGGTGGCTGTGCCCG
CTGCAAGGGGCCACTGCCCACTGACTGCTGCCATGAGCAGTGTGCTGCCGGCTGCA
CGGGCCCCAAGCACTCTGACTGCCTGGCCTGCCTCCACTTCAACCACAGTGGCATCT
GTGAGCTGCACTGCCCAGCCCTGGTCACCTACAACACAGACACGTTTGAGTCCATGC
CCAATCCCGAGGGCCGGTATACATTCGGCGCCAGCTGTGTGACTGCCTGTCCCTACA
ACTACCTTTCTACGGACGTGGGATCCTGCACCCTCGTCTGCCCCCTGCACAACCAAG
AGGTGACAGCAGAGGATGGAACACAGCGGTGTGAGAAGTGCAGCAAGCCCTGTGCC
CGAGTGTGCTATGGTCTGGGCATGGAGCACTTGCGAGAGGTGAGGGCAGTTACCAG
TGCCAATATCCAGGAGTTTGCTGGCTGCAAGAAGATCTTTGGGAGCCTGGCATTTCT
GCCGGAGAGCTTTGATGGGGACCCAGCCTCCAACACTGCCCCGCTCCAGCCAGAGC
AGCTCCAAGTGTTTGAGACTCTGGAAGAGATCACAGGTTACCTATACATCTCAGCAT
GGCCGGACAGCCTGCCTGACCTCAGCGTCTTCCAGAACCTGCAAGTAATCCGGGGA
CGAATTCTGCACAATGGCGCCTACTCGCTGACCCTGCAAGGGCTGGGCATCAGCTG
GCTGGGGCTGCGCTCACTGAGGGAACTGGGCAGTGGACTGGCCCTCATCCACCATA
ACACCCACCTCTGCTTCGTGCACACGGTGCCCTGGGACCAGCTCTTTCGGAACCCGC
ACCAAGCTCTGCTCCACACTGCCAACCGGCCAGAGGACGAGTGTGTGGGCGAGGGC
CTGGCCTGCCACCAGCTGTGCGCCCGAGGGCACTGCTGGGGTCCAGGGCCCACCCA
GTGTGTCAACTGCAGCCAGTTCCTTCGGGGCCAGGAGTGCGTGGAGGAATGCCGAG
TACTGCAGGGGCTCCCCAGGGAGTATGTGAATGCCAGGCACTGTTTGCCGTGCCAC
CCTGAGTGTCAGCCCCAGAATGGCTCAGTGACCTGTTTTGGACCGGAGGCTGACCA
GTGTGTGGCCTGTGCCCACTATAAGGACCCTCCCTTCTGCGTGGCCCGCTGCCCCA
GCGGTGTGAAACCTGACCTCTCCTACATGCCCATCTGGAAGTTTCCAGATGAGGAG
GCGCATGCCAGCCTTGCCCCATCAACTGCACCCACTCCTGTGTGGACCTGGATGACA
AGGGCTGCCCCGCCGAGCAGAGAGCCAGCCCTCTGACGTCCATCATCTCTGCGGTG
GTTGGCATTCTGCTGGTCGTGGTCTTGGGGGTGGTCTTTGGGATCCTCATCAAGCGA
CGGCAGCAGAAGATCCGGAAGTActcgagcTTGAGATGCATCAATTATGTCTTCTTTCC
ATCACTTAAACCTTCTTCCAGTATAGATGAGTATTTCTCTGAACAGCCATTGAAGAAT
CTTCTGCTTTCAACTTCTGAGGAACAAATCGAAAAATGTTTCATAATTGAAAATATAA
GCACAATTGCTACAGTAtaa

⑧ HER2-EX-TM-IFNAR1-IN-D71

ATGGAGCTGGCGGCCTTGTGCCGCTGGGGGCTCCTCCTCGCCCTCTTGCCCCCCGG
AGCCGCGAGCACCCAAGTGTGCACCGGCACAGACATGAAGCTGCGGCTCCCTGCCA
GTCCCGAGACCCACCTGGACATGCTCCGCCACCTCTACCAGGGCTGCCAGGTGGTG
CAGGGAAACCTGGAACTCACCTACCTGCCCACCAATGCCAGCCTGTCCTTCCTGCAG
GATATCCAGGAGGTGCAGGGCTACGTGCTCATCGCTCACAACCAAGTGAGGCAGGT
CCCACTGCAGAGGCTGCGGATTGTGCGAGGCACCCAGCTCTTTGAGGACAACTATGC
CCTGGCCGTGCTAGACAATGGAGACCCGCTGAACAATACCACCCCTGTCACAGGGGC
CTCCCCAGGAGGCCTGCGGGAGCTGCAGCTTCGAAGCCTCACAGAGATCTTGAAAG
GAGGGGTCTTGATCCAGCGGAACCCCCAGCTCTGCTACCAGGACACGATTTTGTGGA
AGGACATCTTCCACAAGAACAACCAGCTGGCTCTCACACTGATAGACACCAACCGCT
CTCGGGCCTGCCACCCCTGTTCTCCGATGTGTAAGGGCTCCCGCTGCTGGGGAGAG

AGTTCTGAGGATTGTCAGAGCCTGACGCGCACTGTCTGTGCCGGTGGCTGTGCCCG
CTGCAAGGGGCCACTGCCCACTGACTGCTGCCATGAGCAGTGTGCTGCCGGCTGCA
CGGGCCCCAAGCACTCTGACTGCCTGGCCTGCCTCCACTTCAACCACAGTGGCATCT
GTGAGCTGCACTGCCCAGCCCTGGTCACCTACAACACAGACACGTTTGAGTCCATGC
CCAATCCCGAGGGCCGGTATACATTCGGCGCCAGCTGTGTGACTGCCTGTCCCTACA
ACTACCTTTCTACGGACGTGGGATCCTGCACCCTCGTCTGCCCCCTGCACAACCAAG
AGGTGACAGCAGAGGATGGAACACAGCGGTGTGAGAAGTGCAGCAAGCCCTGTGCC
CGAGTGTGCTATGGTCTGGGCATGGAGCACTTGCGAGAGGTGAGGGCAGTTACCAG
TGCCAATATCCAGGAGTTTGCTGGCTGCAAGAAGATCTTTGGGAGCCTGGCATTTCT
GCCGGAGAGCTTTGATGGGGACCCAGCCTCCAACACTGCCCCGCTCCAGCCAGAGC
AGCTCCAAGTGTTTGAGACTCTGGAAGAGATCACAGGTTACCTATACATCTCAGCAT
GGCCGGACAGCCTGCCTGACCTCAGCGTCTTCCAGAACCTGCAAGTAATCCGGGGA
CGAATTCTGCACAATGGCGCCTACTCGCTGACCCTGCAAGGGCTGGGCATCAGCTG
GCTGGGGCTGCGCTCACTGAGGGAACTGGGCAGTGGACTGGCCCTCATCCACCATA
ACACCCACCTCTGCTTCGTGCACACGGTGCCCTGGGACCAGCTCTTTCGGAACCCGC
ACCAAGCTCTGCTCCACACTGCCAACCGGCCAGAGGACGAGTGTGTGGGCGAGGGC
CTGGCCTGCCACCAGCTGTGCGCCCGAGGGCACTGCTGGGGTCCAGGGCCCACCCA
GTGTGTCAACTGCAGCCAGTTCCTTCGGGGCCAGGAGTGCGTGGAGGAATGCCGAG
TACTGCAGGGGCTCCCCAGGGAGTATGTGAATGCCAGGCACTGTTTGCCGTGCCAC
CCTGAGTGTCAGCCCCAGAATGGCTCAGTGACCTGTTTTGGACCGGAGGCTGACCA
GTGTGTGGCCTGTGCCCACTATAAGGACCCTCCCTTCTGCGTGGCCCGCTGCCCCA
GCGGTGTGAAACCTGACCTCTCCTACATGCCCATCTGGAAGTTTCCAGATGAGGAG
GCGCATGCCAGCCTTGCCCCATCAACTGCACCCACTCCTGTGTGGACCTGGATGACA
AGGGCTGCCCCGCCGAGCAGAGAGCCAGCCCTCTGACGTCCATCATCTCTGCGGTG
GTTGGCATTCTGCTGGTCGTGGTCTTGGGGGTGGTCTTTGGGATCCTCATCAAGCGA
CGGCAGCAGAAGATCCGGAAGTActcgagcTATGTCTTCTTTCCATCACTTAAACCTTC
TTCCAGTATAGATGAGTATTTCTCTGAACAGCCATTGAAGAATCTTCTGCTTTCAACT
TCTGAGGAACAAATCGAAAAATGTTTCATAATTGAAAATATAAGCACAATTGCTACAG
TAtaa

Supplementary Table 6

DNA sequences in the plasmid constructs of Supplementary Fig 6.

anti-HER2-Af Dimer

ATGAACTCCTTCTCCACAAGCGCCTTCGGTCCAGTTGCCTTCTCCCTGGGCCTGCTC
CTGGTGTTGCCTGCTGCCTTCCCTGCCCCAgtcccctccggaggtggagggtcgtcaggtGTGG
ACAATAAATTCAACAAAGAAATGAGAAACGCCTACTGGGAGATCGCCCTGCTACCCA
ACCTGAACAACCAACAGAAGAGAGCGTTTATCAGAAGCCTGTACGACGACCCTAGCC
AATCTGCTAACCTGCTGGCAGAGGCTAAGAAACTGAATGACGCCCAAGCCCCTAAGg
gcggtggaggctcgaccATGGCCGGAACTACCGTGGACAATAAATTCAACAAAGAAATGAG
AAACGCCTACTGGGAGATCGCCCTGCTACCCAACCTGAACAACCAACAGAAGAGAGC
GTTTATCAGAAGCCTGTACGACGACCCTAGCCAATCTGCTAACCTGCTGGCAGAGGC
TAAGAAACTGAATGACGCCCAAGCCCCTAAGggcggccgctaa

① HER2-EX-TM-SmBiT

ATGGAGCTGGCGGCCTTGTGCCGCTGGGGGCTCCTCCTCGCCCTCTTGCCCCCCGG
AGCCGCGAGCACCCAAGTGTGCACCGGCACAGACATGAAGCTGCGGCTCCCTGCCA
GTCCCGAGACCCACCTGGACATGCTCCGCCACCTCTACCAGGGCTGCCAGGTGGTG
CAGGGAAACCTGGAACTCACCTACCTGCCCACCAATGCCAGCCTGTCCTTCCTGCAG
GATATCCAGGAGGTGCAGGGCTACGTGCTCATCGCTCACAACCAAGTGAGGCAGGT
CCCACTGCAGAGGCTGCGGATTGTGCGAGGCACCCAGCTCTTTGAGGACAACTATGC

CCTGGCCGTGCTAGACAATGGAGACCCGCTGAACAATACCACCCCTGTCACAGGGGC
CTCCCCAGGAGGCCTGCGGGAGCTGCAGCTTCGAAGCCTCACAGAGATCTTGAAAG
GAGGGGTCTTGATCCAGCGGAACCCCCAGCTCTGCTACCAGGACACGATTTTGTGGA
AGGACATCTTCCACAAGAACAACCAGCTGGCTCTCACACTGATAGACACCAACCGCT
CTCGGGCCTGCCACCCCTGTTCTCCGATGTGTAAGGGCTCCCGCTGCTGGGGAGAG
AGTTCTGAGGATTGTCAGAGCCTGACGCGCACTGTCTGTGCCGGTGGCTGTGCCCG
CTGCAAGGGGCCACTGCCCACTGACTGCTGCCATGAGCAGTGTGCTGCCGGCTGCA
CGGGCCCCAAGCACTCTGACTGCCTGGCCTGCCTCCACTTCAACCACAGTGGCATCT
GTGAGCTGCACTGCCCAGCCCTGGTCACCTACAACACAGACACGTTTGAGTCCATGC
CCAATCCCGAGGGCCGGTATACATTCGGCGCCAGCTGTGTGACTGCCTGTCCCTACA
ACTACCTTTCTACGGACGTGGGATCCTGCACCCTCGTCTGCCCCCTGCACAACCAAG
AGGTGACAGCAGAGGATGGAACACAGCGGTGTGAGAAGTGCAGCAAGCCCTGTGCC
CGAGTGTGCTATGGTCTGGGCATGGAGCACTTGCGAGAGGTGAGGGCAGTTACCAG
TGCCAATATCCAGGAGTTTGCTGGCTGCAAGAAGATCTTTGGGAGCCTGGCATTTCT
GCCGGAGAGCTTTGATGGGGACCCAGCCTCCAACACTGCCCCGCTCCAGCCAGAGC
AGCTCCAAGTGTTTGAGACTCTGGAAGAGATCACAGGTTACCTATACATCTCAGCAT
GGCCGGACAGCCTGCCTGACCTCAGCGTCTTCCAGAACCTGCAAGTAATCCGGGGA
CGAATTCTGCACAATGGCGCCTACTCGCTGACCCTGCAAGGGCTGGGCATCAGCTG
GCTGGGGCTGCGCTCACTGAGGGAACTGGGCAGTGGACTGGCCCTCATCCACCATA
ACACCCACCTCTGCTTCGTGCACACGGTGCCCTGGGACCAGCTCTTTCGGAACCCGC
ACCAAGCTCTGCTCCACACTGCCAACCGGCCAGAGGACGAGTGTGTGGGCGAGGGC
CTGGCCTGCCACCAGCTGTGCGCCCGAGGGCACTGCTGGGGTCCAGGGCCCACCCA
GTGTGTCAACTGCAGCCAGTTCCTTCGGGGCCAGGAGTGCGTGGAGGAATGCCGAG
TACTGCAGGGGCTCCCCAGGGAGTATGTGAATGCCAGGCACTGTTTGCCGTGCCAC
CCTGAGTGTCAGCCCCAGAATGGCTCAGTGACCTGTTTTGGACCGGAGGCTGACCA
GTGTGTGGCCTGTGCCCACTATAAGGACCCTCCCTTCTGCGTGGCCCGCTGCCCCA
GCGGTGTGAAACCTGACCTCTCCTACATGCCCATCTGGAAGTTTCCAGATGAGGAG
GCGCATGCCAGCCTTGCCCCATCAACTGCACCCACTCCTGTGTGGACCTGGATGACA
AGGGCTGCCCCGCCGAGCAGAGAGCCAGCCCTCTGACGTCCATCATCTCTGCGGTG
GTTGGCATTCTGCTGGTCGTGGTCTTGGGGGTGGTCTTTGGGATCCTCATCAAGCGA
CGGCAGCAGAAGATCCGGAAGTActcgagcggtGTGACCGGCTACCGGCTGTTCGAGGA
GATTCTGtaa

② HER2-EX-TM-Linker-SmBiT

ATGGAGCTGGCGGCCTTGTGCCGCTGGGGGCTCCTCCTCGCCCTCTTGCCCCCCGG
AGCCGCGAGCACCCAAGTGTGCACCGGCACAGACATGAAGCTGCGGCTCCCTGCCA
GTCCCGAGACCCACCTGGACATGCTCCGCCACCTCTACCAGGGCTGCCAGGTGGTG
CAGGGAAACCTGGAACTCACCTACCTGCCCACCAATGCCAGCCTGTCCTTCCTGCAG
GATATCCAGGAGGTGCAGGGCTACGTGCTCATCGCTCACAACCAAGTGAGGCAGGT
CCCACTGCAGAGGCTGCGGATTGTGCGAGGCACCCAGCTCTTTGAGGACAACTATGC
CCTGGCCGTGCTAGACAATGGAGACCCGCTGAACAATACCACCCCTGTCACAGGGGC
CTCCCCAGGAGGCCTGCGGGAGCTGCAGCTTCGAAGCCTCACAGAGATCTTGAAAG
GAGGGGTCTTGATCCAGCGGAACCCCCAGCTCTGCTACCAGGACACGATTTTGTGGA
AGGACATCTTCCACAAGAACAACCAGCTGGCTCTCACACTGATAGACACCAACCGCT
CTCGGGCCTGCCACCCCTGTTCTCCGATGTGTAAGGGCTCCCGCTGCTGGGGAGAG
AGTTCTGAGGATTGTCAGAGCCTGACGCGCACTGTCTGTGCCGGTGGCTGTGCCCG
CTGCAAGGGGCCACTGCCCACTGACTGCTGCCATGAGCAGTGTGCTGCCGGCTGCA
CGGGCCCCAAGCACTCTGACTGCCTGGCCTGCCTCCACTTCAACCACAGTGGCATCT
GTGAGCTGCACTGCCCAGCCCTGGTCACCTACAACACAGACACGTTTGAGTCCATGC
CCAATCCCGAGGGCCGGTATACATTCGGCGCCAGCTGTGTGACTGCCTGTCCCTACA
ACTACCTTTCTACGGACGTGGGATCCTGCACCCTCGTCTGCCCCCTGCACAACCAAG
AGGTGACAGCAGAGGATGGAACACAGCGGTGTGAGAAGTGCAGCAAGCCCTGTGCC
CGAGTGTGCTATGGTCTGGGCATGGAGCACTTGCGAGAGGTGAGGGCAGTTACCAG
TGCCAATATCCAGGAGTTTGCTGGCTGCAAGAAGATCTTTGGGAGCCTGGCATTTCT
GCCGGAGAGCTTTGATGGGGACCCAGCCTCCAACACTGCCCCGCTCCAGCCAGAGC
AGCTCCAAGTGTTTGAGACTCTGGAAGAGATCACAGGTTACCTATACATCTCAGCAT

GGCCGGACAGCCTGCCTGACCTCAGCGTCTTCCAGAACCTGCAAGTAATCCGGGGA
CGAATTCTGCACAATGGCGCCTACTCGCTGACCCTGCAAGGGCTGGGCATCAGCTG
GCTGGGGCTGCGCTCACTGAGGGAACTGGGCAGTGGACTGGCCCTCATCCACCATA
ACACCCACCTCTGCTTCGTGCACACGGTGCCCTGGGACCAGCTCTTTCGGAACCCGC
ACCAAGCTCTGCTCCACACTGCCAACCGGCCAGAGGACGAGTGTGTGGGCGAGGGC
CTGGCCTGCCACCAGCTGTGCGCCCGAGGGCACTGCTGGGGTCCAGGGCCCACCCA
GTGTGTCAACTGCAGCCAGTTCCTTCGGGGCCAGGAGTGCGTGGAGGAATGCCGAG
TACTGCAGGGGCTCCCCAGGGAGTATGTGAATGCCAGGCACTGTTTGCCGTGCCAC
CCTGAGTGTCAGCCCCAGAATGGCTCAGTGACCTGTTTTGGACCGGAGGCTGACCA
GTGTGTGGCCTGTGCCCACTATAAGGACCCTCCCTTCTGCGTGGCCCGCTGCCCCA
GCGGTGTGAAACCTGACCTCTCCTACATGCCCATCTGGAAGTTTCCAGATGAGGAG
GCGCATGCCAGCCTTGCCCCATCAACTGCACCCACTCCTGTGTGGACCTGGATGACA
AGGGCTGCCCCGCCGAGCAGAGAGCCAGCCCTCTGACGTCCATCATCTCTGCGGTG
GTTGGCATTCTGCTGGTCGTGGTCTTGGGGGTGGTCTTTGGGATCCTCATCAAGCGA
CGGCAGCAGAAGATCCGGAAGTActcgagcGGTGGTGGAGGGTCAGGAGGTGGAGGGt
cgagcGGTGGTGGAGGGTCAGGAGGTGGAGGGtcgagcGGTGGTGGAGGGTCAGGAGG
TGGAGGGtcgagcgccATGGGCTCCGGAGGCGGATCCGGTGGGtcgagcggtGTGACCGG
CTACCGGCTGTTCGAGGAGATTCTGTAA

③ HER2-EX-TM-LgBiT

ATGGAGCTGGCGGCCTTGTGCCGCTGGGGGCTCCTCCTCGCCCTCTTGCCCCCCGG
AGCCGCGAGCACCCAAGTGTGCACCGGCACAGACATGAAGCTGCGGCTCCCTGCCA
GTCCCGAGACCCACCTGGACATGCTCCGCCACCTCTACCAGGGCTGCCAGGTGGTG
CAGGGAAACCTGGAACTCACCTACCTGCCCACCAATGCCAGCCTGTCCTTCCTGCAG
GATATCCAGGAGGTGCAGGGCTACGTGCTCATCGCTCACAACCAAGTGAGGCAGGT
CCCACTGCAGAGGCTGCGGATTGTGCGAGGCACCCAGCTCTTTGAGGACAACTATGC
CCTGGCCGTGCTAGACAATGGAGACCCGCTGAACAATACCACCCCTGTCACAGGGGC
CTCCCCAGGAGGCCTGCGGGAGCTGCAGCTTCGAAGCCTCACAGAGATCTTGAAAG
GAGGGGTCTTGATCCAGCGGAACCCCCAGCTCTGCTACCAGGACACGATTTTGTGGA
AGGACATCTTCCACAAGAACAACCAGCTGGCTCTCACACTGATAGACACCAACCGCT
CTCGGGCCTGCCACCCCTGTTCTCCGATGTGTAAGGGCTCCCGCTGCTGGGGAGAG
AGTTCTGAGGATTGTCAGAGCCTGACGCGCACTGTCTGTGCCGGTGGCTGTGCCCG
CTGCAAGGGGCCACTGCCCACTGACTGCTGCCATGAGCAGTGTGCTGCCGGCTGCA
CGGGCCCCAAGCACTCTGACTGCCTGGCCTGCCTCCACTTCAACCACAGTGGCATCT
GTGAGCTGCACTGCCCAGCCCTGGTCACCTACAACACAGACACGTTTGAGTCCATGC
CCAATCCCGAGGGCCGGTATACATTCGGCGCCAGCTGTGTGACTGCCTGTCCCTACA
ACTACCTTTCTACGGACGTGGGATCCTGCACCCTCGTCTGCCCCCTGCACAACCAAG
AGGTGACAGCAGAGGATGGAACACAGCGGTGTGAGAAGTGCAGCAAGCCCTGTGCC
CGAGTGTGCTATGGTCTGGGCATGGAGCACTTGCGAGAGGTGAGGGCAGTTACCAG
TGCCAATATCCAGGAGTTTGCTGGCTGCAAGAAGATCTTTGGGAGCCTGGCATTTCT
GCCGGAGAGCTTTGATGGGGACCCAGCCTCCAACACTGCCCCGCTCCAGCCAGAGC
AGCTCCAAGTGTTTGAGACTCTGGAAGAGATCACAGGTTACCTATACATCTCAGCAT
GGCCGGACAGCCTGCCTGACCTCAGCGTCTTCCAGAACCTGCAAGTAATCCGGGGA
CGAATTCTGCACAATGGCGCCTACTCGCTGACCCTGCAAGGGCTGGGCATCAGCTG
GCTGGGGCTGCGCTCACTGAGGGAACTGGGCAGTGGACTGGCCCTCATCCACCATA
ACACCCACCTCTGCTTCGTGCACACGGTGCCCTGGGACCAGCTCTTTCGGAACCCGC
ACCAAGCTCTGCTCCACACTGCCAACCGGCCAGAGGACGAGTGTGTGGGCGAGGGC
CTGGCCTGCCACCAGCTGTGCGCCCGAGGGCACTGCTGGGGTCCAGGGCCCACCCA
GTGTGTCAACTGCAGCCAGTTCCTTCGGGGCCAGGAGTGCGTGGAGGAATGCCGAG
TACTGCAGGGGCTCCCCAGGGAGTATGTGAATGCCAGGCACTGTTTGCCGTGCCAC
CCTGAGTGTCAGCCCCAGAATGGCTCAGTGACCTGTTTTGGACCGGAGGCTGACCA
GTGTGTGGCCTGTGCCCACTATAAGGACCCTCCCTTCTGCGTGGCCCGCTGCCCCA
GCGGTGTGAAACCTGACCTCTCCTACATGCCCATCTGGAAGTTTCCAGATGAGGAG
GCGCATGCCAGCCTTGCCCCATCAACTGCACCCACTCCTGTGTGGACCTGGATGACA
AGGGCTGCCCCGCCGAGCAGAGAGCCAGCCCTCTGACGTCCATCATCTCTGCGGTG
GTTGGCATTCTGCTGGTCGTGGTCTTGGGGGTGGTCTTTGGGATCCTCATCAAGCGA

CGGCAGCAGAAGATCCGGAAGTActcgagcggcGTCTTCACACTCGAAGATTTCGTTGG
GGACTGGGAACAGACAGCCGCCTACAACCTGGACCAAGTCCTTGAACAGGGAGGTG
TGTCCAGTTTGCTGCAGAATCTCGCCGTGTCCGTAACTCCGATCCAAAGGATTGTCC
GTAGCGGTGAAAATGCCCTGAAGATCGACATCCATGTCATCATCCCGTATGAAGGTC
TGAGCGCCGACCAAATGGCCCAGATCGAAGAGGTGTTTAAGGTGGTGTACCCTGTG
GATGATCATCACTTTAAGGTGATCCTGCCCTATGGCACACTGGTAATCGACGGGGTT
ACGCCGAACATGCTGAACTATTTCGGACGGCCGTATGAAGGCATCGCCGTGTTCGAC
GGCAAAAAGATCACTGTAACAGGGACCCTGTGGAACGGCAACAAAATTATCGACGAG
CGCCTGATCACCCCCGACGGCTCCATGCTGTTCCGAGTAACCATCAACAGTTAA

④　HER2-EX-TM-Linker-LgBiT

ATGGAGCTGGCGGCCTTGTGCCGCTGGGGGCTCCTCCTCGCCCTCTTGCCCCCCGG
AGCCGCGAGCACCCAAGTGTGCACCGGCACAGACATGAAGCTGCGGCTCCCTGCCA
GTCCCGAGACCCACCTGGACATGCTCCGCCACCTCTACCAGGGCTGCCAGGTGGTG
CAGGGAAACCTGGAACTCACCTACCTGCCCACCAATGCCAGCCTGTCCTTCCTGCAG
GATATCCAGGAGGTGCAGGGCTACGTGCTCATCGCTCACAACCAAGTGAGGCAGGT
CCCACTGCAGAGGCTGCGGATTGTGCGAGGCACCCAGCTCTTTGAGGACAACTATGC
CCTGGCCGTGCTAGACAATGGAGACCCGCTGAACAATACCACCCCTGTCACAGGGGC
CTCCCCAGGAGGCCTGCGGGAGCTGCAGCTTCGAAGCCTCACAGAGATCTTGAAAG
GAGGGGTCTTGATCCAGCGGAACCCCCAGCTCTGCTACCAGGACACGATTTTGTGGA
AGGACATCTTCCACAAGAACAACCAGCTGGCTCTCACACTGATAGACACCAACCGCT
CTCGGGCCTGCCACCCCTGTTCTCCGATGTGTAAGGGCTCCCGCTGCTGGGGAGAG
AGTTCTGAGGATTGTCAGAGCCTGACGCGCACTGTCTGTGCCGGTGGCTGTGCCCG
CTGCAAGGGGCCACTGCCCACTGACTGCTGCCATGAGCAGTGTGCTGCCGGCTGCA
CGGGCCCCAAGCACTCTGACTGCCTGGCCTGCCTCCACTTCAACCACAGTGGCATCT
GTGAGCTGCACTGCCCAGCCCTGGTCACCTACAACACAGACACGTTTGAGTCCATGC
CCAATCCCGAGGGCCGGTATACATTCGGCGCCAGCTGTGTGACTGCCTGTCCCTACA
ACTACCTTTCTACGGACGTGGGATCCTGCACCCTCGTCTGCCCCCTGCACAACCAAG
AGGTGACAGCAGAGGATGGAACACAGCGGTGTGAGAAGTGCAGCAAGCCCTGTGCC
CGAGTGTGCTATGGTCTGGGCATGGAGCACTTGCGAGAGGTGAGGGCAGTTACCAG
TGCCAATATCCAGGAGTTTGCTGGCTGCAAGAAGATCTTTGGGAGCCTGGCATTTCT
GCCGGAGAGCTTTGATGGGGACCCAGCCTCCAACACTGCCCCGCTCCAGCCAGAGC
AGCTCCAAGTGTTTGAGACTCTGGAAGAGATCACAGGTTACCTATACATCTCAGCAT
GGCCGGACAGCCTGCCTGACCTCAGCGTCTTCCAGAACCTGCAAGTAATCCGGGGA
CGAATTCTGCACAATGGCGCCTACTCGCTGACCCTGCAAGGGCTGGGCATCAGCTG
GCTGGGGCTGCGCTCACTGAGGGAACTGGGCAGTGGACTGGCCCTCATCCACCATA
ACACCCACCTCTGCTTCGTGCACACGGTGCCCTGGGACCAGCTCTTTCGGAACCCGC
ACCAAGCTCTGCTCCACACTGCCAACCGGCCAGAGGACGAGTGTGTGGGCGAGGGC
CTGGCCTGCCACCAGCTGTGCGCCCGAGGGCACTGCTGGGGTCCAGGGCCCACCCA
GTGTGTCAACTGCAGCCAGTTCCTTCGGGGCCAGGAGTGCGTGGAGGAATGCCGAG
TACTGCAGGGGCTCCCCAGGGAGTATGTGAATGCCAGGCACTGTTTGCCGTGCCAC
CCTGAGTGTCAGCCCCAGAATGGCTCAGTGACCTGTTTTGGACCGGAGGCTGACCA
GTGTGTGGCCTGTGCCCACTATAAGGACCCTCCCTTCTGCGTGGCCCGCTGCCCCA
GCGGTGTGAAACCTGACCTCTCCTACATGCCCATCTGGAAGTTTCCAGATGAGGAG
GCGCATGCCAGCCTTGCCCCATCAACTGCACCCACTCCTGTGTGGACCTGGATGACA
AGGGCTGCCCCGCCGAGCAGAGAGCCAGCCCTCTGACGTCCATCATCTCTGCGGTG
GTTGGCATTCTGCTGGTCGTGGTCTTGGGGGTGGTCTTTGGGATCCTCATCAAGCGA
CGGCAGCAGAAGATCCGGAAGTActcgagcGGTGGTGGAGGGTCAGGAGGTGGAGGGt
cgagcGGTGGTGGAGGGTCAGGAGGTGGAGGGtcgagcGGTGGTGGAGGGTCAGGAGG
TGGAGGGtcgagcgccATGGCCAGATCTGGGtcgagcggcGTCTTCACACTCGAAGATTTC
GTTGGGGACTGGGAACAGACAGCCGCCTACAACCTGGACCAAGTCCTTGAACAGGG
AGGTGTGTCCAGTTTGCTGCAGAATCTCGCCGTGTCCGTAACTCCGATCCAAAGGAT
TGTCCGTAGCGGTGAAAATGCCCTGAAGATCGACATCCATGTCATCATCCCGTATGA
AGGTCTGAGCGCCGACCAAATGGCCCAGATCGAAGAGGTGTTTAAGGTGGTGTACC
CTGTGGATGATCATCACTTTAAGGTGATCCTGCCCTATGGCACACTGGTAATCGACG
GGGTTACGCCGAACATGCTGAACTATTTCGGACGGCCGTATGAAGGCATCGCCGTGT

TCGACGGCAAAAAGATCACTGTAACAGGGACCCTGTGGAACGGCAACAAAATTATCG
ACGAGCGCCTGATCACCCCCGACGGCTCCATGCTGTTCCGAGTAACCATCAACAGTT
AA

Supplementary Table 7

DNA sequences in the plasmid constructs of Supplementary Fig 8.

① IFNGR1-EX1-IFNAR2-TM-IN-231

ATGGCTCTCCTCTTTCTCCTACCCCTTGTCATGCAGGGTGTGAGCAGGGCTGAGATG
GGCACCGCGGATCTGGGGCCGTCCTCAGTGCCTACACCAACTAATGTTACAATTGAA
TCCTATAACATGAACCCTATCGTATATTGGGAGTACCAGATCATGCCACAGGTCCCT
GTTTTTACCGTAGAGGTAAAGAACTATGGTGTTAAGAATTCAGAATGGATTGATGCCT
GCATCAATATTTCTCATCATTATTGTAATATTTCTGATCATGTTGGTGATCCATCAAA
TTCTCTTTGGGTCAGAGTTAAAGCCAGGGTTGGACAAAAAGAATCTGCCTATGCAAA
GTCAGAAGAATTTGCTGTATGCCGAGATGGAAAAATTGGACCACCTAAACTGGATAT
CAGAAAGGAGGAGAAGCAAATCATGATTGACATATTTCACCCTTCAGTTTTTGTAAAT
GGAGACGAGCAGGAAGTCGATTATGATCCCGAAACTACCTGTTACATTAGGGTGTAC
AATGTGTATGTGAGAATGAACGGAAGTGAGATCCAGTATAAAATACTCACGCAGAAG
GAAGATGATTGTGACGAGATTCAGTGCCAGTTAGCGATTCCAGTATCCTCACTGAAT
TCTCAGTACTGTGTTTCAGCAGAAGGAGTCTTACATGTGTGGGGTGTctcgagtCCACC
TGGCCAGGAATCAGAATCAGCAGAATCTGCCAAAATAGGAGGAATAATTACTGTGTT
TTTGATAGCATTGGTCTTGACAAGCACCATAGTGACACTGAAATGGATTGGTTATATA
TGCTTAAGAAATAGCCTCCCCAAAGTCTTGAATTTTCATAACTTTTTAGCCTGGCCAT
TTCCTAACCTGCCACCGTTGGAAGCCATGGATATGGTGGAGGTCATTTACATCAACA
GAAAGAAGAAAGTGTGGGATTATAATTATGATGATGAAAGTGATAGCGATACTGAGG
CAGCGCCCAGGACAAGTGGCGGTGGCTATACCATGCATGGACTGACTGTCAGGCCT
CTGGGTCAGGCCTCTGCCACCTCTACAGAATCCCAGTTGATAGACCCGGAGTCCGAG
GAGGAGCCTGACCTGCCTGAGGTTGATGTGGAGCTCCCCACGATGCCAAAGGACAG
CCCTCAGCAGTTGGAACTCTTGAGTGGGCCCTGTGAGAGGAGAAAGAGTCCACTCCA
GGACCCTTTTCCCGAAGAGGACTACAGCTCCACGGAGGGGTCTGGGGGCAGAATTA
CCTTCAATGTGGACTTAAACTCTGTGTTTTTGAGAGTTCTTGATGACGAGGACAGTG
ACGACTTAGAAGCCCCTCTGATGCTATCGTCTCATCTGGAAGAGATGGTTGACCCAG
AGGATCCTGATAATGTGCAATCAAACCATTTGCTGGCCAGCGGGGAAGGGACACAGC
CAACCTTTCCCAGCCCCTCTTCAGAGGGCCTGTGGTCCGAAGATGCTCCATCTGATC
AAAGTGACACTTCTGAGTCAGATGTTGACCTTGGGGATGGTTATATAATGAGATGA

② IFNGR1-EX2-IFNAR2-TM-IN-231

ATGGCTCTCCTCTTTCTCCTACCCCTTGTCATGCAGGGTGTGAGCAGGGCTGAGATG
GGCACCGCGGATCTGGGGCCGTCCTCAGTGCCTACACCAACTAATGTTACAATTGAA
TCCTATAACATGAACCCTATCGTATATTGGGAGTACCAGATCATGCCACAGGTCCCT
GTTTTTACCGTAGAGGTAAAGAACTATGGTGTTAAGAATTCAGAATGGATTGATGCCT
GCATCAATATTTCTCATCATTATTGTAATATTTCTGATCATGTTGGTGATCCATCAAA
TTCTCTTTGGGTCAGAGTTAAAGCCAGGGTTGGACAAAAAGAATCTGCCTATGCAAA
GTCAGAAGAATTTGCTGTATGCCGAGATGGAAAAATTGGACCACCTAAACTGGATAT
CAGAAAGGAGGAGAAGCAAATCATGATTGACATATTTCACCCTTCAGTTTTTGTAAAT
GGAGACGAGCAGGAAGTCGATTATGATCCCGAAACTACCTGTTACATTAGGGTGTAC
AATGTGTATGTGAGAATGAACGGAAGTGAGATCCAGTATAAAATACTCACGCAGAAG
GAAGATGATTGTGACGAGATTCAGTGCCAGTTAGCGATTCCAGTATCCTCACTGAAT
TCTCAGTACTGTGTTTCAGCAGAAGGAGTCTTACATGTGTGGGGTGTTACAACTGAA
AAGTCAAAAGAAGTctcgagtCCACCTGGCCAGGAATCAGAATCAGCAGAATCTGCCAA
AATAGGAGGAATAATTACTGTGTTTTTGATAGCATTGGTCTTGACAAGCACCATAGTG
ACACTGAAATGGATTGGTTATATATGCTTAAGAAATAGCCTCCCCAAAGTCTTGAATT

TTCATAACTTTTTAGCCTGGCCATTTCCTAACCTGCCACCGTTGGAAGCCATGGATAT
GGTGGAGGTCATTTACATCAACAGAAAGAAGAAAGTGTGGGATTATAATTATGATGA
TGAAAGTGATAGCGATACTGAGGCAGCGCCCAGGACAAGTGGCGGTGGCTATACCAT
GCATGGACTGACTGTCAGGCCTCTGGGTCAGGCCTCTGCCACCTCTACAGAATCCCA
GTTGATAGACCCGGAGTCCGAGGAGGAGCCTGACCTGCCTGAGGTTGATGTGGAGC
TCCCCACGATGCCAAAGGACAGCCCTCAGCAGTTGGAACTCTTGAGTGGGCCCTGTG
AGAGGAGAAAGAGTCCACTCCAGGACCCTTTTCCCGAAGAGGACTACAGCTCCACGG
AGGGGTCTGGGGGCAGAATTACCTTCAATGTGGACTTAAACTCTGTGTTTTTGAGAG
TTCTTGATGACGAGGACAGTGACGACTTAGAAGCCCCTCTGATGCTATCGTCTCATC
TGGAAGAGATGGTTGACCCAGAGGATCCTGATAATGTGCAATCAAACCATTTGCTGG
CCAGCGGGGAAGGGACACAGCCAACCTTTCCCAGCCCCTCTTCAGAGGGCCTGTGG
TCCGAAGATGCTCCATCTGATCAAAGTGACACTTCTGAGTCAGATGTTGACCTTGGG
GATGGTTATATAATGAGATGA

③ IFNGR1-EX3-IFNAR2-TM-IN-231

ATGGCTCTCCTCTTTCTCCTACCCCTTGTCATGCAGGGTGTGAGCAGGGCTGAGATG
GGCACCGCGGATCTGGGGCCGTCCTCAGTGCCTACACCAACTAATGTTACAATTGAA
TCCTATAACATGAACCCTATCGTATATTGGGAGTACCAGATCATGCCACAGGTCCCT
GTTTTTACCGTAGAGGTAAAGAACTATGGTGTTAAGAATTCAGAATGGATTGATGCCT
GCATCAATATTTCTCATCATTATTGTAATATTTCTGATCATGTTGGTGATCCATCAAA
TTCTCTTTGGGTCAGAGTTAAAGCCAGGGTTGGACAAAAAGAATCTGCCTATGCAAA
GTCAGAAGAATTTGCTGTATGCCGAGATGGAAAAATTGGACCACCTAAACTGGATAT
CAGAAAGGAGGAGAAGCAAATCATGATTGACATATTTCACCCTTCAGTTTTTGTAAAT
GGAGACGAGCAGGAAGTCGATTATGATCCCGAAACTACCTGTTACATTAGGGTGTAC
AATGTGTATGTGAGAATGAACGGAAGTGAGATCCAGTATAAAATACTCACGCAGAAG
GAAGATGATTGTGACGAGATTCAGTGCCAGTTAGCGATTCCAGTATCCTCACTGAAT
TCTCAGTACTGTGTTTCAGCAGAAGGAGTCTTACATGTGTGGGGTGTTACAACTGAA
AAGTCAAAAGAAGTTTGTATTACCATTTTCAATAGCAGTATAAAAGGctcgagtCCACCT
GGCCAGGAATCAGAATCAGCAGAATCTGCCAAAATAGGAGGAATAATTACTGTGTTT
TTGATAGCATTGGTCTTGACAAGCACCATAGTGACACTGAAATGGATTGGTTATATAT
GCTTAAGAAATAGCCTCCCCAAAGTCTTGAATTTTCATAACTTTTTAGCCTGGCCATT
TCCTAACCTGCCACCGTTGGAAGCCATGGATATGGTGGAGGTCATTTACATCAACAG
AAAGAAGAAAGTGTGGGATTATAATTATGATGATGAAAGTGATAGCGATACTGAGGC
AGCGCCCAGGACAAGTGGCGGTGGCTATACCATGCATGGACTGACTGTCAGGCCTC
TGGGTCAGGCCTCTGCCACCTCTACAGAATCCCAGTTGATAGACCCGGAGTCCGAGG
AGGAGCCTGACCTGCCTGAGGTTGATGTGGAGCTCCCCACGATGCCAAAGGACAGC
CCTCAGCAGTTGGAACTCTTGAGTGGGCCCTGTGAGAGGAGAAAGAGTCCACTCCAG
GACCCTTTTCCCGAAGAGGACTACAGCTCCACGGAGGGGTCTGGGGGCAGAATTAC
CTTCAATGTGGACTTAAACTCTGTGTTTTTGAGAGTTCTTGATGACGAGGACAGTGA
CGACTTAGAAGCCCCTCTGATGCTATCGTCTCATCTGGAAGAGATGGTTGACCCAGA
GGATCCTGATAATGTGCAATCAAACCATTTGCTGGCCAGCGGGGAAGGGACACAGCC
AACCTTTCCCAGCCCCTCTTCAGAGGGCCTGTGGTCCGAAGATGCTCCATCTGATCA
AAGTGACACTTCTGAGTCAGATGTTGACCTTGGGGATGGTTATATAATGAGATGA

④ IFNGR1-EX3-IFNAR2-TM-IN-240

ATGGCTCTCCTCTTTCTCCTACCCCTTGTCATGCAGGGTGTGAGCAGGGCTGAGATG
GGCACCGCGGATCTGGGGCCGTCCTCAGTGCCTACACCAACTAATGTTACAATTGAA
TCCTATAACATGAACCCTATCGTATATTGGGAGTACCAGATCATGCCACAGGTCCCT
GTTTTTACCGTAGAGGTAAAGAACTATGGTGTTAAGAATTCAGAATGGATTGATGCCT
GCATCAATATTTCTCATCATTATTGTAATATTTCTGATCATGTTGGTGATCCATCAAA
TTCTCTTTGGGTCAGAGTTAAAGCCAGGGTTGGACAAAAAGAATCTGCCTATGCAAA
GTCAGAAGAATTTGCTGTATGCCGAGATGGAAAAATTGGACCACCTAAACTGGATAT

CAGAAAGGAGGAGAAGCAAATCATGATTGACATATTTCACCCTTCAGTTTTTGTAAAT
GGAGACGAGCAGGAAGTCGATTATGATCCCGAAACTACCTGTTACATTAGGGTGTAC
AATGTGTATGTGAGAATGAACGGAAGTGAGATCCAGTATAAAATACTCACGCAGAAG
GAAGATGATTGTGACGAGATTCAGTGCCAGTTAGCGATTCCAGTATCCTCACTGAAT
TCTCAGTACTGTGTTTCAGCAGAAGGAGTCTTACATGTGTGGGGTGTTACAACTGAA
AAGTCAAAAGAAGTTTGTATTACCATTTTCAATAGCAGTATAAAAGGctcgagcGAATCT
GCCAAAATAGGAGGAATAATTACTGTGTTTTTGATAGCATTGGTCTTGACAAGCACCA
TAGTGACACTGAAATGGATTGGTTATATATGCTTAAGAAATAGCCTCCCCAAAGTCTT
GAATTTTCATAACTTTTTAGCCTGGCCATTTCCTAACCTGCCACCGTTGGAAGCCATG
GATATGGTGGAGGTCATTTACATCAACAGAAAGAAGAAAGTGTGGGATTATAATTAT
GATGATGAAAGTGATAGCGATACTGAGGCAGCGCCCAGGACAAGTGGCGGTGGCTA
TACCATGCATGGACTGACTGTCAGGCCTCTGGGTCAGGCCTCTGCCACCTCTACAGA
ATCCCAGTTGATAGACCCGGAGTCCGAGGAGGAGCCTGACCTGCCTGAGGTTGATG
TGGAGCTCCCCACGATGCCAAAGGACAGCCCTCAGCAGTTGGAACTCTTGAGTGGG
CCCTGTGAGAGGAGAAAGAGTCCACTCCAGGACCCTTTTCCCGAAGAGGACTACAGC
TCCACGGAGGGGTCTGGGGGCAGAATTACCTTCAATGTGGACTTAAACTCTGTGTTT
TTGAGAGTTCTTGATGACGAGGACAGTGACGACTTAGAAGCCCCTCTGATGCTATCG
TCTCATCTGGAAGAGATGGTTGACCCAGAGGATCCTGATAATGTGCAATCAAACCAT
TTGCTGGCCAGCGGGGAAGGGACACAGCCAACCTTTCCCAGCCCCTCTTCAGAGGG
CCTGTGGTCCGAAGATGCTCCATCTGATCAAAGTGACACTTCTGAGTCAGATGTTGA
CCTTGGGGATGGTTATATAATGAGATGA

⑤ IFNGR2-EX4-IFNAR1-TM-IN-394

ATGCGACCGACGCTGCTGTGGTCGCTGCTGCTGCTGCTCGGAGTCTTCGCCGCCGC
CGCCGCGGCCCCGCCAGACCCTCTTTCCCAGCTGCCCGCTCCTCAGCACCCGAAGA
TTCGCCTGTACAACGCAGAGCAGGTCCTGAGTTGGGAGCCAGTGGCCCTGAGCAAT
AGCACGAGGCCTGTTGTCTACCAAGTGCAGTTTAAATACACCGACAGTAAATGGTTC
ACGGCCGACATCATGTCCATAGGGGTGAATTGTACACAGATCACAGCAACAGAGTGT
GACTTCACTGCCGCCAGTCCCTCAGCAGGCTTCCCAATGGATTTCAATGTCACTCTA
CGCCTTCGAGCTGAGCTGGGAGCACTCCATTCTGCCTGGGTGACAATGCCTTGGTTT
CAACACTATCGGAATGTGACTGTCGGGCCTCCAGAAAACATTGAGGTGACCCCAGGA
GAAGGCTCCCTCATCATCAGGTTCTCCTCTCCCTTTGACATCGCTGATACCTCCACG
GCCTTTTTTTGTTATTATGTCCATTACTGGGAAAAAGGAGGAATCCAACAGGTCAAAG
GCCCTTTCAGAAGCAACTCCATTTCATTGGATAACTTAAAACCCTCCAGAGTGTACTG
TTTACAAGTCCAGGCACAACTGCTTTGGAACAAAAGTAACATCTTTAGAGTCGGGCA
TTTAAGCAACATctcgagtCCTAATTTGAAACCACTGACTGTATATTGTGTGAAAGCCAG
AGCACACACCATGGATGAAAAGCTGAATAAAAGCAGTGTTTTTAGTGACGCTGTATG
TGAGAAAACAAAACCAGGAAATACCTCTAAAATTTGGCTTATAGTTGGAATTTGTATT
GCATTATTTGCTCTCCCGTTTGTCATTTATGCTGCGAAAGTCTTCTTGAGATGCATCA
ATTATGTCTTCTTTCCATCACTTAAACCTTCTTCCAGTATAGATGAGTATTTCTCTGA
ACAGCCATTGAAGAATCTTCTGCTTTCAACTTCTGAGGAACAAATCGAAAAATGTTTC
ATAATTGAAAATATAAGCACAATTGCTACAGTAGAAGAAACTAATCAAACTGATGAAG
ATCATAAAAAATACAGTTCCCAAACTAGCCAAGATTCAGGAAATTATTCTAATGAAGA
TGAAAGCGAAAGTAAAACAAGTGAAGAACTACAGCAGGACTTTGTATGActctaga

⑥ IFNGR2-EX5-IFNAR1-TM-IN-394

ATGCGACCGACGCTGCTGTGGTCGCTGCTGCTGCTGCTCGGAGTCTTCGCCGCCGC
CGCCGCGGCCCCGCCAGACCCTCTTTCCCAGCTGCCCGCTCCTCAGCACCCGAAGA
TTCGCCTGTACAACGCAGAGCAGGTCCTGAGTTGGGAGCCAGTGGCCCTGAGCAAT
AGCACGAGGCCTGTTGTCTACCAAGTGCAGTTTAAATACACCGACAGTAAATGGTTC
ACGGCCGACATCATGTCCATAGGGGTGAATTGTACACAGATCACAGCAACAGAGTGT
GACTTCACTGCCGCCAGTCCCTCAGCAGGCTTCCCAATGGATTTCAATGTCACTCTA

CGCCTTCGAGCTGAGCTGGGAGCACTCCATTCTGCCTGGGTGACAATGCCTTGGTTT
CAACACTATCGGAATGTGACTGTCGGGCCTCCAGAAAACATTGAGGTGACCCCAGGA
GAAGGCTCCCTCATCATCAGGTTCTCCTCTCCCTTTGACATCGCTGATACCTCCACG
GCCTTTTTTTGTTATTATGTCCATTACTGGGAAAAAGGAGGAATCCAACAGGTCAAAG
GCCCTTTCAGAAGCAACTCCATTTCATTGGATAACTTAAAACCCTCCAGAGTGTACTG
TTTACAAGTCCAGGCACAACTGCTTTGGAACAAAAGTAACATCTTTAGAGTCGGGCA
TTTAAGCAACATATCTTGCTACGAAACAATGGCAGATGCCTCCACTGAGCTTCAGCAA
gtctcgagtCCTAATTTGAAACCACTGACTGTATATTGTGTGAAAGCCAGAGCACACACC
ATGGATGAAAAGCTGAATAAAAGCAGTGTTTTTAGTGACGCTGTATGTGAGAAAACA
AAACCAGGAAATACCTCTAAAATTTGGCTTATAGTTGGAATTTGTATTGCATTATTTG
CTCTCCCGTTTGTCATTTATGCTGCGAAAGTCTTCTTGAGATGCATCAATTATGTCTT
CTTTCCATCACTTAAACCTTCTTCCAGTATAGATGAGTATTTCTCTGAACAGCCATTG
AAGAATCTTCTGCTTTCAACTTCTGAGGAACAAATCGAAAAATGTTTCATAATTGAAA
ATATAAGCACAATTGCTACAGTAGAAGAAACTAATCAAACTGATGAAGATCATAAAAA
ATACAGTTCCCAAACTAGCCAAGATTCAGGAAATTATTCTAATGAAGATGAAAGCGAA
AGTAAAACAAGTGAAGAACTACAGCAGGACTTTGTATGActctaga

⑦ IFNGR2-EX5-IFNAR1-TM-IN-431

ATGCGACCGACGCTGCTGTGGTCGCTGCTGCTGCTGCTCGGAGTCTTCGCCGCCGC
CGCCGCGGCCCCGCCAGACCCTCTTTCCCAGCTGCCCGCTCCTCAGCACCCGAAGA
TTCGCCTGTACAACGCAGAGCAGGTCCTGAGTTGGGAGCCAGTGGCCCTGAGCAAT
AGCACGAGGCCTGTTGTCTACCAAGTGCAGTTTAAATACACCGACAGTAAATGGTTC
ACGGCCGACATCATGTCCATAGGGGTGAATTGTACACAGATCACAGCAACAGAGTGT
GACTTCACTGCCGCCAGTCCCTCAGCAGGCTTCCCAATGGATTTCAATGTCACTCTA
CGCCTTCGAGCTGAGCTGGGAGCACTCCATTCTGCCTGGGTGACAATGCCTTGGTTT
CAACACTATCGGAATGTGACTGTCGGGCCTCCAGAAAACATTGAGGTGACCCCAGGA
GAAGGCTCCCTCATCATCAGGTTCTCCTCTCCCTTTGACATCGCTGATACCTCCACG
GCCTTTTTTTGTTATTATGTCCATTACTGGGAAAAAGGAGGAATCCAACAGGTCAAAG
GCCCTTTCAGAAGCAACTCCATTTCATTGGATAACTTAAAACCCTCCAGAGTGTACTG
TTTACAAGTCCAGGCACAACTGCTTTGGAACAAAAGTAACATCTTTAGAGTCGGGCA
TTTAAGCAACATATCTTGCTACGAAACAATGGCAGATGCCTCCACTGAGCTTCAGCAA
gtctcgagtCCAGGAAATACCTCTAAAATTTGGCTTATAGTTGGAATTTGTATTGCATTAT
TTGCTCTCCCGTTTGTCATTTATGCTGCGAAAGTCTTCTTGAGATGCATCAATTATGT
CTTCTTTCCATCACTTAAACCTTCTTCCAGTATAGATGAGTATTTCTCTGAACAGCCA
TTGAAGAATCTTCTGCTTTCAACTTCTGAGGAACAAATCGAAAAATGTTTCATAATTG
AAAATATAAGCACAATTGCTACAGTAGAAGAAACTAATCAAACTGATGAAGATCATAA
AAAATACAGTTCCCAAACTAGCCAAGATTCAGGAAATTATTCTAATGAAGATGAAAGC
GAAAGTAAAACAAGTGAAGAACTACAGCAGGACTTTGTATGA

⑧ IFNGR2-EX5-IFNAR1-TM-IN-D83

ATGCGACCGACGCTGCTGTGGTCGCTGCTGCTGCTGCTCGGAGTCTTCGCCGCCGC
CGCCGCGGCCCCGCCAGACCCTCTTTCCCAGCTGCCCGCTCCTCAGCACCCGAAGA
TTCGCCTGTACAACGCAGAGCAGGTCCTGAGTTGGGAGCCAGTGGCCCTGAGCAAT
AGCACGAGGCCTGTTGTCTACCAAGTGCAGTTTAAATACACCGACAGTAAATGGTTC
ACGGCCGACATCATGTCCATAGGGGTGAATTGTACACAGATCACAGCAACAGAGTGT
GACTTCACTGCCGCCAGTCCCTCAGCAGGCTTCCCAATGGATTTCAATGTCACTCTA
CGCCTTCGAGCTGAGCTGGGAGCACTCCATTCTGCCTGGGTGACAATGCCTTGGTTT
CAACACTATCGGAATGTGACTGTCGGGCCTCCAGAAAACATTGAGGTGACCCCAGGA
GAAGGCTCCCTCATCATCAGGTTCTCCTCTCCCTTTGACATCGCTGATACCTCCACG
GCCTTTTTTTGTTATTATGTCCATTACTGGGAAAAAGGAGGAATCCAACAGGTCAAAG
GCCCTTTCAGAAGCAACTCCATTTCATTGGATAACTTAAAACCCTCCAGAGTGTACTG
TTTACAAGTCCAGGCACAACTGCTTTGGAACAAAAGTAACATCTTTAGAGTCGGGCA

TTTAAGCAACATATCTTGCTACGAAACAATGGCAGATGCCTCCACTGAGCTTCAGCAA
gtctcgagtCCAGGAAATACCTCTAAAATTTGGCTTATAGTTGGAATTTGTATTGCATTAT
TTGCTCTCCCGTTTGTCATTTATGCTGCGAAAGTCTTCTTGAGATGCATCAATTATGT
CTTCTTTCCATCACTTAAACCTTCTTCCAGTATAGATGAGTATTTCTCTGAACAGCCA
TTGAAGAATCTTCTGCTTTCAACTTCTGAGGAACAAATCGAAAAATGTTTCATAATTG
AAAATATAAGCACAATTGCTACAGTAGAAGAAACTAATCAAACTGATGAAGATCATAA
AAAATACAGTTCCCAAACTAGCCAAGATTCAGGAAATTATTCTAATGAAGATGAAAGC
GAAAGTAAAACAAGTgtcgactaa

7xISRE-IFNG

ggtaccacgcgtagatcccccGGGAAAGTGAAACTAGGGAAAGTGAAACTAGGGAAAGTGAA
ACTAGGGAAAGTGAAACTActcgaggaattcaagcttGGGAAAGTGAAACTAGGGAAAGTGA
AACTAGGGAAAGTGAAACTAcagcttagacactAGAGGGTATATAATGGAAGCTCGACTTC
CAGcttggcaatccggtactgttggtaaaggatccactagtaacggccgccagtgtgctggaattaagcttgaattc
tctcggaaacgATGAAATATACAAGTTATATCTTGGCTTTTCAGCTCTGCATCGTTTTGGG
TTCTCTTGGCTGTTACTGCCAGGACCCATATGTAAAAGAAGCAGAAAACCTTAAGAAA
TATTTTAATGCAGGTCATTCAGATGTAGCGGATAATGGAACTCTTTTCTTAGGCATTT
TGAAGAATTGGAAAGAGGAGAGTGACAGAAAAATAATGCAGAGCCAAATTGTCTCCT
TTTACTTCAAACTTTTTAAAAACTTTAAAGATGACCAGAGCATCCAAAAGAGTGTGGA
GACCATCAAGGAAGACATGAATGTCAAGTTTTTCAATAGCAACAAAAAGAAACGAGAT
GACTTCGAAAAGCTGACTAATTATTCGGTAACTGACTTGAATGTCCAACGCAAAGCAA
TACATGAACTCATCCAAGTGATGGCTGAACTGTCGCCAGCAGCTAAAACAGGGAAGC
GAAAAAGGAGTCAGATGCTGTTTCGAGGTCGAAGAGCATCCCAGTAA

Supplementary Table 8

DNA sequences in the plasmid constructs of Supplementary Fig 9.

sgRNA

CCAGATGATGGTCGTCCTCC

Target DNA sequence

CCAGATGATGGTCGTCCTCCTGG

Left arm-P2A-Puro^r

gcacaggggtgctgcaattaggatggggcaatgggagcttggagaaggggtgctagctaggaggaaaggcgcgtg
cgtggaggaacggcgcgtgcgcggaggggcggtgtgtgtgtcagaagAGGCGGCGCGTGCGTAGAGG
GGCGGTGAGAGCTAAGAGGGGCAGCGCGTGTGCAGAGGGGCGGTGTGACTTAGGAC
GGGGCGATGGCGGCTGAGAGGAGCTGCGCGTGCGCGAACATGTAACTGGTGGGATC
TGCGGCGGCTCCCAGATGATGGTCGTCCgactagtctctagaggatccggcgcaacaaacttctctc
tgctgaaacaagccggagatgtcgaagagaatcctggaccgACCGAGTACAAGCCCACGGTGCGCC
TCGCCACCCGCGACGACGTCCCCAGGGCCGTACGCACCCTCGCCGCCGCGTTCGCC
GACTACCCCGCCACGCGCCACACCGTCGATCCGGACCGCCACATCGAGCGGGTCAC
CGAGCTGCAAGAACTCTTCCTCACGCGCGTCGGGCTCGACATCGGCAAGGTGTGGG

TCGCGGACGACGGCGCCGCGGTGGCGGTCTGGACCACGCCGGAGAGCGTCGAAGC
GGGGGCGGTGTTCGCCGAGATCGGCCCGCGCATGGCCGAGTTGAGCGGTTCCCGGC
TGGCCGCGCAGCAACAGATGGAAGGCCTCCTGGCGCCGCACCGGCCCAAGGAGCCC
GCGTGGTTCCTGGCCACCGTCGGAGTCTCGCCCGACCACCAGGGCAAGGGTCTGGG
CAGCGCCGTCGTGCTCCCCGGAGTGGAGGCGGCCGAGCGCGCCGGGGTGCCCGCC
TTCCTGGAGACCTCCGCGCCCCGCAACCTCCCCTTCTACGAGCGGCTCGGCTTCACC
GTCACCGCCGACGTCGAGGTGCCCGAAGGACCGCGCACCTGGTGCATGACCCGCAA
GCCCGGTGCCTGA

Right arm

TCCTGGGCGCGACGACCCTAGTGCTCGTCGCCGTGGCGCCATGGGTGTTGTCCGCA
GCCGCAGgtgagaggcggggaggagagtcttggcgcagggcgggaggtagggcacgcagctgggctacggg
ggcggcgatgctgttgggggcgacagacgcccagtctgggaaaccttcggtccactttgccgcgccaaagattaaa
cccgacctgggctcgcaaatcaaccaggagaaagtggtgttctgggtcctctcttgccgcttgcctgtggccgtgtac
gggt

Supplementary Table 9

DNA sequences in the plasmid constructs of Supplementary Fig 10.

IL2RA

ATGGATTCATACCTGCTGATGTGGGGACTGCTCACGTTCATCATGGTGCCTGGCTGC
CAGGCAGAGCTCTGTGACGATGACCCGCCAGAGATCCCACACGCCACATTCAAAGCC
ATGGCCTACAAGGAAGGAACCATGTTGAACTGTGAATGCAAGAGAGGTTTCCGCAGA
ATAAAAAGCGGGTCACTCTATATGCTCTGTACAGGAAACTCTAGCCACTCGTCCTGG
GACAACCAATGTCAATGCACAAGCTCTGCCACTCGGAACACAACGAAACAAGTGACA
CCTCAACCTGAAGAACAGAAAGAAAGGAAAACCACAGAAATGCAAAGTCCAATGCAG
CCAGTGGACCAAGCGAGCCTTCCAGGTCACTGCAGGGAACCTCCACCATGGGAAAAT
GAAGCCACAGAGAGAATTTATCATTTCGTGGTGGGGCAGATGGTTTATTATCAGTGC
GTCCAGGGATACAGGGCTCTACACAGAGGTCCTGCTGAGAGCGTCTGCAAAATGACC
CACGGGAAGACAAGGTGGACCCAGCCCCAGCTCATATGCACAGGTGAAATGGAGAC
CAGTCAGTTTCCAGGTGAAGAGAAGCCTCAGGCAAGCCCCGAAGGCCGTCCTGAGA
GTGAGACTTCCTGCCTCGTCACAACAACAGATTTTCAAATACAGACAGAAATGGCTG
CAACCATGGAGACGTCCATATTTACAACAGAGTACCAGGTAGCAGTGGCCGGCTGTG
TTTTCCTGCTGATCAGCGTCCTCCTCCTGAGTGGGCTCACCTGGCAGCGGAGACAGA
GGAAGAGTAGAAGAACAATCTAG

① IL2RB-EX-IFNAR2-IN-231

ATGGCGGCCCCTGCTCTGTCCTGGCGTCTGCCCCTCCTCATCCTCCTCCTGCCCCTG
GCTACCTCTTGGGCATCTGCAGCGGTGAATGGCACTTCCCAGTTCACATGCTTCTAC
AACTCGAGAGCCAACATCTCCTGTGTCTGGAGCCAAGATGGGGCTCTGCAGGACACT
TCCTGCCAAGTCCATGCCTGGCCGGACAGACGGCGGTGGAACCAAACCTGTGAGCT
GCTCCCCGTGAGTCAAGCATCCTGGGCCTGCAACCTGATCCTCGGAGCCCCAGATTC
TCAGAAACTGACCACAGTTGACATCGTCACCCTGAGGGTGCTGTGCCGTGAGGGGG
TGCGATGGAGGGTGATGGCCATCCAGGACTTCAAGCCCTTTGAGAACCTTCGCCTGA
TGGCCCCCATCTCCCTCCAAGTTGTCCACGTGGAGACCCACAGATGCAACATAAGCT
GGGAAATCTCCCAAGCCTCCCACTACTTTGAAAGACACCTGGAGTTCGAGGCCCGGA
CGCTGTCCCCAGGCCACACCTGGGAGGAGGCCCCCCTGCTGACTCTCAAGCAGAAG

CAGGAATGGATCTGCCTGGAGACGCTCACCCCAGACACCCAGTATGAGTTTCAGGTG
CGGGTCAAGCCTCTGCAAGGCGAGTTCACGACCTGGAGCCCCTGGAGCCAGCCCCT
GGCCTTCAGGACAAAGCCTGCAGCCCTTGGGAAGGACACCgggtcgagtCCACCTGGCC
AGGAATCAGAATCAGCAGAATCTGCCAAAATAGGAGGAATAATTACTGTGTTTTTGAT
AGCATTGGTCTTGACAAGCACCATAGTGACACTGAAATGGATTGGTTATATATGCTTA
AGAAATAGCCTCCCCAAAGTCTTGAATTTTCATAACTTTTTAGCCTGGCCATTTCCTA
ACCTGCCACCGTTGGAAGCCATGGATATGGTGGAGGTCATTTACATCAACAGAAAGA
AGAAAGTGTGGGATTATAATTATGATGATGAAAGTGATAGCGATACTGAGGCAGCGC
CCAGGACAAGTGGCGGTGGCTATACCATGCATGGACTGACTGTCAGGCCTCTGGGT
CAGGCCTCTGCCACCTCTACAGAATCCCAGTTGATAGACCCGGAGTCCGAGGAGGAG
CCTGACCTGCCTGAGGTTGATGTGGAGCTCCCCACGATGCCAAAGGACAGCCCTCAG
CAGTTGGAACTCTTGAGTGGGCCCTGTGAGAGGAGAAAGAGTCCACTCCAGGACCCT
TTTCCCGAAGAGGACTACAGCTCCACGGAGGGGTCTGGGGGCAGAATTACCTTCAAT
GTGGACTTAAACTCTGTGTTTTTGAGAGTTCTTGATGACGAGGACAGTGACGACTTA
GAAGCCCCTCTGATGCTATCGTCTCATCTGGAAGAGATGGTTGACCCAGAGGATCCT
GATAATGTGCAATCAAACCATTTGCTGGCCAGCGGGGAAGGGACACAGCCAACCTTT
CCCAGCCCCTCTTCAGAGGGCCTGTGGTCCGAAGATGCTCCATCTGATCAAAGTGAC
ACTTCTGAGTCAGATGTTGACCTTGGGGATGGTTATATAATGAGATGA

② IL2RB-EX-IFNAR2-IN-240

ATGGCGGCCCCTGCTCTGTCCTGGCGTCTGCCCCTCCTCATCCTCCTCCTGCCCCTG
GCTACCTCTTGGGCATCTGCAGCGGTGAATGGCACTTCCCAGTTCACATGCTTCTAC
AACTCGAGAGCCAACATCTCCTGTGTCTGGAGCCAAGATGGGGCTCTGCAGGACACT
TCCTGCCAAGTCCATGCCTGGCCGGACAGACGGCGGTGGAACCAAACCTGTGAGCT
GCTCCCCGTGAGTCAAGCATCCTGGGCCTGCAACCTGATCCTCGGAGCCCCAGATTC
TCAGAAACTGACCACAGTTGACATCGTCACCCTGAGGGTGCTGTGCCGTGAGGGGG
TGCGATGGAGGGTGATGGCCATCCAGGACTTCAAGCCCTTTGAGAACCTTCGCCTGA
TGGCCCCCATCTCCCTCCAAGTTGTCCACGTGGAGACCCACAGATGCAACATAAGCT
GGGAAATCTCCCAAGCCTCCCACTACTTTGAAAGACACCTGGAGTTCGAGGCCCGGA
CGCTGTCCCCAGGCCACACCTGGGAGGAGGCCCCCCTGCTGACTCTCAAGCAGAAG
CAGGAATGGATCTGCCTGGAGACGCTCACCCCAGACACCCAGTATGAGTTTCAGGTG
CGGGTCAAGCCTCTGCAAGGCGAGTTCACGACCTGGAGCCCCTGGAGCCAGCCCCT
GGCCTTCAGGACAAAGCCTGCAGCCCTTGGGAAGGACACCgggtcgagcGAATCTGCCA
AAATAGGAGGAATAATTACTGTGTTTTTGATAGCATTGGTCTTGACAAGCACCATAGT
GACACTGAAATGGATTGGTTATATATGCTTAAGAAATAGCCTCCCCAAAGTCTTGAAT
TTTCATAACTTTTTAGCCTGGCCATTTCCTAACCTGCCACCGTTGGAAGCCATGGATA
TGGTGGAGGTCATTTACATCAACAGAAAGAAGAAAGTGTGGGATTATAATTATGATG
ATGAAAGTGATAGCGATACTGAGGCAGCGCCCAGGACAAGTGGCGGTGGCTATACC
ATGCATGGACTGACTGTCAGGCCTCTGGGTCAGGCCTCTGCCACCTCTACAGAATCC
CAGTTGATAGACCCGGAGTCCGAGGAGGAGCCTGACCTGCCTGAGGTTGATGTGGA
GCTCCCCACGATGCCAAAGGACAGCCCTCAGCAGTTGGAACTCTTGAGTGGGCCCTG
TGAGAGGAGAAAGAGTCCACTCCAGGACCCTTTTCCCGAAGAGGACTACAGCTCCAC
GGAGGGGTCTGGGGGCAGAATTACCTTCAATGTGGACTTAAACTCTGTGTTTTTGAG
AGTTCTTGATGACGAGGACAGTGACGACTTAGAAGCCCCTCTGATGCTATCGTCTCA
TCTGGAAGAGATGGTTGACCCAGAGGATCCTGATAATGTGCAATCAAACCATTTGCT
GGCCAGCGGGGAAGGGACACAGCCAACCTTTCCCAGCCCCTCTTCAGAGGGCCTGT
GGTCCGAAGATGCTCCATCTGATCAAAGTGACACTTCTGAGTCAGATGTTGACCTTG
GGGATGGTTATATAATGAGATGA

③ IL2RB-EX-IFNAR1-TM-IN-427

ATGGCGGCCCCTGCTCTGTCCTGGCGTCTGCCCCTCCTCATCCTCCTCCTGCCCCTG
GCTACCTCTTGGGCATCTGCAGCGGTGAATGGCACTTCCCAGTTCACATGCTTCTAC

AACTCGAGAGCCAACATCTCCTGTGTCTGGAGCCAAGATGGGGCTCTGCAGGACACT
TCCTGCCAAGTCCATGCCTGGCCGGACAGACGGCGGTGGAACCAAACCTGTGAGCT
GCTCCCCGTGAGTCAAGCATCCTGGGCCTGCAACCTGATCCTCGGAGCCCCAGATTC
TCAGAAACTGACCACAGTTGACATCGTCACCCTGAGGGTGCTGTGCCGTGAGGGGG
TGCGATGGAGGGTGATGGCCATCCAGGACTTCAAGCCCTTTGAGAACCTTCGCCTGA
TGGCCCCCATCTCCCTCCAAGTTGTCCACGTGGAGACCCACAGATGCAACATAAGCT
GGGAAATCTCCCAAGCCTCCCACTACTTTGAAAGACACCTGGAGTTCGAGGCCCGGA
CGCTGTCCCCAGGCCACACCTGGGAGGAGGCCCCCCTGCTGACTCTCAAGCAGAAG
CAGGAATGGATCTGCCTGGAGACGCTCACCCCAGACACCCAGTATGAGTTTCAGGTG
CGGGTCAAGCCTCTGCAAGGCGAGTTCACGACCTGGAGCCCCTGGAGCCAGCCCCT
GGCCTTCAGGACAAAGCCTGCAGCCCTTGGGAAGGACACCgggtcgagtGAGAAAACAA
AACCAGGAAATACCTCTAAAATTTGGCTTATAGTTGGAATTTGTATTGCATTATTTGC
TCTCCCGTTTGTCATTTATGCTGCGAAAGTCTTCTTGAGATGCATCAATTATGTCTTC
TTTCCATCACTTAAACCTTCTTCCAGTATAGATGAGTATTTCTCTGAACAGCCATTGA
AGAATCTTCTGCTTTCAACTTCTGAGGAACAAATCGAAAAATGTTTCATAATTGAAAA
TATAAGCACAATTGCTACAGTAGAAGAAACTAATCAAACTGATGAAGATCATAAAAAA
TACAGTTCCCAAACTAGCCAAGATTCAGGAAATTATTCTAATGAAGATGAAAGCGAAA
GTAAAACAAGTGAAGAACTACAGCAGGACTTTGTATGA

④　IL2RGB-EX-IFNAR1-TM-IN-431

ATGGCGGCCCCTGCTCTGTCCTGGCGTCTGCCCCTCCTCATCCTCCTCCTGCCCCTG
GCTACCTCTTGGGCATCTGCAGCGGTGAATGGCACTTCCCAGTTCACATGCTTCTAC
AACTCGAGAGCCAACATCTCCTGTGTCTGGAGCCAAGATGGGGCTCTGCAGGACACT
TCCTGCCAAGTCCATGCCTGGCCGGACAGACGGCGGTGGAACCAAACCTGTGAGCT
GCTCCCCGTGAGTCAAGCATCCTGGGCCTGCAACCTGATCCTCGGAGCCCCAGATTC
TCAGAAACTGACCACAGTTGACATCGTCACCCTGAGGGTGCTGTGCCGTGAGGGGG
TGCGATGGAGGGTGATGGCCATCCAGGACTTCAAGCCCTTTGAGAACCTTCGCCTGA
TGGCCCCCATCTCCCTCCAAGTTGTCCACGTGGAGACCCACAGATGCAACATAAGCT
GGGAAATCTCCCAAGCCTCCCACTACTTTGAAAGACACCTGGAGTTCGAGGCCCGGA
CGCTGTCCCCAGGCCACACCTGGGAGGAGGCCCCCCTGCTGACTCTCAAGCAGAAG
CAGGAATGGATCTGCCTGGAGACGCTCACCCCAGACACCCAGTATGAGTTTCAGGTG
CGGGTCAAGCCTCTGCAAGGCGAGTTCACGACCTGGAGCCCCTGGAGCCAGCCCCT
GGCCTTCAGGACAAAGCCTGCAGCCCTTGGGAAGGACACCgggtcgagtCCAGGAAATA
CCTCTAAAATTTGGCTTATAGTTGGAATTTGTATTGCATTATTTGCTCTCCCGTTTGT
CATTTATGCTGCGAAAGTCTTCTTGAGATGCATCAATTATGTCTTCTTTCCATCACTT
AAACCTTCTTCCAGTATAGATGAGTATTTCTCTGAACAGCCATTGAAGAATCTTCTGC
TTTCAACTTCTGAGGAACAAATCGAAAAATGTTTCATAATTGAAAATATAAGCACAAT
TGCTACAGTAGAAGAAACTAATCAAACTGATGAAGATCATAAAAAATACAGTTCCCAA
ACTAGCCAAGATTCAGGAAATTATTCTAATGAAGATGAAAGCGAAAGTAAAACAAGTG
AAGAACTACAGCAGGACTTTGTATGA

⑤　IL2RG-EX-IFNAR2-TM-IN-240

ATGTTGAAGCCATCATTACCATTCACATCCCTCTTATTCCTGCAGCTGCCCCTGCTGG
GAGTGGGGCTGAACACGACAATTCTGACGCCCAATGGGAATGAAGACACCACAGCTG
ATTTCTTCCTGACCACTATGCCCACTGACTCCCTCAGTGTTTCCACTCTGCCCCTCCC
AGAGGTTCAGTGTTTTGTGTTCAATGTCGAGTACATGAATTGCACTTGGAACAGCAG
CTCTGAGCCCCAGCCTACCAACCTCACTCTGCATTATTGGTACAAGAACTCGGATAA
TGATAAAGTCCAGAAGTGCAGCCACTATCTATTCTCTGAAGAAATCACTTCTGGCTGT
CAGTTGCAAAAAAAGGAGATCCACCTCTACCAAACATTTGTTGTTCAGCTCCAGGAC
CCACGGGAACCCAGGAGACAGGCCACACAGATGCTAAAACTGCAGAATCTGGTGATC
CCCTGGGCTCCAGAGAACCTAACACTTCACAAACTGAGTGAATCCCAGCTAGAACTG
AACTGGAACAACAGATTCTTGAACCACTGTTTGGAGCACTTGGTGCAGTACCGGACT

GACTGGGACCACAGCTGGACTGAACAATCAGTGGATTATAGACATAAGTTCTCCTTG
CCTAGTGTGGATGGGCAGAAACGCTACACGTTTCGTGTTCGGAGCCGCTTTAACCCA
CTCTGTGGAAGTGCTCAGCATTGGAGTGAATGGAGCCACCCAATCCACTGGGGGAG
CAATACTTCAAAAGAGAATCCTTTCCTGTTTGCATTGGAAGCCggctcgagcGAATCTGC
CAAAATAGGAGGAATAATTACTGTGTTTTTGATAGCATTGGTCTTGACAAGCACCATA
GTGACACTGAAATGGATTGGTTATATATGCTTAAGAAATAGCCTCCCCAAAGTCTTGA
ATTTTCATAACTTTTTAGCCTGGCCATTTCCTAACCTGCCACCGTTGGAAGCCATGGA
TATGGTGGAGGTCATTTACATCAACAGAAAGAAGAAAGTGTGGGATTATAATTATGAT
GATGAAAGTGATAGCGATACTGAGGCAGCGCCCAGGACAAGTGGCGGTGGCTATAC
CATGCATGGACTGACTGTCAGGCCTCTGGGTCAGGCCTCTGCCACCTCTACAGAATC
CCAGTTGATAGACCCGGAGTCCGAGGAGGAGCCTGACCTGCCTGAGGTTGATGTGG
AGCTCCCCACGATGCCAAAGGACAGCCCTCAGCAGTTGGAACTCTTGAGTGGGCCCT
GTGAGAGGAGAAAGAGTCCACTCCAGGACCCTTTTCCCGAAGAGGACTACAGCTCCA
CGGAGGGGTCTGGGGGCAGAATTACCTTCAATGTGGACTTAAACTCTGTGTTTTTGA
GAGTTCTTGATGACGAGGACAGTGACGACTTAGAAGCCCCTCTGATGCTATCGTCTC
ATCTGGAAGAGATGGTTGACCCAGAGGATCCTGATAATGTGCAATCAAACCATTTGC
TGGCCAGCGGGGAAGGGACACAGCCAACCTTTCCCAGCCCCTCTTCAGAGGGCCTG
TGGTCCGAAGATGCTCCATCTGATCAAAGTGACACTTCTGAGTCAGATGTTGACCTT
GGGGATGGTTATATAATGAGATGA

⑥ IL2RG-EX-IFNAR1-TM-IN-427

ATGTTGAAGCCATCATTACCATTCACATCCCTCTTATTCCTGCAGCTGCCCCTGCTGG
GAGTGGGGCTGAACACGACAATTCTGACGCCCAATGGGAATGAAGACACCACAGCTG
ATTTCTTCCTGACCACTATGCCCACTGACTCCCTCAGTGTTTCCACTCTGCCCCTCCC
AGAGGTTCAGTGTTTTGTGTTCAATGTCGAGTACATGAATTGCACTTGGAACAGCAG
CTCTGAGCCCCAGCCTACCAACCTCACTCTGCATTATTGGTACAAGAACTCGGATAA
TGATAAAGTCCAGAAGTGCAGCCACTATCTATTCTCTGAAGAAATCACTTCTGGCTGT
CAGTTGCAAAAAAAGGAGATCCACCTCTACCAAACATTTGTTGTTCAGCTCCAGGAC
CCACGGGAACCCAGGAGACAGGCCACACAGATGCTAAAACTGCAGAATCTGGTGATC
CCCTGGGCTCCAGAGAACCTAACACTTCACAAACTGAGTGAATCCCAGCTAGAACTG
AACTGGAACAACAGATTCTTGAACCACTGTTTGGAGCACTTGGTGCAGTACCGGACT
GACTGGGACCACAGCTGGACTGAACAATCAGTGGATTATAGACATAAGTTCTCCTTG
CCTAGTGTGGATGGGCAGAAACGCTACACGTTTCGTGTTCGGAGCCGCTTTAACCCA
CTCTGTGGAAGTGCTCAGCATTGGAGTGAATGGAGCCACCCAATCCACTGGGGGAG
CAATACTTCAAAAGAGAATCCTTTCCTGTTTGCATTGGAAGCCggctcgagtGAGAAAAC
AAAACCAGGAAATACCTCTAAAATTTGGCTTATAGTTGGAATTTGTATTGCATTATTT
GCTCTCCCGTTTGTCATTTATGCTGCGAAAGTCTTCTTGAGATGCATCAATTATGTCT
TCTTTCCATCACTTAAACCTTCTTCCAGTATAGATGAGTATTTCTCTGAACAGCCATT
GAAGAATCTTCTGCTTTCAACTTCTGAGGAACAAATCGAAAAATGTTTCATAATTGAA
AATATAAGCACAATTGCTACAGTAGAAGAAACTAATCAAACTGATGAAGATCATAAAA
AATACAGTTCCCAAACTAGCCAAGATTCAGGAAATTATTCTAATGAAGATGAAAGCGA
AAGTAAAACAAGTGAAGAACTACAGCAGGACTTTGTATGA

⑦ IL2RG-EX-IFNAR1-TM-IN-D83

ATGTTGAAGCCATCATTACCATTCACATCCCTCTTATTCCTGCAGCTGCCCCTGCTGG
GAGTGGGGCTGAACACGACAATTCTGACGCCCAATGGGAATGAAGACACCACAGCTG
ATTTCTTCCTGACCACTATGCCCACTGACTCCCTCAGTGTTTCCACTCTGCCCCTCCC
AGAGGTTCAGTGTTTTGTGTTCAATGTCGAGTACATGAATTGCACTTGGAACAGCAG
CTCTGAGCCCCAGCCTACCAACCTCACTCTGCATTATTGGTACAAGAACTCGGATAA
TGATAAAGTCCAGAAGTGCAGCCACTATCTATTCTCTGAAGAAATCACTTCTGGCTGT
CAGTTGCAAAAAAAGGAGATCCACCTCTACCAAACATTTGTTGTTCAGCTCCAGGAC
CCACGGGAACCCAGGAGACAGGCCACACAGATGCTAAAACTGCAGAATCTGGTGATC

CCCTGGGCTCCAGAGAACCTAACACTTCACAAACTGAGTGAATCCCAGCTAGAACTG
AACTGGAACAACAGATTCTTGAACCACTGTTTGGAGCACTTGGTGCAGTACCGGACT
GACTGGGACCACAGCTGGACTGAACAATCAGTGGATTATAGACATAAGTTCTCCTTG
CCTAGTGTGGATGGGCAGAAACGCTACACGTTTCGTGTTCGGAGCCGCTTTAACCCA
CTCTGTGGAAGTGCTCAGCATTGGAGTGAATGGAGCCACCCAATCCACTGGGGGAG
CAATACTTCAAAAGAGAATCCTTTCCTGTTTGCATTGGAAGCCggctcgagtCCAGGAAA
TACCTCTAAAATTTGGCTTATAGTTGGAATTTGTATTGCATTATTTGCTCTCCCGTTT
GTCATTTATGCTGCGAAAGTCTTCTTGAGATGCATCAATTATGTCTTCTTTCCATCAC
TTAAACCTTCTTCCAGTATAGATGAGTATTTCTCTGAACAGCCATTGAAGAATCTTCT
GCTTTCAACTTCTGAGGAACAAATCGAAAAATGTTTCATAATTGAAAATATAAGCACA
ATTGCTACAGTAGAAGAAACTAATCAAACTGATGAAGATCATAAAAAATACAGTTCCC
AAACTAGCCAAGATTCAGGAAATTATTCTAATGAAGATGAAAGCGAAAGTAAAACAAG
Tgtcgactaa

Supplementary Table 10

DNA sequence in the plasmid construct of Supplementary Fig 11.

ISREx10-IL-2

GGGAAAGTGAAACTAGGGAAAGTGAAACTAGGGAAAGTGAAACTAGGGAAAGTGAAA
CTActcgaggaattcaagcttGGGAAAGTGAAACTAGGGAAAGTGAAACTAGGGAAAGTGAA
ACTAcagcttGGGAAAGTGAAACTAGGGAAAGTGAAACTAGGGAAAGTGAAACTAcagctt
agacactAGAGGGTATATAATGGAAGCTCGACTTCCAGcttggcaatccggtactgttggtaaagg
atccgccaccATGTACAGGATGCAACTCCTGTCTTGCATTGCACTAAGTCTTGCACTTGT
CACAAACAGTGCACCTACTTCAAGTTCTACAAAGAAAACACAGCTACAACTGGAGCAT
TTACTGCTGGATTTACAGATGATTTTGAATGGAATTAATAATTACAAGAATCCCAAAC
TCACCAGGATGCTCACATTTAAGTTTTACATGCCCAAGAAGGCCACAGAACTGAAAC
ATCTTCAGTGTCTAGAAGAAGAACTCAAACCTCTGGAGGAAGTGCTAAATTTAGCTCA
AAGCAAAAACTTTCACTTAAGACCCAGGGACTTAATCAGCAATATCAACGTAATAGTT
CTGGAACTAAAGGGATCTGAAACAACATTCATGTGTGAATATGCTGATGAGACAGCA
ACCATTGTAGAATTTCTGAACAGATGGATTACCTTTTGTCAAAGCATCATCTCAACAC
TGACTTGA

Supplementary Table 11

DNA sequence in the plasmid construct of Supplementary Fig 12.

anti-NY-ESO-1-TCRa-E2A-TCRb-P2A-CD3e-T2A-CD3z

ATGGAGACCCTCTTGGGCCTGCTTATCCTTTGGCTGCAGCTGCAATGGGTGAGCAGC
AAACAGGAGGTGACACAGATTCCTGCAGCTCTGAGTGTCCCAGAAGGAGAAAACTTG
GTTCTCAACTGCAGTTTCACTGATAGCGCTATTTACAACCTCCAGTGGTTTAGGCAG
GACCCTGGGAAAGGTCTCACATCTCTGTTGCTTATTCAGTCAAGTCAGAGAGAGCAA
ACAAGTGGAAGACTTAATGCCTCGCTGGATAAATCATCAGGACGTAGTACTTTATACA
TTGCAGCTTCTCAGCCTGGTGACTCAGCCACCTACCTCTGTGCTGTGAGGCCCctgtac
GGAGGAAGCTACATACCTACATTTGGAAGAGGAACCAGCCTTATTGTTCATCCGTAT
ATCCAGAACCCggatccTGCCGTGTACCAGCTGAGAGACTCTAAATCCAGTGACAAGTC
TGTCTGCCTATTCACCGATTTTGATTCTCAAACAAATGTGTCACAAAGTAAGGATTCT
GATGTGTATATCACAGACAAAACTGTGCTAGACATGAGGTCTATGGACTTCAAGAGC
AACAGTGCTGTGGCCTGGAGCAACAAATCTGACTTTGCATGTGCAAACGCCTTCAAC
AACAGCATTATTCCAGAAGACACCTTCTTCCCCAGCCCAGAAAGTTCCTGTGATGTCA
AGCTGGTCGAGAAGAGCTTTGAAACAGATACGAACCTAAACTTTCAAAACCTGTCAG
TGATTGGGTTCCGAATCCTCCTCCTGAAAGTGGCCGGGTTTAATCTGCTCATGACGC

TGCGGCTGTGGTCCAGCgaattcgggtccggccaatgtactaactacgctttgttgaaactcgctggcgatg
ttgaaagtaaccccggtcctaagcttATGAGCATCGGCCTCCTGTGCTGTGCAGCCTTGTCTCT
CCTGTGGGCAGGTCCAGTGAATGCTGGTGTCACTCAGACCCCAAAATTCCAGGTCCT
GAAGACAGGACAGAGCATGACACTGCAGTGTGCCCAGGATATGAACCATGAATACAT
GTCCTGGTATCGACAAGACCCAGGCATGGGGCTGAGGCTGATTCATTACTCAGTTgc
catcGGTATCACTGACCAAGGAGAAGTCCCCAATGGCTACAATGTCTCCAGATCAACC
ACAGAGGATTTCCCGCTCAGGCTGCTGTCGGCTGCTCCCTCCCAGACATCTGTGTAC
TTCTGTGCCAGCAGTTACGTCGGGAACACCGGGGAGCTGTTTTTTGGAGAAGGCTCT
AGGCTGACCGTACTGGAGGACCTGAAAAACGTGTTCCCtccggaGGTCGCTGTGTTTG
AGCCATCAGAAGCAGAAATCTCCCACACCCAAAAGGCCACACTGGTGTGCCTGGCCA
CAGGCTTCTTCCCCGACCACGTGGAGCTGAGCTGGTGGGTGAATGGGAAGGAGGTG
CACAGTGGGGTCAGCACAGACCCGCAGCCCCTCAAGGAGCAGCCCGCCCTCAATGA
CTCCAGATACTGCCTGAGCAGCCGCCTGAGGGTCTCGGCCACCTTCTGGCAGAACC
CCCGCAACCACTTCCGCTGTCAAGTCCAGTTCTACGGGCTCTCGGAGAATGACGAGT
GGACCCAGGATAGGGCCAAACCCGTCACCCAGATCGTCAGCGCCGAGGCCTGGGGT
AGAGCAGACTGTGGCTTTACCTCGGTGTCCTACCAGCAAGGGGTCCTGTCTGCCACC
ATCCTCTATGAGATCCTGCTAGGGAAGGCCACCCTGTATGCTGTGCTGGTCAGCGCC
CTTGTGTTGATGGCCATGGTCAAGAGAAAGGATTTCacgcgtggctccggcgccacgaacttct
ctctgttaaagcaagcaggagacgtggaagaaaaccccggtcccggtaccATGCAGTCGGGCACTCACT
GGAGAGTTCTGGGCCTCTGCCTCTTATCAGTTGGCGTTTGGGGGCAAGATGGTAATG
AAGAAATGGGTGGTATTACACAGACACCATATAAAGTCTCCATCTCTGGAACCACAG
TAATATTGACATGCCCTCAGTATCCTGGATCTGAAATACTATGGCAACACAATGATAA
AAACATAGGCGGTGATGAGGATGATAAAAACATAGGCAGTGATGAGGATCACCTGTC
ACTGAAGGAATTTTCAGAATTGGAGCAAAGTGGTTATTATGTCTGCTACCCCAGAGG
AAGCAAACCAGAAGATGCGAACTTTTATCTCTACCTGAGGGCAAGAGTGTGTGAGAA
CTGCATGGAGATGGATGTGATGTCGGTGGCCACAATTGTCATAGTGGACATCTGCAT
CACTGGGGGCTTGCTGCTGCTGGTTTACTACTGGAGCAAGAATAGAAAGGCCAAGGC
CAAGCCTGTGACACGAGGAGCGGGTGCTGGCGGCAGGCAAAGGGGACAAAACAAGG
AGAGGCCACCACCTGTTCCCAACCCAGACTATGAGCCCATCCGGAAAGGCCAGCGG
GACCTGTATTCTGGCCTGAATCAGAGACGCATCggatccgggtccggcgagggcagaggaagtc
tgctaacatgcggtgacgtcgaggagaatcctggcccaaccggtATGAAGTGGAAGGCGCTTTTCACC
GCGGCCATCCTGCAGGCACAGTTGCCGATTACAGAGGCACAGAGCTTTGGCCTGCT
GGACCCCAAACTCTGCTACCTGCTGGATGGAATCCTCTTCATCTATGGTGTCATTCT
CACTGCCTTGTTCCTGAGAGTGAAGTTCAGCAGGAGCGCAGACGCCCCCGCGTACCA
GCAGGGCCAGAACCAGCTCTATAACGAGCTCAATCTAGGACGAAGAGAGGAGTACGA
TGTTTTGGACAAGAGACGTGGCCGGGACCCTGAGATGGGGGGAAAGCCGCAGAGAA
GGAAGAACCCTCAGGAAGGCCTGTACAATGAACTGCAGAAAGATAAGATGGCGGAGG
CCTACAGTGAGATTGGGATGAAAGGCGAGCGCCGGAGGGGCAAGGGGCACGATGGC
CTTTACCAGGGTCTCAGTACAGCCACCAAGGACACCTACGACGCCCTTCACATGCAG
GCCCTGCCCCCTCGCTGA

Supplementary Table 12

DNA sequences in the plasmid constructs of Supplementary Fig 15.

anti-CD3e-scFv-Sc

ATGCCGCTGCTGCTACTGCTGCCCCTGCTGTGGGCAGGTGCCCTGGCTATGGACCC
AtccggaGTCCAGCTGCAGCAGTCTGGGGCTGAACTGGCAAGACCTGGGGCCTCAGTG
AAGATGTCCTGCAAGGCTTCTGGCTACACCTTTACTAGGTACACGATGCACTGGGTA
AAACAGAGGCCTGGACAGGGTCTGGAATGGATTGGATACATTAATCCTAGCCGTGGT
TATACTAATTACAATCAGAAGTTCAAGGACAAGGCCACATTGACTACAGACAAATCCT
CCAGCACAGCCTACATGCAACTGAGCAGCCTGACATCTGAGGACTCTGCAGTCTATT
ACTGTGCAAGATATTATGATGATCATTACTGCCTTGACTACTGGGGCCAAGGCACCA
CTCTCACAGTCTCCTCAggatccggtggtggtggtagcggtggtggcggttctggcggtggagggtcgacc
GATATTGTTCTCACCCAGTCTCCAGCAATCATGTCTGCATCTCCAGGGGAGAAGGTC
ACCATGACCTGCAGTGCCAGCTCAAGTGTAAGTTACATGAACTGGTATCAGCAGAAG

TCAGGCACCTCCCCCAAAAGATGGATTTATGACACATCCAAACTGGCTTCTGGAGTC
CCTGCTCACTTCAGGGGCAGTGGGTCTGGGACCTCTTACTCTCTCACAATCAGCGGC
ATGGAGGCTGAAGATGCTGCCACTTATTACTGCCAGCAGTGGAGTAGTAACCCATTC
ACGTTCGGCTCGGGGACAAAGTTGGAAATAaaggggccctgtacaggtggtggaggctcgagcta
a

anti-CD3e-scFv-TM
ATGCCGCTGCTGCTACTGCTGCCCCTGCTGTGGGCAGGTGCCCTGGCTATGGACCC
AtccggaGTCCAGCTGCAGCAGTCTGGGGCTGAACTGGCAAGACCTGGGGCCTCAGTG
AAGATGTCCTGCAAGGCTTCTGGCTACACCTTTACTAGGTACACGATGCACTGGGTA
AAACAGAGGCCTGGACAGGGTCTGGAATGGATTGGATACATTAATCCTAGCCGTGGT
TATACTAATTACAATCAGAAGTTCAAGGACAAGGCCACATTGACTACAGACAAATCCT
CCAGCACAGCCTACATGCAACTGAGCAGCCTGACATCTGAGGACTCTGCAGTCTATT
ACTGTGCAAGATATTATGATGATCATTACTGCCTTGACTACTGGGGCCAAGGCACCA
CTCTCACAGTCTCCTCAggatccggtggtggtggtagcggtggtggcggttctggcggtggagggtcgacc
GATATTGTTCTCACCCAGTCTCCAGCAATCATGTCTGCATCTCCAGGGGAGAAGGTC
ACCATGACCTGCAGTGCCAGCTCAAGTGTAAGTTACATGAACTGGTATCAGCAGAAG
TCAGGCACCTCCCCCAAAAGATGGATTTATGACACATCCAAACTGGCTTCTGGAGTC
CCTGCTCACTTCAGGGGCAGTGGGTCTGGGACCTCTTACTCTCTCACAATCAGCGGC
ATGGAGGCTGAAGATGCTGCCACTTATTACTGCCAGCAGTGGAGTAGTAACCCATTC
ACGTTCGGCTCGGGGACAAAGTTGGAAATAaaggggccctgtacaggtggtggaggctcgactCC
TCTGACGTCCATCATCTCTGCGGTGGTTGGCATTCTGCTGGTCGTGGTCTTGGGGGT
GGTCTTTGGGATCCTCATCAAGCGACGGCAGCAGAAGATCCGGAAGTActcgagctaa

B7-1

ATGGGCCACACACGGAGGCAGGGAACATCACCATCCAAGTGTCCATACCTCAATTTC
TTTCAGCTCTTGGTGCTGGCTGGTCTTTCTCACTTCTGTTCAGGTGTTATCCACGTG
ACCAAGGAAGTGAAAGAAGTGGCAACGCTGTCCTGTGGTCACAATGTTTCTGTTGAA
GAGCTGGCACAAACTCGCATCTACTGGCAAAAGGAGAAGAAAATGGTGCTGACTATG
ATGTCTGGGGACATGAATATATGGCCCGAGTACAAGAACCGGACCATCTTTGATATC
ACTAATAACCTCTCCATTGTGATCCTGGCTCTGCGCCCATCTGACGAGGGCACATAC
GAGTGTGTTGTTCTGAAGTATGAAAAAGACGCTTTCAAGCGGGAACACCTGGCTGAA
GTGACGTTATCAGTCAAAGCTGACTTCCCTACACCTAGTATATCTGACTTTGAAATTC
CAACTTCTAATATTAGAAGGATAATTTGCTCAACCTCTGGAGGTTTTCCAGAGCCTCA
CCTCTCCTGGTTGGAAAATGGAGAAGAATTAAATGCCATCAACACAACAGTTTCCCAA
GATCCTGAAACTGAGCTCTATGCTGTTAGCAGCAAACTGGATTTCAATATGACAACCA
ACCACAGCTTCATGTGTCTCATCAAGTATGGACATTTAAGAGTGAATCAGACCTTCAA
CTGGAATACAACCAAGCAAGAGCATTTTCCTGATAACCTGCTCCCATCCTGGGCCAT
TACCTTAATCTCAGTAAATGGAATTTTTGTGATATGCTGCCTGACCTACTGCTTTGCC
CCAAGATGCAGAGAGAGAAGGAGGAATGAGAGATTGAGAAGGGAAAGTGTACGCCC
TGTATAA

Supplementary Table 13

DNA sequences in the plasmid constructs of Supplementary Fig 18.

① IFNGR1-EX-TM-IFNAR2-IN-265

ATGGCTCTCCTCTTTCTCCTACCCCTTGTCATGCAGGGTGTGAGCAGGGCTGAGATG
GGCACCGCGGATCTGGGGCCGTCCTCAGTGCCTACACCAACTAATGTTACAATTGAA
TCCTATAACATGAACCCTATCGTATATTGGGAGTACCAGATCATGCCACAGGTCCCT

GTTTTTACCGTAGAGGTAAAGAACTATGGTGTTAAGAATTCAGAATGGATTGATGCCT
GCATCAATATTTCTCATCATTATTGTAATATTTCTGATCATGTTGGTGATCCATCAAA
TTCTCTTTGGGTCAGAGTTAAAGCCAGGGTTGGACAAAAAGAATCTGCCTATGCAAA
GTCAGAAGAATTTGCTGTATGCCGAGATGGAAAAATTGGACCACCTAAACTGGATAT
CAGAAAGGAGGAGAAGCAAATCATGATTGACATATTTCACCCTTCAGTTTTTGTAAAT
GGAGACGAGCAGGAAGTCGATTATGATCCCGAAACTACCTGTTACATTAGGGTGTAC
AATGTGTATGTGAGAATGAACGGAAGTGAGATCCAGTATAAAATACTCACGCAGAAG
GAAGATGATTGTGACGAGATTCAGTGCCAGTTAGCGATTCCAGTATCCTCACTGAAT
TCTCAGTACTGTGTTTCAGCAGAAGGAGTCTTACATGTGTGGGGTGTTACAACTGAA
AAGTCAAAAGAAGTTTGTATTACCATTTTCAATAGCAGTATAAAAGGTTCTCTTTGGA
TTCCAGTTGTTGCTGCTTTACTACTCTTTCTAGTGCTTAGCCTGGTATTCATCTGTTT
TTATATTAAGAAAggctcgagcAAATGGATTGGTTATATATGCTTAAGAAATAGCCTCCC
CAAAGTCTTGAATTTTCATAACTTTTTAGCCTGGCCATTTCCTAACCTGCCACCGTTG
GAAGCCATGGATATGGTGGAGGTCATTTACATCAACAGAAAGAAGAAAGTGTGGGAT
TATAATTATGATGATGAAAGTGATAGCGATACTGAGGCAGCGCCCAGGACAAGTGGC
GGTGGCTATACCATGCATGGACTGACTGTCAGGCCTCTGGGTCAGGCCTCTGCCACC
TCTACAGAATCCCAGTTGATAGACCCGGAGTCCGAGGAGGAGCCTGACCTGCCTGAG
GTTGATGTGGAGCTCCCCACGATGCCAAAGGACAGCCCTCAGCAGTTGGAACTCTTG
AGTGGGCCCTGTGAGAGGAGAAAGAGTCCACTCCAGGACCCTTTTCCCGAAGAGGA
CTACAGCTCCACGGAGGGGTCTGGGGGCAGAATTACCTTCAATGTGGACTTAAACTC
TGTGTTTTTGAGAGTTCTTGATGACGAGGACAGTGACGACTTAGAAGCCCCTCTGAT
GCTATCGTCTCATCTGGAAGAGATGGTTGACCCAGAGGATCCTGATAATGTGCAATC
AAACCATTTGCTGGCCAGCGGGGAAGGGACACAGCCAACCTTTCCCAGCCCCTCTTC
AGAGGGCCTGTGGTCCGAAGATGCTCCATCTGATCAAAGTGACACTTCTGAGTCAGA
TGTTGACCTTGGGGATGGTTATATAATGAGATGA

② IFNGR1-EX-TM-IFNAR1-IN-D62

ATGGCTCTCCTCTTTCTCCTACCCCTTGTCATGCAGGGTGTGAGCAGGGCTGAGATG
GGCACCGCGGATCTGGGGCCGTCCTCAGTGCCTACACCAACTAATGTTACAATTGAA
TCCTATAACATGAACCCTATCGTATATTGGGAGTACCAGATCATGCCACAGGTCCCT
GTTTTTACCGTAGAGGTAAAGAACTATGGTGTTAAGAATTCAGAATGGATTGATGCCT
GCATCAATATTTCTCATCATTATTGTAATATTTCTGATCATGTTGGTGATCCATCAAA
TTCTCTTTGGGTCAGAGTTAAAGCCAGGGTTGGACAAAAAGAATCTGCCTATGCAAA
GTCAGAAGAATTTGCTGTATGCCGAGATGGAAAAATTGGACCACCTAAACTGGATAT
CAGAAAGGAGGAGAAGCAAATCATGATTGACATATTTCACCCTTCAGTTTTTGTAAAT
GGAGACGAGCAGGAAGTCGATTATGATCCCGAAACTACCTGTTACATTAGGGTGTAC
AATGTGTATGTGAGAATGAACGGAAGTGAGATCCAGTATAAAATACTCACGCAGAAG
GAAGATGATTGTGACGAGATTCAGTGCCAGTTAGCGATTCCAGTATCCTCACTGAAT
TCTCAGTACTGTGTTTCAGCAGAAGGAGTCTTACATGTGTGGGGTGTTACAACTGAA
AAGTCAAAAGAAGTTTGTATTACCATTTTCAATAGCAGTATAAAAGGTTCTCTTTGGA
TTCCAGTTGTTGCTGCTTTACTACTCTTTCTAGTGCTTAGCCTGGTATTCATCTGTTT
TTATATTAAGAAAggctcgagcTTGAGATGCATCAATTATGTCTTCTTTCCATCACTTAAA
CCTTCTTCCAGTATAGATGAGTATTTCTCTGAACAGCCATTGAAGAATCTTCTGCTTT
CAACTTCTGAGGAACAAATCGAAAAATGTTTCATAATTGAAAATATAAGCACAATTGC
TACAGTAGAAGAAACTAATCAAACTGATGAAGATCATAAAAAATACAGTTCCCAAACT
AGCCAAGATTCAGGAAATtaa

Supplementary Table 14

DNA sequences in the plasmid constructs of Supplementary Fig 19.

① IFNGR1-EX-TM-SmBiT

ATGGCTCTCCTCTTTCTCCTACCCCTTGTCATGCAGGGTGTGAGCAGGGCTGAGATG
GGCACCGCGGATCTGGGGCCGTCCTCAGTGCCTACACCAACTAATGTTACAATTGAA
TCCTATAACATGAACCCTATCGTATATTGGGAGTACCAGATCATGCCACAGGTCCCT
GTTTTTACCGTAGAGGTAAAGAACTATGGTGTTAAGAATTCAGAATGGATTGATGCCT
GCATCAATATTTCTCATCATTATTGTAATATTTCTGATCATGTTGGTGATCCATCAAA
TTCTCTTTGGGTCAGAGTTAAAGCCAGGGTTGGACAAAAAGAATCTGCCTATGCAAA
GTCAGAAGAATTTGCTGTATGCCGAGATGGAAAAATTGGACCACCTAAACTGGATAT
CAGAAAGGAGGAGAAGCAAATCATGATTGACATATTTCACCCTTCAGTTTTTGTAAAT
GGAGACGAGCAGGAAGTCGATTATGATCCCGAAACTACCTGTTACATTAGGGTGTAC
AATGTGTATGTGAGAATGAACGGAAGTGAGATCCAGTATAAAATACTCACGCAGAAG
GAAGATGATTGTGACGAGATTCAGTGCCAGTTAGCGATTCCAGTATCCTCACTGAAT
TCTCAGTACTGTGTTTCAGCAGAAGGAGTCTTACATGTGTGGGGTGTTACAACTGAA
AAGTCAAAAGAAGTTTGTATTACCATTTTCAATAGCAGTATAAAAGGTTCTCTTTGGA
TTCCAGTTGTTGCTGCTTTACTACTCTTTCTAGTGCTTAGCCTGGTATTCATCTGTTT
TTATATTAAGAAAggctcgagcggtGTGACCGGCTACCGGCTGTTCGAGGAGATTCTGtaa

② IFNGR1-EX-TM-Linker-SmBiT

ATGGCTCTCCTCTTTCTCCTACCCCTTGTCATGCAGGGTGTGAGCAGGGCTGAGATG
GGCACCGCGGATCTGGGGCCGTCCTCAGTGCCTACACCAACTAATGTTACAATTGAA
TCCTATAACATGAACCCTATCGTATATTGGGAGTACCAGATCATGCCACAGGTCCCT
GTTTTTACCGTAGAGGTAAAGAACTATGGTGTTAAGAATTCAGAATGGATTGATGCCT
GCATCAATATTTCTCATCATTATTGTAATATTTCTGATCATGTTGGTGATCCATCAAA
TTCTCTTTGGGTCAGAGTTAAAGCCAGGGTTGGACAAAAAGAATCTGCCTATGCAAA
GTCAGAAGAATTTGCTGTATGCCGAGATGGAAAAATTGGACCACCTAAACTGGATAT
CAGAAAGGAGGAGAAGCAAATCATGATTGACATATTTCACCCTTCAGTTTTTGTAAAT
GGAGACGAGCAGGAAGTCGATTATGATCCCGAAACTACCTGTTACATTAGGGTGTAC
AATGTGTATGTGAGAATGAACGGAAGTGAGATCCAGTATAAAATACTCACGCAGAAG
GAAGATGATTGTGACGAGATTCAGTGCCAGTTAGCGATTCCAGTATCCTCACTGAAT
TCTCAGTACTGTGTTTCAGCAGAAGGAGTCTTACATGTGTGGGGTGTTACAACTGAA
AAGTCAAAAGAAGTTTGTATTACCATTTTCAATAGCAGTATAAAAGGTTCTCTTTGGA
TTCCAGTTGTTGCTGCTTTACTACTCTTTCTAGTGCTTAGCCTGGTATTCATCTGTTT
TTATATTAAGAAAggctcgagcGGTGGTGGAGGGTCAGGAGGTGGAGGGtcgagcGGTGG
TGGAGGGTCAGGAGGTGGAGGGtcgagcGGTGGTGGAGGGTCAGGAGGTGGAGGGtcg
agcgccATGGGCTCCGGAGGCGGATCCGGTGGGtcgagcggtGTGACCGGCTACCGGCT
GTTCGAGGAGATTCTGTAA

③ IFNGR1-EX-TM-LgBiT

ATGGCTCTCCTCTTTCTCCTACCCCTTGTCATGCAGGGTGTGAGCAGGGCTGAGATG
GGCACCGCGGATCTGGGGCCGTCCTCAGTGCCTACACCAACTAATGTTACAATTGAA
TCCTATAACATGAACCCTATCGTATATTGGGAGTACCAGATCATGCCACAGGTCCCT
GTTTTTACCGTAGAGGTAAAGAACTATGGTGTTAAGAATTCAGAATGGATTGATGCCT
GCATCAATATTTCTCATCATTATTGTAATATTTCTGATCATGTTGGTGATCCATCAAA
TTCTCTTTGGGTCAGAGTTAAAGCCAGGGTTGGACAAAAAGAATCTGCCTATGCAAA
GTCAGAAGAATTTGCTGTATGCCGAGATGGAAAAATTGGACCACCTAAACTGGATAT
CAGAAAGGAGGAGAAGCAAATCATGATTGACATATTTCACCCTTCAGTTTTTGTAAAT
GGAGACGAGCAGGAAGTCGATTATGATCCCGAAACTACCTGTTACATTAGGGTGTAC
AATGTGTATGTGAGAATGAACGGAAGTGAGATCCAGTATAAAATACTCACGCAGAAG
GAAGATGATTGTGACGAGATTCAGTGCCAGTTAGCGATTCCAGTATCCTCACTGAAT
TCTCAGTACTGTGTTTCAGCAGAAGGAGTCTTACATGTGTGGGGTGTTACAACTGAA
AAGTCAAAAGAAGTTTGTATTACCATTTTCAATAGCAGTATAAAAGGTTCTCTTTGGA
TTCCAGTTGTTGCTGCTTTACTACTCTTTCTAGTGCTTAGCCTGGTATTCATCTGTTT
TTATATTAAGAAAggctcgagcggcGTCTTCACACTCGAAGATTTCGTTGGGGACTGGGA

ACAGACAGCCGCCTACAACCTGGACCAAGTCCTTGAACAGGGAGGTGTGTCCAGTTT
GCTGCAGAATCTCGCCGTGTCCGTAACTCCGATCCAAAGGATTGTCCGTAGCGGTGA
AAATGCCCTGAAGATCGACATCCATGTCATCATCCCGTATGAAGGTCTGAGCGCCGA
CCAAATGGCCCAGATCGAAGAGGTGTTTAAGGTGGTGTACCCTGTGGATGATCATCA
CTTTAAGGTGATCCTGCCCTATGGCACACTGGTAATCGACGGGGTTACGCCGAACAT
GCTGAACTATTTCGGACGGCCGTATGAAGGCATCGCCGTGTTCGACGGCAAAAAGAT
CACTGTAACAGGGACCCTGTGGAACGGCAACAAAATTATCGACGAGCGCCTGATCAC
CCCCGACGGCTCCATGCTGTTCCGAGTAACCATCAACAGTTAA

④ IFNGR1-EX-TM-Linker-LgBiT

ATGGCTCTCCTCTTTCTCCTACCCCTTGTCATGCAGGGTGTGAGCAGGGCTGAGATG
GGCACCGCGGATCTGGGGCCGTCCTCAGTGCCTACACCAACTAATGTTACAATTGAA
TCCTATAACATGAACCCTATCGTATATTGGGAGTACCAGATCATGCCACAGGTCCCT
GTTTTTACCGTAGAGGTAAAGAACTATGGTGTTAAGAATTCAGAATGGATTGATGCCT
GCATCAATATTTCTCATCATTATTGTAATATTTCTGATCATGTTGGTGATCCATCAAA
TTCTCTTTGGGTCAGAGTTAAAGCCAGGGTTGGACAAAAAGAATCTGCCTATGCAAA
GTCAGAAGAATTTGCTGTATGCCGAGATGGAAAAATTGGACCACCTAAACTGGATAT
CAGAAAGGAGGAGAAGCAAATCATGATTGACATATTTCACCCTTCAGTTTTTGTAAAT
GGAGACGAGCAGGAAGTCGATTATGATCCCGAAACTACCTGTTACATTAGGGTGTAC
AATGTGTATGTGAGAATGAACGGAAGTGAGATCCAGTATAAAATACTCACGCAGAAG
GAAGATGATTGTGACGAGATTCAGTGCCAGTTAGCGATTCCAGTATCCTCACTGAAT
TCTCAGTACTGTGTTTCAGCAGAAGGAGTCTTACATGTGTGGGGTGTTACAACTGAA
AAGTCAAAAGAAGTTTGTATTACCATTTTCAATAGCAGTATAAAAGGTTCTCTTTGGA
TTCCAGTTGTTGCTGCTTTACTACTCTTTCTAGTGCTTAGCCTGGTATTCATCTGTTT
TTATATTAAGAAAggctcgagcGGTGGTGGAGGGTCAGGAGGTGGAGGGtcgagcGGTGG
TGGAGGGTCAGGAGGTGGAGGGtcgagcGGTGGTGGAGGGTCAGGAGGTGGAGGGtcg
agcgccATGGCCAGATCTGGGtcgagcggcGTCTTCACACTCGAAGATTTCGTTGGGGAC
TGGGAACAGACAGCCGCCTACAACCTGGACCAAGTCCTTGAACAGGGAGGTGTGTCC
AGTTTGCTGCAGAATCTCGCCGTGTCCGTAACTCCGATCCAAAGGATTGTCCGTAGC
GGTGAAAATGCCCTGAAGATCGACATCCATGTCATCATCCCGTATGAAGGTCTGAGC
GCCGACCAAATGGCCCAGATCGAAGAGGTGTTTAAGGTGGTGTACCCTGTGGATGAT
CATCACTTTAAGGTGATCCTGCCCTATGGCACACTGGTAATCGACGGGGTTACGCCG
AACATGCTGAACTATTTCGGACGGCCGTATGAAGGCATCGCCGTGTTCGACGGCAAA
AAGATCACTGTAACAGGGACCCTGTGGAACGGCAACAAAATTATCGACGAGCGCCTG
ATCACCCCCGACGGCTCCATGCTGTTCCGAGTAACCATCAACAGTTAA
